# Relationship between nine triglyceride-glucose-related indices and cardiometabolic multimorbidity incidence in patients with cardiovascular-kidney-metabolic syndrome stage 0–3: a nationwide prospective cohort study

**DOI:** 10.1186/s12933-026-03077-4

**Published:** 2026-01-12

**Authors:** Tonglong Jin, Xiaogang Tang, Yang Han, Haiqing Fan, Qi Qin, Hui Jiang, Zhaoyao Chen, Wenlei Li, Yuan Zhu, Minghua Wu

**Affiliations:** 1 Department of Neurology, Affiliated Hospital of Nanjing University of Chinese Medicine, 155 Hanzhong Road, Jiangsu Province 210029 Nanjing, China; 2 Department of Neurology, Jiangsu Province Hospital of Chinese Medicine, Nanjing, 210029 Jiangsu Province China; 3 Department of Neurology, Changshu Hospital Affiliated to Nanjing University of Chinese Medicine, Suzhou, 215500 Jiangsu Province China

**Keywords:** TyG-related indices, CKM syndrome, CMM, CHARLS, TyG-CVAI

## Abstract

**Background:**

Cardiovascular-kidney-metabolic (CKM) syndrome integrates metabolic, renal, and cardiovascular disease risk. While increasing evidence suggests that triglyceride-glucose (TyG)-related indices are associated with the future risk of cardiometabolic multimorbidity (CMM), their link to CMM in CKM syndrome has not been established.

**Methods:**

This study analyzed participants with CKM syndrome stage 0–3 from the China Health and Retirement Longitudinal Study (CHARLS) from 2011 to 2020. We used Cox regression analysis, restricted cubic spline (RCS) curves, and Kaplan–Meier (K–M) survival curves to evaluate the relationship between TyG-related indices and CMM risk in patients with CKM stage 0–3 syndrome. Receiver operating characteristic (ROC) curves, net reclassification improvement (NRI), and integrated discrimination improvement (IDI) analyses were used to assess the predictive performance of the TyG-related indices for CMM.

**Results:**

During a median follow-up of 9 years, 652 participants (9.5%) developed CMM. The fully adjusted model revealed an elevated CMM risk across the highest quartiles of all indices, with hazard increases ranging from 72 to over 200%. A linear dose-response relationship was observed for most indices, except for triglyceride glucose-a body shape index (TyG-ABSI) and C-reactive protein-triglyceride-glucose index (CTI). The triglyceride glucose-Chinese visceral adiposity index (TyG-CVAI) achieved the highest area under the curve (AUC) for CMM prediction (0.679), and compared with the fully adjusted model (Model 4), all indices provided significant incremental predictive values.

**Conclusion:**

Nine TyG-related indices, particularly TyG-CVAI, are strong independent predictors of future CMM in patients with CKM syndrome stage 0–3. These findings underscore the utility of TyG-related indices, particularly TyG-CVAI, in identifying high-risk individuals, thereby informing strategies for the early detection and prevention of CKM syndrome.

**Graphical abstract:**

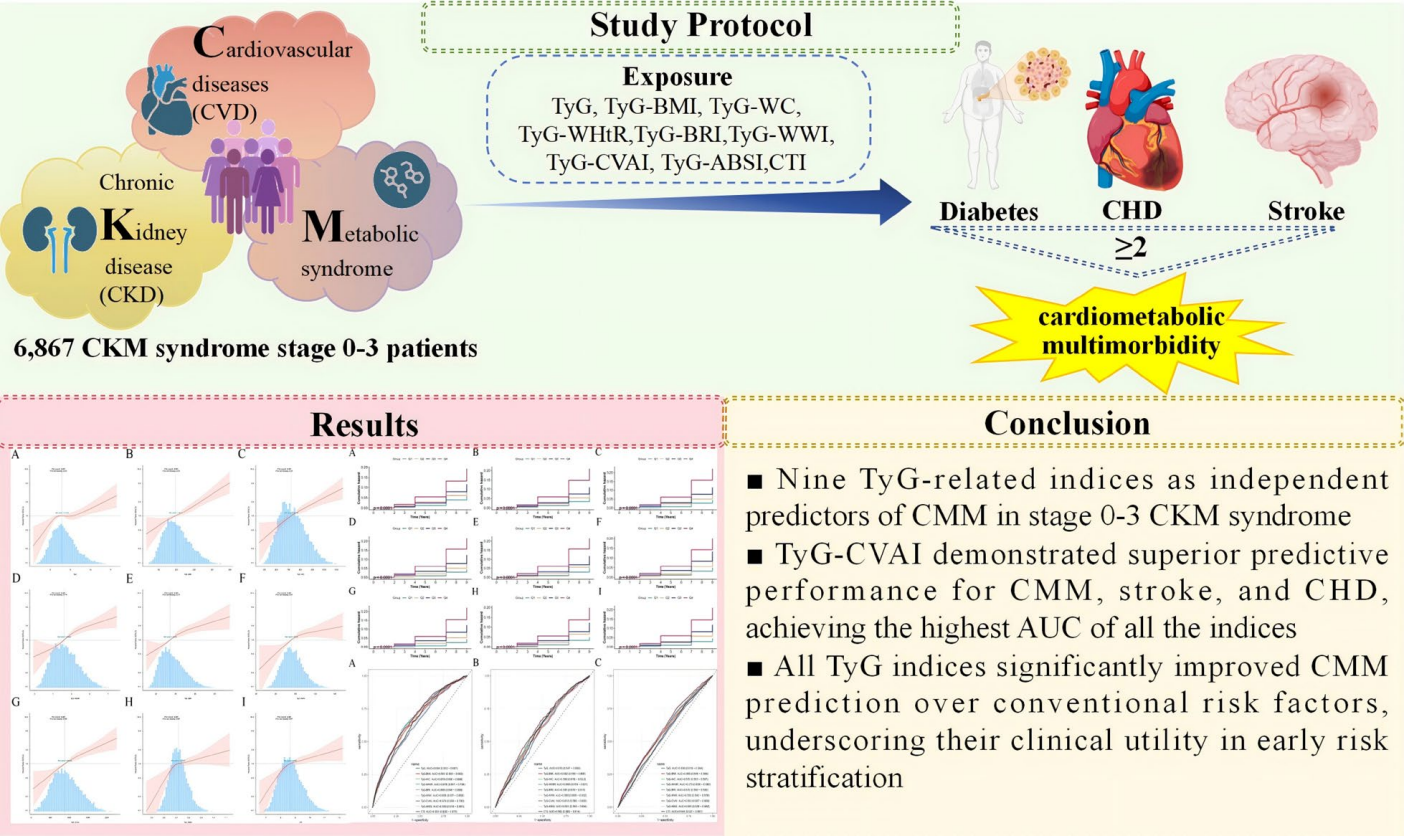

**Supplementary Information:**

The online version contains supplementary material available at 10.1186/s12933-026-03077-4.

## Research insights

**What is currently known about this topic?**

- TyG-related indices are established surrogates for insulin resistance.
- TyG-related indices are linked to individual cardiometabolic diseases.

**What is the key research question?**

- Can nine TyG-related indices predict CMM risk in patients with stage 0-3 CKM syndrome, and which performs best?

**What is new?**

- First study to establish nine TyG-related indices as independent predictors of CMM in stage 0-3 CKM syndrome.
- TyG-CVAI demonstrated superior predictive performance for CMM, stroke, and CHD, achieving the highest AUC of all the indices.
- All TyG indices significantly improved CMM prediction over conventional risk factors.

**How might this study influence clinical practice?**

- Routinely using TyG-CVAI could enhance early risk stratification, enabling targeted prevention to halt CMM progression in CKM patients.

## Introduction

CKM syndrome is a systemic condition that was first defined in the American Heart Association (AHA) October 2023 Presidential Advisory Report [1]. The syndrome describes the pathophysiological interplay among metabolic risk factors, chronic kidney disease (CKD), and the cardiovascular system, which substantially increases the risk of cardiovascular morbidity and mortality. Central to its conceptualization is the emphasis on the disease continuum, which progresses from metabolic risk factors such as obesity and insulin resistance to subclinical cardiovascular damage and, ultimately, to significant cardiorenal events [2]. The rising prevalence of CKM syndrome, which is associated with a significant societal burden, poses systemic challenges to global public health, economic development, and social equity [3–5]. A key manifestation of CKM syndrome is CMM, defined as the presence of at least two major cardiometabolic diseases (e.g., diabetes, coronary heart disease (CHD), or stroke) [6], which is associated with unhealthy lifestyles, cognitive decline, and elevated mortality risk in middle-aged and older Chinese adults [7–9]. Therefore, there is an urgent clinical need to identify reliable and accessible biomarkers in patients with CKM syndrome for the early detection of those at high risk of developing CMM, which is essential for implementing preventive strategies to halt or slow disease progression [10, 11].

The TyG index, a surrogate marker of insulin resistance based on fasting triglyceride and glucose levels, has emerged as a reliable predictor of diabetes, cardiovascular disease (CVD), and CKD [12–14]. Based on the TyG index, several novel composite indices integrating anthropometric measurements or inflammatory markers have been developed to improve risk stratification [15–22]. These indices include the TyG index combined with the body mass index (TyG-BMI), waist circumference (TyG-WC), waist-to-height ratio (TyG-WHtR), body roundness index (TyG-BRI), weight-adjusted waist circumference index (TyG-WWI), Chinese visceral fat index, a body shape index, and C-reactive protein-triglyceride-glucose index. Collectively, these factors capture a spectrum of pathophysiological features, including dyslipidemia, hyperglycemia, visceral adiposity, body shape, and systemic inflammation, thereby enabling a more comprehensive risk assessment.

Although TyG-related indices have been increasingly associated with single cardiometabolic diseases and mortality, their ability to predict CMM, especially in early to mid-stage CKM syndrome (stages 0–3)—is not well established [23–25]. Moreover, the specific utility and comparative performance of all nine indices in this high-risk context remain unknown. Clarifying their predictive value is imperative, since early stage interventions offer the greatest potential to halt progression to multimorbidity.

Critically, while prior studies using the CHARLS database have examined insulin resistance surrogates and CMM in general or metabolically high-risk populations [23, 25], our study is the first to specifically investigate this relationship within the newly defined CKM syndrome framework, focusing on CKM syndrome in stages 0–3. This stage-specific focus targets the early to mid-spectrum of the syndrome continuum, where interventions hold the greatest potential to halt the progression to multimorbidity. Furthermore, we conduct a comprehensive head-to-head comparison of nine TyG-related indices to determine which composite marker—integrating distinct pathways of dyslipidemia, visceral adiposity, body shape, and inflammation—provides the strongest predictive value for CMM in this defined population, thereby addressing a key gap for precise risk stratification.

As the core pathophysiological mechanism of metabolic syndrome, insulin resistance, a state of impaired insulin sensitivity, predisposes individuals to stroke and cardiovascular disease by contributing to increased vascular stiffness [26, 27]. Although the euglycemic hyperinsulinemic clamp technique is considered the gold standard for assessing insulin resistance, its cumbersome operation and high labor costs make it difficult to use in clinical settings [28]. Therefore, identifying alternative indicators that can effectively identify insulin resistance and related cardiovascular risks in large populations and routine clinical work has become an important research direction.

To this end, this study utilized data from a prospective cohort of middle-aged and elderly Chinese adults to: first, investigate the prospective associations between nine TyG-related indices and incident CMM in individuals with CKM syndrome stage 0–3; second, evaluate and compare the predictive performance and incremental value of these indices for CMM, stroke, and CHD; and finally, examine the robustness of these associations through age-stratified and sensitivity analyses.

## Methods

### Study population and design

This prospective cohort analysis was based on data from the China Health and Retirement Longitudinal Study (CHARLS). The CHARLS is a large-scale, nationally representative, interdisciplinary longitudinal survey that aims to collect high-quality data from Chinese households and individuals aged 45 years or older. The baseline survey was initiated in 2011, recruiting participants from 150 county-level units and 450 communities across 28 provinces, with an initial response rate of 80.5% [29]. Participants were followed up with biennial assessments from 2013 to 2020 to monitor dynamic changes within the cohort.

The CHARLS study adhered to the principles of the Declaration of Helsinki and was approved by the Peking University Institutional Review Board (IRB 00001052–11015). All participants provided written informed consent before participating in the CHARLS.

**Fig. 1:**
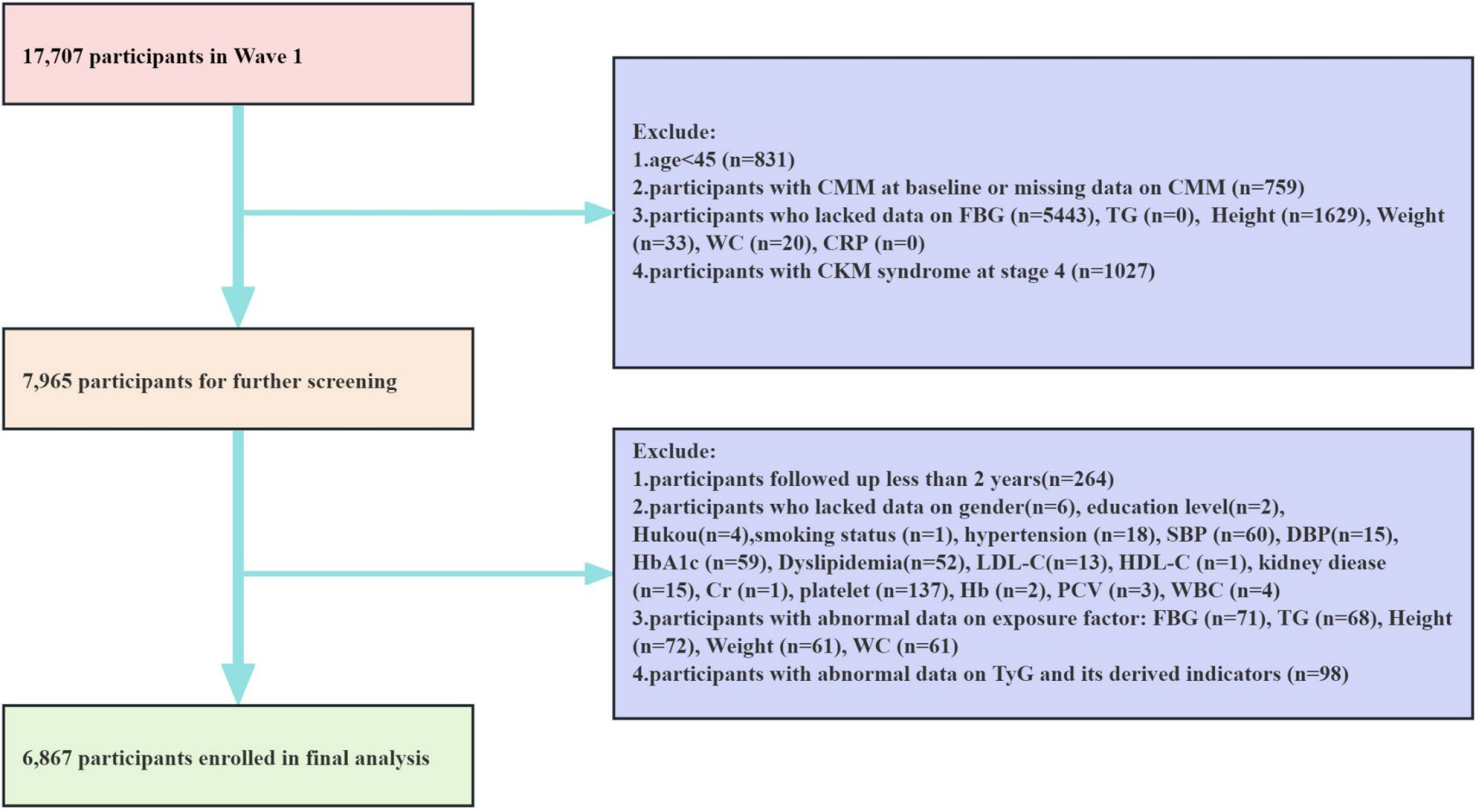
Flowchart of population selection for the CHARLS from 2011 to 2020. CMM, cardiometabolic multimorbidity; FBG, fasting blood glucose; TG, triglyceride; WC, waist circumference; CRP, C-reactive protein; CKM syndrome, cardiovascular kidney-metabolic syndrome; SBP, systolic blood pressure; DBP, diastolic blood pressure; LDL-C, low-density lipoprotein cholesterol; HDL-C, high-density lipoprotein cholesterol; Cr, creatinine; Hb, hemoglobin; PCV, packed cell volume; WBC, white blood cell count; TyG, triglyceride-glucose index

This study initially included 17,707 participants enrolled in the 2011 baseline survey. The following exclusion criteria were applied sequentially: (1) age < 45 years; (2) presence of CMM at baseline or missing CMM data; (3) missing data on fasting blood glucose (FBG), triglyceride (TG), height, weight, waist circumference (WC), C-reactive protein (CRP); (4) participants with CKM syndrome at stage 4. After these exclusions, 7965 participants were eligible for further screening. Subsequently, additional participants were excluded based on the following criteria: (1) follow-up duration of less than 2 years; (2) missing data on demographic, lifestyle, or clinical covariates (including gender, education, Hukou, smoking status, hypertension, systolic blood pressure (SBP) and diastolic blood pressure (DBP), hemoglobin A1c (HbA1c), dyslipidemia, low-density lipoprotein cholesterol (LDL-C), high-density lipoprotein cholesterol (HDL-C), kidney disease, creatinine (Cr), platelet count, hemoglobin (Hb), mean corpuscular volume (MCV), packed cell volume (PCV), and white blood cell count (WBC); (3) abnormal values in exposure variables; (4) abnormal data on TyG and its derived indicators. This exclusion is critical because stage 4 is defined by the presence of established clinical cardiovascular disease (CVD), which is a component of the primary outcome (CMM) in this study. Including these individuals would confound the assessment of incident CMM by mixing participants who already had a component of the outcome with those who did not, thereby compromising the prospective analysis of the risk predictors. After this rigorous screening process, 6867 participants were included in the final analysis. The participant selection flowchart is shown in Fig. 1.

### Assessment of TyG-related indices

In this study, we calculated nine TyG-related indices using the following formula [24, 30–32]:

TyG = ln [TG (mg/dL) × FBG (mg/dL)/2]

TyG − BMI = TyG × BMI

TyG − WC = TyG × WC

TyG − WHtR = TyG × WHtR

TyG − BRI = TyG × BRI

TyG − WWI = TyG × WWI

TyG − CVAI = TyG × CVAI

TyG − ABSI = TyG × ABSI

CTI = 0.412 * Ln (CRP [mg/L]) + TyG

BMI = weight (kg) / height^2^ (m^2^)

WHtR = WC (cm) / height (cm).

BRI = 364.2 − 365.5 ×√1 − (WC (cm) /2π)^2^/ (height (cm) /2)^2^.

WWI = WC (cm) /√weight (kg).

CVAI = − 267.93 + 0.68 × age (years) + 0.03 × BMI (kg/m^2^) + 4.00 × WC (cm) + 22.00 × log_10_ (TG) (mmol/L) − 16.32 × HDL − C (mmol/L) in male.

CVAI = − 187.32 + 1.71 × age (years) + 4.23 × BMI (kg/m^2^) + 1.12 × WC (cm) + 39.76 × log_10_ (TG) (mmol/L) − 11.66 × HDL − C (mmol/L) in female.

ABSI = WC(m) / (BMI (kg/m^2^)^2/3^× height (m)^1/2^).

### Outcome definition and assessment

The definition of CMM was consistent with that used in prior studies [33–35]. The primary endpoint of this study was the occurrence of CMM events, characterized by the coexistence of at least two cardiometabolic diseases (CMDs), including stroke, CHD, and diabetes mellitus. CHD was determined by asking, “Have you been diagnosed with heart disease, coronary artery disease, angina, congestive heart failure, or other heart problems?“. Similarly, stroke was determined by asking, “Have you been diagnosed with stroke?” [36]. Although self-reporting of cardiovascular events has recognized limitations, this method is commonly employed in prior studies to assess population-level risk associations [9, 37, 38]. Participants who reported a history of diabetes or were treated for diabetes and had a baseline FBG ≥ 7.0 mmol/L (126 mg/dL) or HbA1c ≥ 6.5% were defined as having diabetes [39].

### Definition of CKM syndrome stage 0–3

CKM syndrome stage 0–3 were classified according to the AHA Presidential Advisory Statement on CKM syndrome [1]. CKM syndrome stages are as follows: stage 0 indicates the absence of CKM risk factors; stage 1 is characterized by excessive or dysfunctional obesity; stage 2 includes metabolic risk factors or CKD; and Stage 3 includes subclinical CVD [1, 2], as described in Table S1. For this classification, very high-risk CKD (G4 or G5 CKD) and a 10-year high risk of CVD predicted by the Framingham risk score were used as the risk equivalents of subclinical CVD [36]. The estimated glomerular filtration rate (eGFR) was calculated using the Chinese Modification of Diet in Renal Disease (C-MDRD) formula [40], and CKD stage was determined according to the Kidney Disease Improving Global Outcomes (KDIGO) organization [1].

### Covariates

In this study, the following five indicators constituted covariates: (1) demographic data: age, gender, marital status (categorized as married or others), education level (categorized as secondary school or below, high school, college, or above), and Hu kou (categorized as agricultural or others); (2) lifestyle data: smoking status (categorized as yes or no), drinking status (categorized as yes or no) (3) disease history (yes or no): hypertension, dyslipidemia, kidney disease, stages of CKM syndrome; (4) laboratory test data: HDL-C, LDL-C, eGFR, uric acid (UA), Cr, blood urea nitrogen (BUN), Hb, MCV, PCV, Platelet count, WBC; (5) physical measurements: SBP, DBP.

Participants who reported a history of hypertension, received treatment for hypertension, or had a baseline SBP ≥ 140 mmHg or DBP ≥ 90 mmHg were defined as having hypertension [41]. Participants who reported a history of dyslipidemia, received treatment for dyslipidemia, or had a triglyceride (TC) ≥ 240 mg/dL, TG ≥ 150 mg/dL, LDL–C ≥ 160 mg/dL, or HDL–C < 40 mg/dL at baseline were defined as having dyslipidemia [42]. Kidney disease was defined as an eGFR < 60 ml/min/1.73 m² and/or a self-reported medical history [43]. Blood samples were collected by on-site medical professionals and immediately sent to a laboratory at a local partner hospital for analysis. The laboratory used standardized testing methods and equipment to ensure the accuracy and reliability of the data. Vital sign measurements primarily included the pulse rate, which was measured using an OMRON HEM7200 electronic blood pressure monitor. During the measurement, the participants were required to remain quiet. The pulse rate was measured three times, with 45-second intervals between each measurement. The average of the three measurements was used as the final pulse rate. Anthropometric measurements, including height, weight, and waist circumference, were obtained by trained interviewers using a standardized protocol. Height was measured using a Seca™ 213 height meter, accurate to 0.1 cm. Weight was measured using an Omron™ HN-286 electronic scale, accurate to 0.1 kg. When measuring waist circumference, the subject should face the measuring tape and wrap it horizontally around the lower edge of the navel, with an error of less than 0.5 cm. All measurements were performed while the participants were wearing light clothing and were unshoes [29].

### Statistical analysis

Table S2 lists the missing data for the participants. Multiple imputation was applied to handle missing values, presuming that the data were missing at random. Multicollinearity among all covariates was assessed using the generalized variance inflation factor (GVIF). A threshold of GVIF^(1/(2×DF)) ≥ 2 was adopted to identify variables with significant collinearity, where DF represents the degrees of freedom. To avoid multicollinearity in comparative effect estimation, we fitted separate cox models for each index; the results are shown in Tables S3.1–S3.9. Values exceeding the 0.5th and 99.5th percentiles were replaced with the corresponding thresholds, preserving the central 99th percentile of the distribution.

Normally distributed continuous variables are described as mean ± standard deviation (SD) and were compared using the Student’s t-test. Non-normally distributed continuous variables are described as medians with interquartile ranges and were compared using the Mann-Whitney U test. The normality of the distribution was assessed using the Shapiro-Wilk test and visual inspection of histograms. The participants were stratified according to CKM stage (0, 1, 2, or 3). Cox proportional hazards regression models were employed to estimate the associations between the nine TyG-related indices and incident CMM, with results expressed as hazard ratios (HRs) and 95% confidence intervals (CIs). The *P* value for trend was calculated by treating the quartiles of each TyG-related index as ordinal variables (scores 1–4) in the Cox model. A significant P for trend (< 0.05) indicates a statistically significant linear increase (or decrease) in the hazard of the outcome across ascending quartiles of the exposure index. Four models were constructed: Model 1 adjusted for age and gender; Model 2 adjusted for age, gender, marital status, education level, Hukou, smoking and drinking status; Model 3 incorporated adjustments for hypertension, diabetes, dyslipidemia, and kidney disease, beyond the covariates included in Model 2; and Model 4 extended Model 3 by adding physiological measures (SBP and DBP), hematological parameters (HDL-C, LDL-C, eGFR, UA, Cr, BUN, Hb, MCV, PCV, platelet count, and WBC) and stages of CKM syndrome. The proportional hazards assumption for all Cox models was tested using the Schoenfeld residuals. Global tests for the models and tests for individual variables indicated no significant violations of the proportional hazards assumption (all *p*-values > 0.05), supporting the validity of the model application (Tables S13–S15). Furthermore, RCS regression was used to examine the dose-response relationships between the nine TyG-derived indices and CMM incidence in participants with CKM syndrome stage 0–3. In the RCS analysis, four knots were placed at the 5th, 35th, 65th, and 95th percentiles of the distribution for each TyG-related index. The reference value for calculating the hazard ratios (HRs) was set at the median value of each index. Each of the nine TyG-related indices was divided into quartiles (Q1-Q4) based on their distribution in the entire study population at baseline, with Q1 representing the lowest 25% and Q4 representing the highest 25% of the index values. K-M survival curves were generated to compare the cumulative event-free survival across the quartile groups.

The predictive performances of the nine TyG-related indices for CMM, stroke, and CHD in CKM syndrome stages 0–3 were evaluated using ROC curves. The DeLong test was used to compare the areas under the ROC curves for the different TyG-related indices. Their incremental value over the basic model was further assessed using NRI and IDI analyses. To assess the potential effect modification by age, we conducted stratified analyses using the fully adjusted model, categorizing participants into age groups (< 60, 60 –70, and ≥ 70 years). Furthermore, sensitivity analyses were performed to test the robustness of the findings: (1) excluding participants with diabetes at baseline; (2) excluding participants in CKM stage 0 at baseline; (3) utilizing a dataset after multiple imputation. (4) supplementing the primary Cox regression analyses with Fine and Gray subdistribution hazard models for the outcome of incident CMM to account for competing risks of death from non-CMM causes.

All statistical analyses were performed using the R statistical software (Version 4.2.2, http://www.R-project.org, The R Foundation) and the Free Statistics Analysis Platform (Version 2.1.1, Beijing, China, http://www.clinicalscientists.cn/freestatistics). A two-sided *P*-value < 0.05 was considered statistically significant.

## Results

### Baseline characteristics

The baseline data of the participants are shown in Table 1. The final analytic sample comprised 6,867 individuals with CKM syndrome stage 0–3. The study population had a mean age of 59.2 ± 9.0 years and comprised 47.6% of males. The participants were stratified into four groups based on the CKM syndrome stage (0, 1, 2, or 3). Compared with those in CKM syndrome stage 3, participants in stage 0 were younger, had lower rates of smoking and alcohol consumption, and exhibited a lower prevalence of hypertension, diabetes, and dyslipidemia. They also demonstrated more favorable clinical profiles, including lower SBP, DBP, HbA1c, FBG, TG, TC, LDL-C, UA, Cr, and BUN levels and higher eGFR and HDL-C levels. The height, weight, body mass index (BMI), WC, CRP, and WBC levels were also generally lower in the CKM syndrome stage 0 group. To further characterize the study population, the baseline characteristics were summarized according to the incidence of CMM (Table S4).

**Table 1:** Characteristics of 6,867 participants based on CKM syndrome stage 0–3

Characteristics	Total	CKM				*P*
		Stage 0	Stage 1	Stage 2	Stage 3	
n	6867	552	1106	3030	2179	
Age Mean ± SD	59.2 ± 9.0	56.6 ± 7.5	56.1 ± 7.8	56.7 ± 8.0	65.1 ± 8.4	< 0.001
Gender (%)						< 0.001
Male	3270 (47.6)	303 (54.9)	432 (39.1)	779 (25.7)	1756 (80.6)	
Female	3597 (52.4)	249 (45.1)	674 (60.9)	2251 (74.3)	423 (19.4)	
Marital status (%)						< 0.001
Married	6091 (88.7)	501 (90.8)	1009 (91.2)	2719 (89.7)	1862 (85.5)	
Others	776 (11.3)	51 (9.2)	97 (8.8)	311 (10.3)	317 (14.5)	
Education level (%)						0.141
Secondary school or below	6212 (90.5)	488 (88.4)	996 (90.1)	2738 (90.4)	1990 (91.3)	
High school	575 (8.4)	60 (10.9)	95 (8.6)	260 (8.6)	160 (7.3)	
College or above	80 (1.2)	4 (0.7)	15 (1.4)	32 (1.1)	29 (1.3)	
Hukou (%)						< 0.001
Agriculture	5853 (85.2)	503 (91.1)	965 (87.3)	2590 (85.5)	1795 (82.4)	
Others	1014 (14.8)	49 (8.9)	141 (12.7)	440 (14.5)	384 (17.6)	
Smoking status (%)						< 0.001
No	4145 (60.4)	320 (58)	773 (69.9)	2470 (81.5)	582 (26.7)	
Yes	2722 (39.6)	232 (42)	333 (30.1)	560 (18.5)	1597 (73.3)	
Drinking status (%)						< 0.001
No	4545 (66.2)	333 (60.3)	730 (66)	2317 (76.5)	1165 (53.5)	
Yes	2322 (33.8)	219 (39.7)	376 (34)	713 (23.5)	1014 (46.5)	
Hypertension (%)						< 0.001
No	4309 (62.7)	552 (100)	1106 (100)	1801 (59.4)	850 (39)	
Yes	2558 (37.3)	0 (0)	0 (0)	1229 (40.6)	1329 (61)	
Diabetes (%)						< 0.001
No	6462 (94.1)	552 (100)	1106 (100)	2850 (94.1)	1954 (89.7)	
Yes	405 (5.9)	0 (0)	0 (0)	180 (5.9)	225 (10.3)	
Dyslipidemia (%)						< 0.001
No	3697 (53.8)	552 (100)	1106 (100)	1111 (36.7)	928 (42.6)	
Yes	3170 (46.2)	0 (0)	0 (0)	1919 (63.3)	1251 (57.4)	
Kidney disease (%)						0.366
No	6456 (94.0)	527 (95.5)	1045 (94.5)	2843 (93.8)	2041 (93.7)	
Yes	411 (6.0)	25 (4.5)	61 (5.5)	187 (6.2)	138 (6.3)	
SBP, mmHg	128.8 ± 22.4	112.5 ± 11.4	115.4 ± 10.7	127.6 ± 20.8	141.4 ± 23.8	< 0.001
DBP, mmHg	75.0 ± 12.1	67.1 ± 9.0	68.9 ± 8.5	75.6 ± 11.9	79.2 ± 12.3	< 0.001
FBG, mg/dL	108.3 ± 27.6	90.7 ± 6.8	100.6 ± 10.4	108.5 ± 26.3	116.6 ± 34.6	< 0.001
HbA1c (%)	5.2 ± 0.7	5.0 ± 0.4	5.1 ± 0.4	5.2 ± 0.7	5.4 ± 0.9	< 0.001
HDL-C, mg/dL	51.7 ± 15.0	59.4 ± 12.5	59.4 ± 12.2	49.8 ± 14.4	48.4 ± 15.6	< 0.001
LDL-C, mg/dL	117.2 ± 34.3	105.0 ± 23.0	110.1 ± 23.5	119.6 ± 36.3	120.4 ± 37.0	< 0.001
TC, mg/dL	193.5 ± 37.5	177.9 ± 24.8	183.2 ± 26.6	197.0 ± 39.4	197.6 ± 40.0	< 0.001
TG, mg/dL	126.1 ± 78.7	77.6 ± 24.6	81.3 ± 24.7	140.7 ± 78.0	140.7 ± 92.1	< 0.001
eGFR, mL/min per 1.73 m^2^	108.5 ± 27.7	113.4 ± 25.1	114.3 ± 26.9	110.6 ± 28.4	101.4 ± 26.4	< 0.001
UA, mg/dL	4.4 ± 1.2	4.2 ± 1.1	4.1 ± 1.1	4.3 ± 1.2	4.9 ± 1.3	< 0.001
Cr, mg/dL	0.8 ± 0.2	0.8 ± 0.2	0.7 ± 0.2	0.7 ± 0.2	0.9 ± 0.3	< 0.001
BUN, mg/dL	15.7 ± 4.5	16.0 ± 4.5	15.7 ± 4.3	15.2 ± 4.3	16.5 ± 4.7	< 0.001
Height, m	157.9 ± 8.2	158.2 ± 8.3	157.4 ± 8.3	156.1 ± 7.8	160.5 ± 8.1	< 0.001
Weight, kg	58.2 ± 10.3	50.6 ± 7.2	56.8 ± 9.2	58.6 ± 10.2	60.1 ± 10.7	< 0.001
WC, cm	84.8 ± 9.5	75.3 ± 5.3	83.0 ± 7.9	86.0 ± 9.3	86.5 ± 9.7	< 0.001
BMI, kg/m^2^	23.3 ± 3.4	20.1 ± 1.7	22.9 ± 2.9	24.0 ± 3.4	23.3 ± 3.4	< 0.001
CRP, mg/L	1.0 (0.5, 2.1)	0.7 (0.4, 1.5)	0.8 (0.5, 1.5)	1.0 (0.5, 2.0)	1.3 (0.7, 2.7)	< 0.001
Hb, g/dL	14.4 ± 2.2	14.0 ± 2.2	14.1 ± 2.1	14.1 ± 2.1	14.9 ± 2.2	< 0.001
MCV, fL	90.7 ± 8.5	90.7 ± 9.0	90.6 ± 8.8	89.9 ± 8.3	92.0 ± 8.4	< 0.001
PCV (%)	41.7 ± 6.1	41.0 ± 6.0	40.9 ± 6.2	41.0 ± 5.9	43.1 ± 6.0	< 0.001
Platelet count, 10^9^/L	211.6 ± 76.1	209.6 ± 74.1	211.7 ± 90.6	214.1 ± 70.8	208.7 ± 75.6	0.081
WBC, 10^9^/L	6.3 ± 1.9	6.0 ± 1.9	5.9 ± 1.8	6.2 ± 1.9	6.5 ± 1.9	< 0.001

Data are presented as mean ± SD or n (percentage)

CKM, cardiovascular kidney-metabolic; SBP, systolic blood pressure; DBP, diastolic blood pressure; FBG, fasting blood glucose; HbA1c, hemoglobin A1c; HDL-C, high-density lipoprotein cholesterol; LDL-C, low-density lipoprotein cholesterol; TC, total cholesterol; TG, triglyceride; eGFR, estimated glomerular filtration rate; UA, uric acid; Cr, creatinine; BUN, blood urea nitrogen; WC, waist circumference; BMI, body mass index; CRP, C-reactive protein; Hb, hemoglobin; MCV, mean corpuscular volume; PCV, packed cell volume; WBC, white blood cell count; SD, standard deviation

### Associations between TyG-related indices and CMM in CKM syndrome stage 0–3

Over a median follow-up of 9 years, 652 participants (9.5%) developed CMM. In the fully adjusted Cox proportional hazards model (Model 4), all nine TyG-related indices were significantly associated with CMM in the highest quartile (Table 2). A significant positive trend was observed for all indices (all P for trend < 0.05), indicating a monotonically increasing risk of CMM with ascending quartile levels. In contrast, the associations with stroke and CHD were substantially reduced. For some of the indices examined (e.g., TyG, TyG-WWI, and TyG-ABSI), the hazard ratios approached 1.00, with confidence intervals spanning the null value, indicating statistically non-significant effects (Tables S5 and S6).

**Table 2:** Associations of TyG-related indices with future CMM risk in CKM syndrome stage 0–3

	Case	Crude		Model 1		Model 2		Model 3		Model 4	
		HR (95%CI)	*P*	HR (95%CI)	*P*	HR (95%CI)	*P*	HR (95%CI)	*P*	HR (95%CI)	*P*
TyG											
Q1	83 (4.8)	1(Ref)									
Q2	139 (8.1)	1.68 (1.28–2.21)	< 0.001	1.69 (1.29–2.21)	< 0.001	1.67 (1.28–2.2)	< 0.001	1.55 (1.18–2.04)	0.002	1.44 (1.09–1.9)	0.01
Q3	165 (9.6)	2.03 (1.56–2.65)	< 0.001	1.98 (1.52–2.58)	< 0.001	1.95 (1.49–2.54)	< 0.001	1.57 (1.19–2.06)	0.001	1.34 (1.01–1.78)	0.045
Q4	265 (15.4)	3.4 (2.66–4.35)	< 0.001	3.4 (2.66–4.36)	< 0.001	3.33 (2.6–4.27)	< 0.001	1.88 (1.41–2.51)	< 0.001	1.62 (1.2–2.2)	0.002
Trend test	652 (9.5)	1.47 (1.37–1.58)	< 0.001	1.47 (1.37–1.58)	< 0.001	1.46 (1.36–1.57)	< 0.001	1.19 (1.09–1.31)	< 0.001	1.14 (1.04–1.25)	0.007
TyG-BMI											
Q1	81 (4.7)	1(Ref)									
Q2	120 (7)	1.45 (1.1–1.93)	0.009	1.56 (1.18–2.07)	0.002	1.56 (1.17–2.07)	0.002	1.38 (1.04–1.84)	0.026	1.29 (0.96–1.72)	0.09
Q3	157 (9.1)	1.92 (1.47–2.51)	< 0.001	2.13 (1.62–2.79)	< 0.001	2.1 (1.6–2.76)	< 0.001	1.58 (1.19–2.09)	0.002	1.42 (1.06–1.91)	0.02
Q4	294 (17.1)	3.71 (2.9–4.74)	< 0.001	4.25 (3.31–5.47)	< 0.001	4.19 (3.25–5.4)	< 0.001	2.5 (1.88–3.31)	< 0.001	2.18 (1.61–2.95)	< 0.001
Trend test	652 (9.5)	1.57 (1.45–1.69)	< 0.001	1.63 (1.51–1.76)	< 0.001	1.62 (1.5–1.75)	< 0.001	1.35 (1.24–1.47)	< 0.001	1.3 (1.18–1.43)	< 0.001
TyG-WC											
Q1	63 (3.7)	1(Ref)									
Q2	120 (7)	1.92 (1.42–2.61)	< 0.001	1.93 (1.42–2.62)	< 0.001	1.92 (1.41–2.61)	< 0.001	1.74 (1.28–2.36)	< 0.001	1.62 (1.18–2.22)	0.003
Q3	165 (9.6)	2.67 (2–3.57)	< 0.001	2.68 (2–3.58)	< 0.001	2.63 (1.96–3.52)	< 0.001	2.11 (1.56–2.85)	< 0.001	1.91 (1.39–2.63)	< 0.001
Q4	304 (17.7)	5.19 (3.96–6.81)	< 0.001	5.2 (3.96–6.82)	< 0.001	5.08 (3.86–6.68)	< 0.001	3.11 (2.3–4.22)	< 0.001	2.81 (2.03–3.89)	< 0.001
Trend test	652 (9.5)	1.7 (1.58–1.84)	< 0.001	1.7 (1.58–1.84)	< 0.001	1.69 (1.56–1.82)	< 0.001	1.42 (1.3–1.55)	< 0.001	1.38 (1.25–1.51)	< 0.001
TyG-WHtR											
Q1	63 (3.7)	1(Ref)									
Q2	113 (6.6)	1.81 (1.33–2.47)	< 0.001	1.88 (1.38–2.56)	< 0.001	1.86 (1.37–2.54)	< 0.001	1.69 (1.23–2.3)	0.001	1.57 (1.14–2.17)	0.006
Q3	179 (10.4)	2.85 (2.14–3.8)	< 0.001	2.94 (2.2–3.92)	< 0.001	2.87 (2.15–3.84)	< 0.001	2.24 (1.66–3.03)	< 0.001	2.04 (1.49–2.79)	< 0.001
Q4	297 (17.3)	5.08 (3.87–6.67)	< 0.001	5.23 (3.95–6.92)	< 0.001	5.13 (3.87–6.8)	< 0.001	3.06 (2.25–4.18)	< 0.001	2.74 (1.96–3.82)	< 0.001
Trend test	652 (9.5)	1.7 (1.58–1.84)	< 0.001	1.71 (1.58–1.85)	< 0.001	1.7 (1.57–1.84)	< 0.001	1.41 (1.29–1.55)	< 0.001	1.37 (1.24–1.51)	< 0.001
TyG-BRI											
Q1	72 (4.2)	1(Ref)									
Q2	113 (6.6)	1.56 (1.16–2.09)	0.003	1.61 (1.2–2.17)	0.002	1.6 (1.19–2.15)	0.002	1.41 (1.05–1.91)	0.024	1.33 (0.98–1.8)	0.068
Q3	170 (9.9)	2.34 (1.78–3.08)	< 0.001	2.42 (1.83–3.2)	< 0.001	2.37 (1.79–3.14)	< 0.001	1.79 (1.34–2.38)	< 0.001	1.62 (1.21–2.19)	0.001
Q4	297 (17.3)	4.39 (3.39–5.68)	< 0.001	4.55 (3.48–5.96)	< 0.001	4.49 (3.42–5.88)	< 0.001	2.69 (2.01–3.6)	< 0.001	2.4 (1.76–3.26)	< 0.001
Trend test	652 (9.5)	1.66 (1.53–1.79)	< 0.001	1.67 (1.54–1.81)	< 0.001	1.66 (1.53–1.8)	< 0.001	1.39 (1.27–1.51)	< 0.001	1.34 (1.22–1.47)	< 0.001
TyG-WWI											
Q1	72 (4.2)	1(Ref)									
Q2	117 (6.8)	1.66 (1.23–2.22)	0.001	1.63 (1.21–2.19)	0.001	1.62 (1.21–2.17)	0.001	1.43 (1.06–1.92)	0.019	1.34 (0.99–1.81)	0.059
Q3	190 (11.1)	2.72 (2.07–3.57)	< 0.001	2.68 (2.04–3.53)	< 0.001	2.66 (2.02–3.5)	< 0.001	2.01 (1.51–2.67)	< 0.001	1.78 (1.33–2.38)	< 0.001
Q4	273 (15.9)	4.18 (3.22–5.42)	< 0.001	3.98 (3.03–5.23)	< 0.001	3.94 (3–5.17)	< 0.001	2.18 (1.61–2.94)	< 0.001	1.9 (1.39–2.6)	< 0.001
Trend test	652 (9.5)	1.6 (1.49–1.73)	< 0.001	1.58 (1.46–1.71)	< 0.001	1.57 (1.45–1.7)	< 0.001	1.28 (1.17–1.4)	< 0.001	1.23 (1.12–1.35)	< 0.001
TyG-CVAI											
Q1	62 (3.6)	1(Ref)									
Q2	117 (6.8)	1.89 (1.39–2.57)	< 0.001	1.84 (1.35–2.51)	< 0.001	1.83 (1.34–2.5)	< 0.001	1.65 (1.21–2.25)	0.002	1.56 (1.13–2.16)	0.006
Q3	175 (10.2)	2.89 (2.17–3.87)	< 0.001	2.75 (2.05–3.69)	< 0.001	2.69 (2–3.61)	< 0.001	2.11 (1.56–2.87)	< 0.001	1.98 (1.43–2.75)	< 0.001
Q4	298 (17.4)	5.25 (3.99–6.9)	< 0.001	4.83 (3.65–6.38)	< 0.001	4.71 (3.56–6.24)	< 0.001	2.88 (2.11–3.93)	< 0.001	2.71 (1.92–3.81)	< 0.001
Trend test	652 (9.5)	1.71 (1.59–1.85)	< 0.001	1.66 (1.54–1.8)	< 0.001	1.65 (1.53–1.79)	< 0.001	1.38 (1.27–1.51)	< 0.001	1.36 (1.23–1.51)	< 0.001
TyG-ABSI											
Q1	73 (4.3)	1(Ref)									
Q2	140 (8.2)	1.97 (1.48–2.61)	< 0.001	1.89 (1.42–2.51)	< 0.001	1.89 (1.42–2.5)	< 0.001	1.69 (1.27–2.25)	< 0.001	1.54 (1.16–2.06)	0.003
Q3	172 (10)	2.48 (1.88–3.26)	< 0.001	2.32 (1.76–3.05)	< 0.001	2.29 (1.74–3.02)	< 0.001	1.76 (1.33–2.34)	< 0.001	1.56 (1.17–2.09)	0.002
Q4	267 (15.6)	4.09 (3.16–5.3)	< 0.001	3.66 (2.81–4.77)	< 0.001	3.63 (2.79–4.74)	< 0.001	2.11 (1.58–2.83)	< 0.001	1.86 (1.37–2.51)	< 0.001
Trend test	652 (9.5)	1.54 (1.43–1.66)	< 0.001	1.49 (1.38–1.6)	< 0.001	1.48 (1.37–1.6)	< 0.001	1.23 (1.13–1.33)	< 0.001	1.18 (1.08–1.29)	< 0.001
CTI											
Q1	68 (4)	1(Ref)									
Q2	135 (7.9)	2.06 (1.54–2.75)	< 0.001	1.98 (1.48–2.65)	< 0.001	1.96 (1.47–2.63)	< 0.001	1.86 (1.38–2.49)	< 0.001	1.75 (1.3–2.35)	< 0.001
Q3	167 (9.7)	2.61 (1.97–3.46)	< 0.001	2.49 (1.88–3.3)	< 0.001	2.46 (1.85–3.26)	< 0.001	1.99 (1.49–2.67)	< 0.001	1.77 (1.31–2.4)	< 0.001
Q4	282 (16.4)	4.69 (3.6–6.12)	< 0.001	4.45 (3.41–5.8)	< 0.001	4.35 (3.34–5.68)	< 0.001	2.71 (2.01–3.64)	< 0.001	2.39 (1.74–3.28)	< 0.001
Trend test	652 (9.5)	1.61 (1.5–1.74)	< 0.001	1.59 (1.47–1.71)	< 0.001	1.58 (1.46–1.7)	< 0.001	1.33 (1.22–1.45)	< 0.001	1.28 (1.16–1.4)	< 0.001

CMM, cardiometabolic multimorbidity; CKM syndrome, cardiovascular kidney-metabolic syndrome; TyG, triglyceride-glucose index; TyG-BMI, triglyceride glucose-body mass index; TyG-WC, triglyceride glucose-waist circumference index; TyG-WHtR, triglyceride glucose-waist height ratio index; TyG-BRI, triglyceride glucose-body roundness index; TyG-WWI, triglyceride glucose-weight-adjusted waist index; TyG-CVAI, triglyceride glucose-Chinese visceral adiposity index; TyG-ABSI, triglyceride glucose -a body shape index; CTI, C-reactive protein-triglyceride-glucose index; SBP, systolic blood pressure; DBP, diastolic blood pressure; HDL-C, high-density lipoprotein cholesterol; LDL-C, low-density lipoprotein cholesterol; eGFR, estimated glomerular filtration rate; UA, uric acid; Cr, creatinine; BUN, blood urea nitrogen; Hb, hemoglobin; MCV, mean corpuscular volume; PCV, packed cell volume; WBC, white blood cell count

Model 1 adjusted for age, gender

Model 2 adjusted for Model 1 + marital status, education level, Hu kou, smoking status, drinking status

Model 3 adjusted for Model 2 + hypertension, diabetes, dyslipidemia, kidney disease

Model 4 adjusted for Model 3 + SBP, DBP, HDL-C, LDL-C, eGFR, UA, Cr, BUN, Hb, MCV, PCV, platelet count, WBC, stages of CKM syndrome

The hazard ratios (HRs) presented in the table were derived from cox proportional hazards regression models

**Fig. 2:**
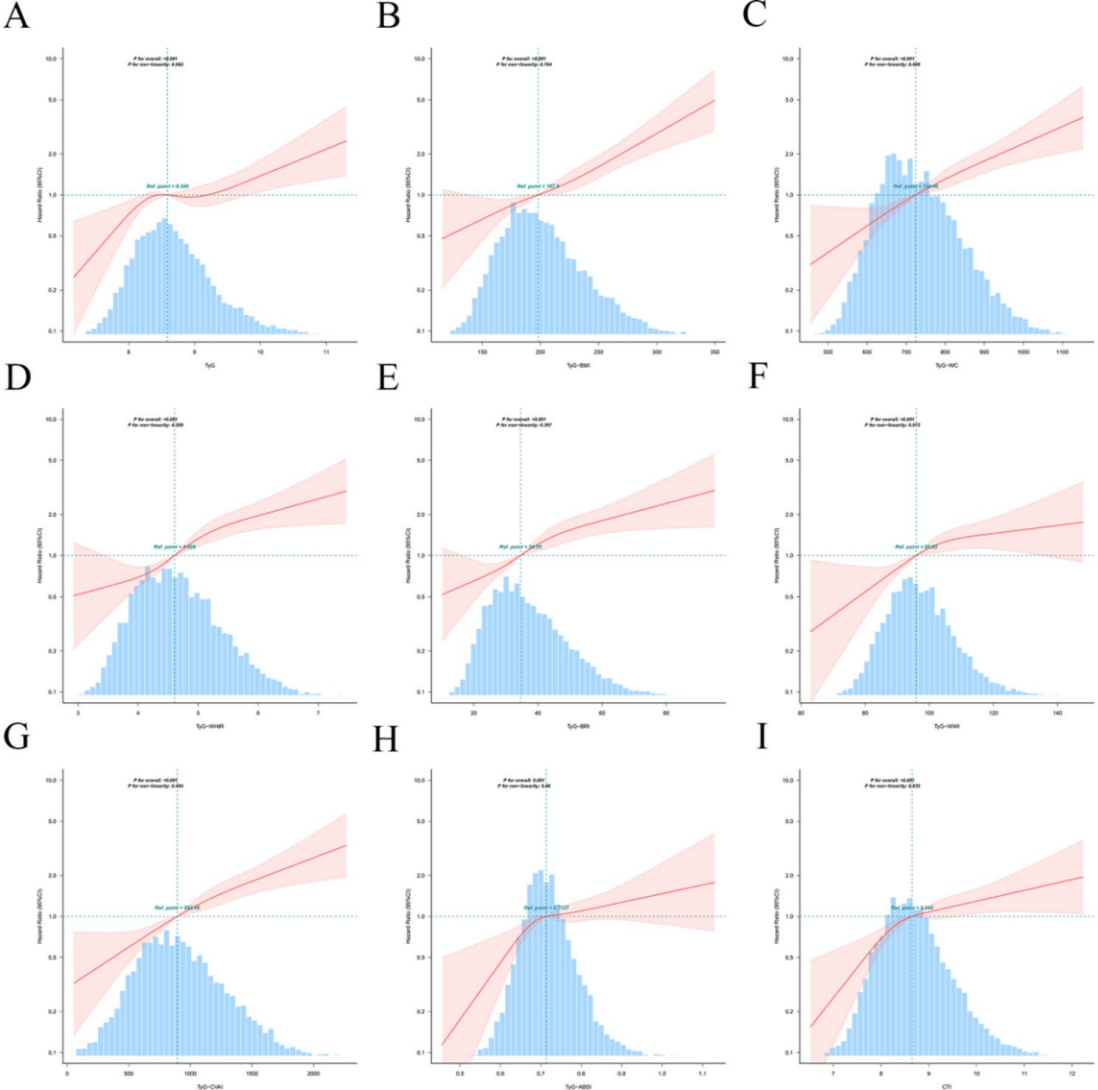
Dose-response relationships between TyG-related indices and CMM risk. Solid lines indicate HRs and shadow shapes indicate 95% CI. The y-axis represents the adjusted HR for the CMM. The reference value (HR = 1) was set at the median of each index. **A** TyG, triglyceride-glucose index; **B** TyG-BMI, triglyceride glucose-body mass index; **C** TyG-WC, triglyceride glucose-waist circumference; **D** TyG-WHtR, triglyceride glucose-waist height ratio index; **E** TyG-BRI, triglyceride glucose-body roundness index; **F** TyG-WWI, triglyceride glucose-weight-adjusted waist index; **G** TyG-CVAI, triglyceride glucose-Chinese visceral adiposity index; (H) TyG-ABSI, triglyceride glucose-a body shape index; **I** CTI, C-reactive protein-triglyceride-glucose index; HR, hazard ratio; CI, confidence interval; CMM, cardiometabolic multimorbidity

Restricted cubic spline analyses revealed distinct dose-response relationships between TyG-related indices and specific cardiometabolic outcomes. For CMM (Fig. 2), TyG, TyG-BMI, TyG-WC, TyG-WHtR, TyG-BRI, TyG-WWI, and TyG-CVAI exhibited positive linear relationships (all P-overall ≤ 0.05; P-nonlinearity ≥ 0.05), whereas TyG-ABSI and CTI showed positive nonlinear associations (both P-overall ≤ 0.05; P-nonlinearity ≤ 0.05). A similar positive association was observed in patients with stroke (Fig. S1). Among them, TyG-BMI, TyG-WC, TyG-WHtR, TyG-BRI, TyG-WWI, TyG-CVAI, and TyG-ABSI displayed linearity (P-nonlinearity ≥ 0.05), while TyG and CTI were nonlinear (P-nonlinearity ≤ 0.05). In contrast, for CHD (Fig. S2), no significant positive associations were found for TyG, TyG-WWI, TyG-ABSI, or CTI (P-overall ≥ 0.05). The remaining five indices (TyG-BMI, TyG-WC, TyG-WHtR, TyG-BRI, and TyG-CVAI) demonstrated positive nonlinear dose-response relationships with CHD (P-overall ≤ 0.05; P-nonlinearity ≤ 0.05).

**Fig. 3:**
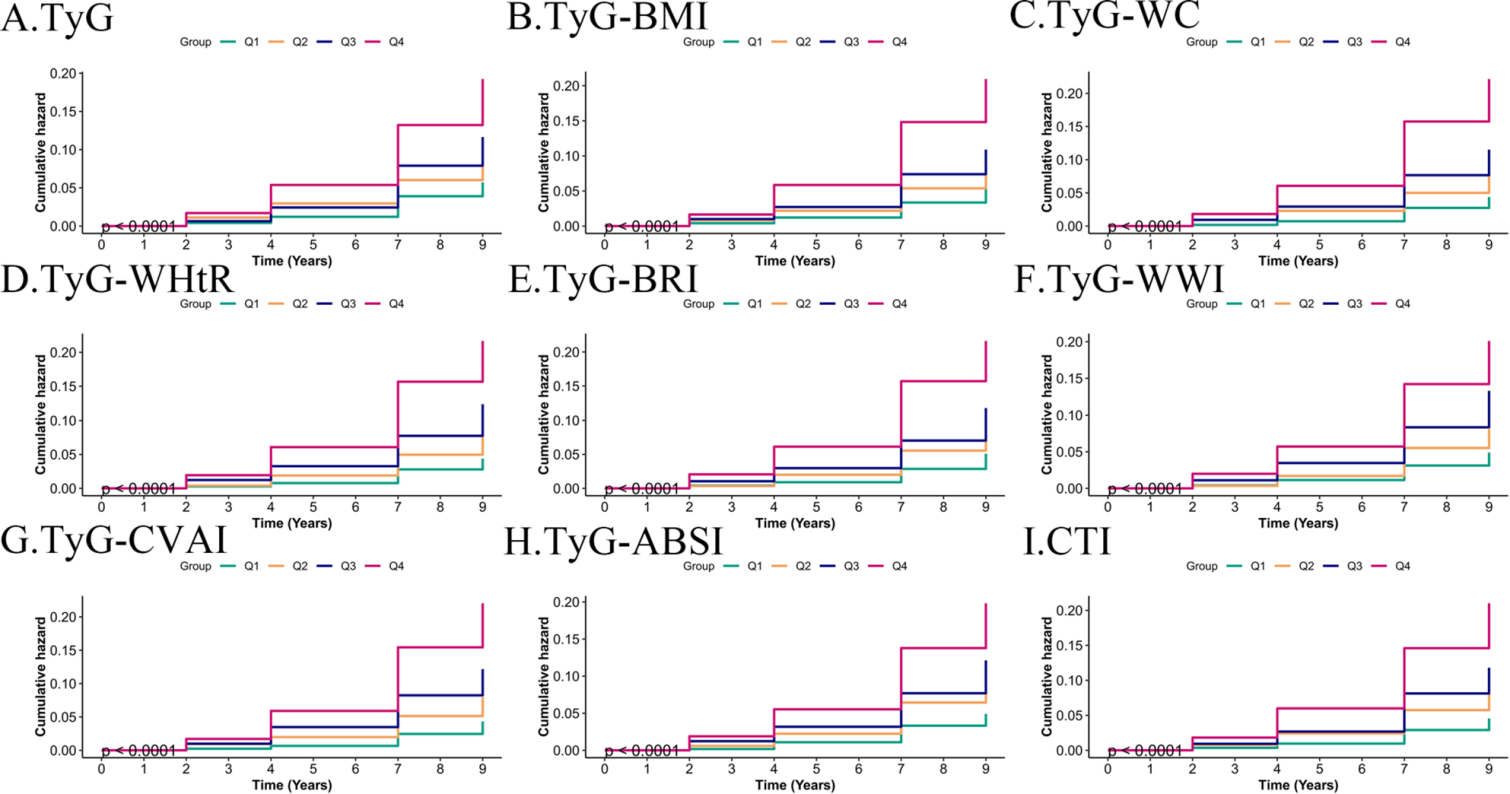
Kaplan–Meier curves for cumulative incidence of CMM by quartiles of TyG-related indices. Participants were stratified into quartiles (Q1-Q4) based on their baseline levels of each index. The cumulative incidence of CMM over the follow-up period is shown. Q1 (lowest quartile) was the reference group. Log-rank tests were performed to compare survival distributions across quartiles, with all *p*-values < 0.001, indicating a significantly higher CMM incidence in the highest quartile (Q4) for all nine indices. **A** TyG, triglyceride-glucose index; **B** TyG-BMI, triglyceride glucose-body mass index; **C** TyG-WC, triglyceride glucose-waist circumference; **D** TyG-WHtR, triglyceride glucose-waist height ratio index; **E** TyG-BRI, triglyceride glucose-body roundness index; **F** TyG-WWI, triglyceride glucose-weight-adjusted waist index; **G** TyG-CVAI, triglyceride glucose-Chinese visceral adiposity index; **H** TyG-ABSI, triglyceride glucose-a body shape index; **I** CTI, C-reactive protein-triglyceride-glucose index; CMM, cardiometabolic multimorbidity

Kaplan-Meier survival analysis demonstrated a significantly higher incidence of CMM among patients in the highest quartile (Q4) of TyG-related indices than among those in the lower quartiles (Q1–Q3) (log-rank test, all *p* < 0.001; Fig. 3). Consistent patterns were observed for stroke and CHD (Figs. S3–S4), reinforcing the association between elevated TyG-related indices and increased cardiometabolic risk. These findings indicate that all nine TyG-related markers show significant and independent associations with CMM development in individuals with CKM stage 0–3. However, the strength and shape of these associations varied according to the index composition and endpoint, highlighting the disease-specific risk patterns.

### Predictive performance of TyG-related indices for CMM in CKM syndrome stage 0–3

**Fig. 4:**
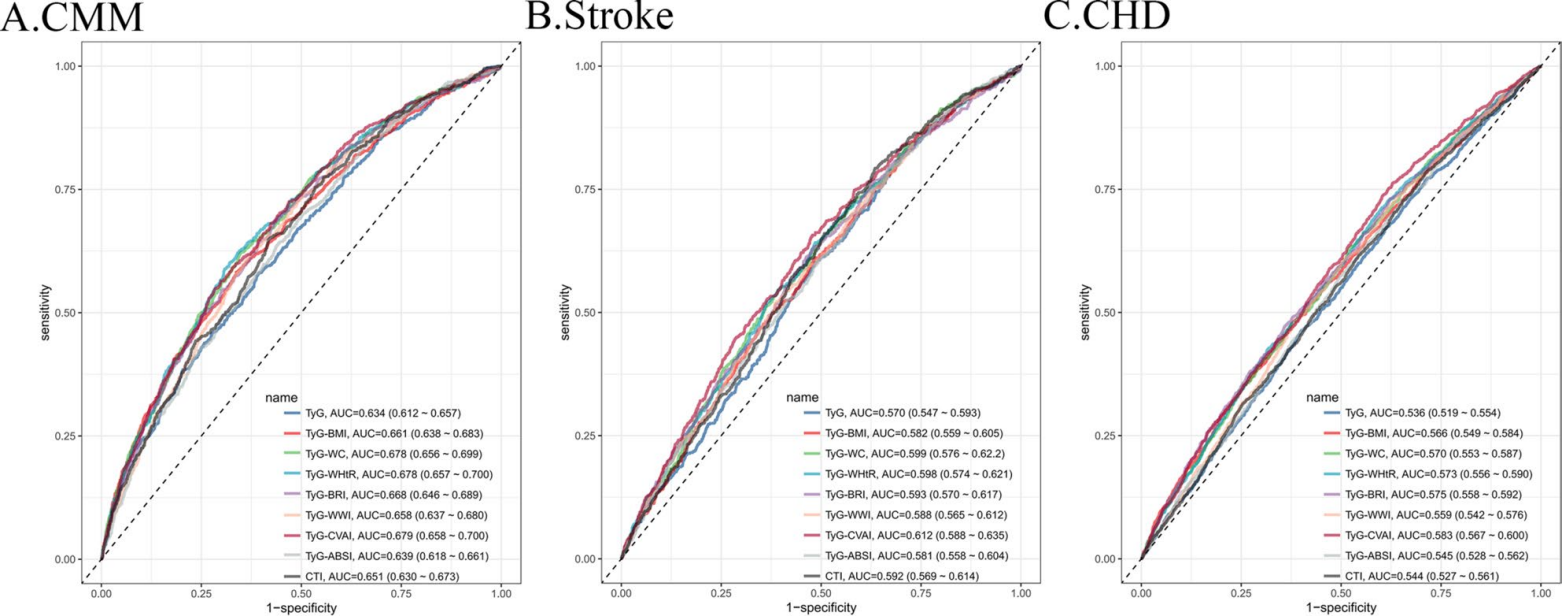
Predictive performance of TyG-related indices for CMM (**A**), stroke (**B**), and CHD (**C**). The area under the curve (AUC) with 95% CI is provided in the legend for each index. TyG, triglyceride-glucose index; TyG-BMI, triglyceride glucose-body mass index; TyG-WC, triglyceride glucose-waist circumference; TyG-WHtR, triglyceride glucose-waist height ratio index; TyG-BRI, triglyceride glucose-body roundness index; TyG-WWI, triglyceride glucose-weight-adjusted waist index; TyG-CVAI, triglyceride glucose-Chinese visceral adiposity index; TyG-ABSI, triglyceride glucose-a body shape index; CTI, C-reactive protein-triglyceride-glucose index; AUC, area under curve; CMM, cardiometabolic multimorbidity; CHD, coronary heart disease

ROC curves demonstrated that TyG-CVAI exhibited the strongest predictive ability for CMM, stroke, and CHD among individuals with CKM syndrome stage 0–3, with AUC values of 0.679 (95% CI 0.658–0.700), 0.612 (0.588–0.635), and 0.583 (0.567–0.600), respectively (Fig. 4). Pairwise comparisons using the DeLong test revealed that the AUC value of TyG-CVAI was higher than that of most TyG-related indices (*p* < 0.05). Although the AUC value of TyG-CVAI was also higher than that of TyG-WC and TyG-WHtR, these differences were not statistically significant (*p* > 0.05). The detailed results of all pairwise comparisons are provided in Fig. S5. The remaining TyG-related indices also showed modest to good predictive performance for the three outcomes.

Based on the fully adjusted model (Model 4), we assessed the incremental predictive value of the nine TyG-related indices for CMM, stroke, and CHD in CKM syndrome stage 0–3 using NRI and IDI analyses. For the CMM, all nine indices demonstrated a statistically significant improvement in predictive performance (Table 3). The optimal cut-off points, sensitivity, specificity, positive predictive value (PPV), and negative predictive value (NPV) of each TyG-related index for predicting CMM, stroke, and CHD are detailed in Table S12. Regarding stroke prediction, five indices (TyG-BMI, TyG-WC, TyG-WHtR, TyG-BRI, and TyG-CVAI) showed significant incremental value, whereas the remaining four did not reach statistical significance (Table S7). Similarly, for CHD, four indices (TyG-BMI, TyG-WC, TyG-BRI, and TyG-CVAI) provided a significant improvement in predictive performance, whereas the other five indices showed no statistically significant enhancement (Table S8).

**Table 3:** Incremental predictive value of TyG-related indices for CMM over the basic model

Model	NRI (95%CI)	*P*	IDI (95%CI)	*P*
Basic model	Ref			
+TyG	0.1244 (0.0440–0.2047)	0.002	0.0026 (0.0007–0.0046)	0.008
+TyG-BMI	0.2268 (0.1465–0.3072)	< 0.001	0.0113 (0.0074–0.0152)	< 0.001
+TyG-WC	0.2551 (0.1751–0.3352)	< 0.001	0.0109 (0.0071–0.0146)	< 0.001
+TyG-WHtR	0.2523 (0.1723–0.3322)	< 0.001	0.0091 (0.0057–0.0125)	< 0.001
+TyG-BRI	0.2144 (0.1340–0.2948)	< 0.001	0.0083 (0.0050–0.0116)	< 0.001
+TyG-WWI	0.1512 (0.0707–0.2316)	< 0.001	0.0035 (0.0013–0.0057)	0.002
+TyG-CVAI	0.2006 (0.1202–0.2810)	< 0.001	0.0099 (0.0064–0.0134)	< 0.001
+TyG-ABSI	0.0970 (0.0163–0.1776)	0.018	0.0020 (0.0030–0.0037)	0.022
+CTI	0.1750 (0.0947–0.2552)	< 0.001	0.0042 (0.0016–0.0068)	0.002

The basic model included age, gender, marital status, education level, Hu kou, smoking status, drinking status, hypertension, diabetes, dyslipidemia, kidney disease, stages of CKM syndrome, SBP, DBP, HDL-C, LDL-C, eGFR, UA, Cr, BUN, Hb, MCV, PCV, platelet count, WBC

NRI, net reclassification improvement; IDI, integrated discrimination improvement; Ref, reference; CI, confidence interval; TyG, triglyceride-glucose index; TyG-BMI, triglyceride glucose-body mass index; TyG-WC, triglyceride glucose-waist circumference; TyG-WHtR, triglyceride glucose-waist height ratio index; TyG-BRI, triglyceride glucose-body roundness index; TyG-WWI, triglyceride glucose-weight-adjusted waist index; TyG-CVAI, triglyceride glucose-Chinese visceral adiposity index; TyG-ABSI, triglyceride glucose -a body shape index; CTI, C-reactive protein-triglyceride-glucose index; SBP, systolic blood pressure; DBP, diastolic blood pressure; HDL-C, high-density lipoprotein cholesterol; LDL-C, low-density lipoprotein cholesterol; eGFR, estimated glomerular filtration rate; UA, uric acid; Cr, creatinine; BUN, blood urea nitrogen; Hb, hemoglobin; MCV, mean corpuscular volume; PCV, packed cell volume; WBC, white blood cell count

### Age-stratified analysis

**Fig. 5:**
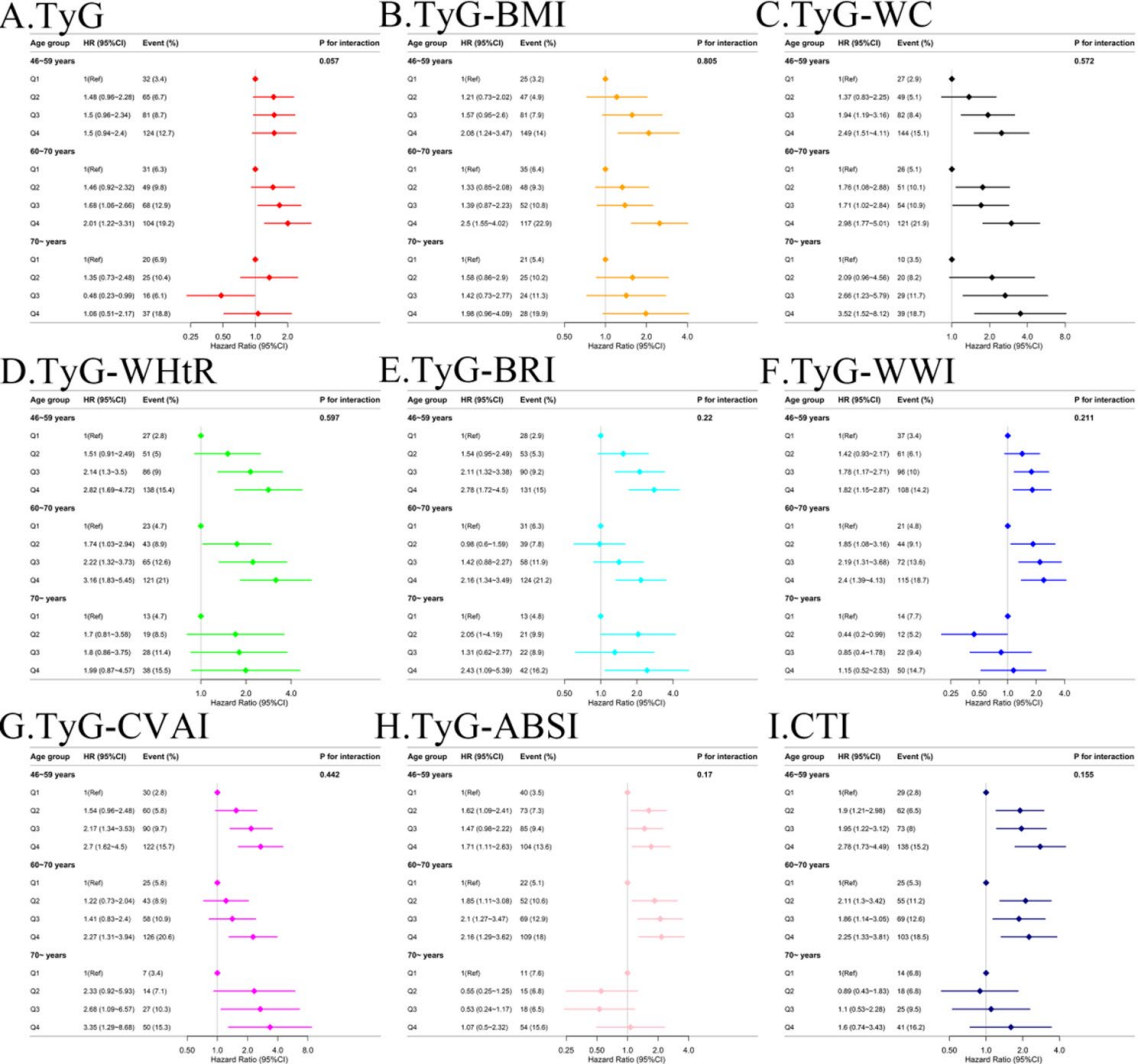
Age-stratified associations between TyG-related indices and the risk of CMM. Hazard ratios (HRs, squares) and 95% confidence intervals (CIs, horizontal lines) for the highest quartile (Q4) versus the lowest quartile (Q1) of each index are shown for three age strata: <60, 60–70, and ≥ 70 years. Analyses were based on the fully adjusted Model 4. **A** TyG, triglyceride-glucose index; **B** TyG-BMI, triglyceride glucose-body mass index; **C** TyG-WC, triglyceride glucose-waist circumference; **D** TyG-WHtR, triglyceride glucose-waist height ratio index; **E** TyG-BRI, triglyceride glucose-body roundness index; **F** TyG-WWI, triglyceride glucose-weight-adjusted waist index; **G** TyG-CVAI, triglyceride glucose-Chinese visceral adiposity index; **H** TyG-ABSI, triglyceride glucose–a body shape index; **I** CTI, C-reactive protein-triglyceride-glucose index. CMM, cardiometabolic multimorbidity; HR, hazard ratio; CI, confidence interval; Ref, reference

Age-stratified analysis revealed distinct patterns in the associations between TyG-related indices and CMM risk across age groups (Fig. 5). In participants aged 45–59 and 60–70 years, all nine TyG-related indices demonstrated clear dose-response relationships with CMM, characterized by monotonically increasing event rates and hazard ratios across ascending quartiles (Q1 to Q4). In contrast, among individuals aged ≥ 70 years, the strength of the association between most TyG-related indices and CMM was markedly attenuated compared with that in younger age groups. Only TyG-BMI, TyG-WC, TyG-WHtR, and TyG-CVAI maintained stable associations in this age group, whereas the remaining five indices showed inconsistent relationships with the risk of CMM. The p-interaction values for all nine indices were greater than 0.05, indicating that the strength of the associations between these indices and CMM risk did not differ significantly across the three age groups.

### Sensitivity analysis

To evaluate the robustness of our primary findings, we performed several sensitivity analyses. First, we excluded participants with diabetes at baseline to avoid potential confounding factors in the CMM outcome assessment (Table S9). Second, we repeated the analysis after excluding individuals with CKM syndrome at stage 0 who exhibited minimal cardiometabolic risk factors (Table S10). Third, we conducted analyses using multiple imputed datasets to account for the missing covariate data (Table S11). Finally, we performed a competing risk analysis using the Fine–Gray subdistribution hazard model (Table S16). The consistency of the findings across these analyses supports the robustness of our results.

## Discussion

In this large-scale, nationwide, and representative prospective cohort study, we comprehensively evaluated the association between nine TyG-related indices and the incidence of CMM in middle-aged and elderly Chinese patients with CKM syndrome stage 0–3. Our primary analysis revealed that all nine TyG-related indices were independent predictors of elevated CMM risk after multivariable adjustment. Notably, TyG-CVAI demonstrated the strongest predictive performance for CMM, stroke, and CHD, with the highest AUC values among all the indices. Moreover, NRI and IDI analyses demonstrated that these indices provided significant incremental predictive value beyond that of the comprehensive set of conventional risk factors included in our base model (Model 4). This finding supports the potential role of these markers in improving the clinical risk stratification of CMM.

We found that the association between TyG-related indices and CMM risk was substantially stronger than that between TyG-related indices and incident stroke or CHD. This discrepancy can be explained by several intuitive reasons. First, from a pathophysiological perspective, TyG indices are direct surrogates of insulin resistance and metabolic dysregulation, and the definition of CMM inherently includes diabetes mellitus. Therefore, a stronger alignment exists between the metabolic nature of the TyG indices and the CMM composition. In contrast, although stroke and CHD are critical consequences of metabolic disorders, their acute onset involves more complex and diverse immediate mechanisms (e.g., plaque rupture and thrombosis). The association between TyG indices and these specific cardiovascular events, although present, may be diluted by a longer and more indirect causal pathway. Second, from a study design standpoint, the composite endpoint of CMM had a higher number of events, providing greater statistical power to detect robust associations than the individual endpoints of stroke or CHD. Finally, methodological differences in outcome ascertainment should be considered. In this study, diabetes was defined using both self-reports and objective laboratory data, whereas stroke and CHD were ascertained primarily based on self-reported physician diagnoses. The latter method is more susceptible to recall bias and non-differential misclassification, which would tend to bias the effect estimates toward the null and may have partially attenuated the observed associations between stroke and CHD.

To address this need, the TyG index and related indices were developed as practical surrogates based on fasting triglyceride and glucose levels. The TyG index is well established for its association with cardiovascular disease [14, 44, 45], and its predictive value extends to a spectrum of other conditions [46]. To enhance risk stratification, composite indices derived from the TyG index by integrating anthropometric parameters (e.g., waist circumference, body mass index, and body shape index) have been developed. These indices capture cardiovascular risk from multiple dimensions and exhibit superior predictive ability [32, 47]. In terms of CMM prediction, Zhenyu et al. found that TyG and its related indicators were associated with CMM in young people [48], and Longyan et al. reported the predictive value of TyG-WHtR in the middle-aged and elderly populations in China [34]. Compared with their study, we focused our research on the middle-aged and elderly population with CKM syndrome stage 0–3. To the best of our knowledge, this is the first study to compare the predictive efficacy of nine TyG-related indices for CMM in this population. In our comparative analysis, TyG-CVAI demonstrated superior predictive performance for CMM, stroke, and CHD in both sexes. The enhanced performance of TyG-CVAI likely arises from its ability to integrate complementary pathophysiological pathways: the TyG component captures insulin resistance, while CVAI, a formula specifically developed for the Chinese population, incorporates age, sex, BMI, waist circumference, triglycerides, and HDL-C [49], providing a validated measure of visceral obesity and atherogenic lipid disorders [31, 50]. By concurrently reflecting insulin resistance, visceral adiposity, and dyslipidemia, TyG-CVAI offers a comprehensive biomarker strongly associated with CMM risk in individuals with stage 0–3 CKM syndrome. However, its clinical utility should be interpreted in context. Although the AUC for TyG-CVAI (0.679) reflects a moderate discriminative ability, its primary value lies in providing significant incremental information beyond conventional risk factors, as demonstrated by the NRI and IDI analyses. This enables improved risk stratification within the early CKM continuum (Stages 0–3), a heterogeneous, pre‑clinical population in which predicting a complex outcome such as CMM is inherently challenging. Moreover, TyG-CVAI is derived from routine, low‑cost measurements, making it a practical and accessible tool for initial screening in primary care. An elevated TyG‑CVAI can thus serve as a clinical red flag, prompting intensified management of modifiable risk factors (e.g., through lifestyle or pharmacologic interventions aimed at improving insulin sensitivity and reducing visceral adiposity) to halt progression along the CKM spectrum.

The robust associations observed between TyG-related indices and CMM risk in patients with CKM syndrome stages 0–3 can be understood through integrated pathophysiological pathways converging on multisystem injury. Insulin resistance (IR) is at the core of these conditions, and the TyG index serves as a practical surrogate marker. IR initiates a cascade of metabolic disturbances: systemic IR increases free fatty acid (FFA) flux, promotes ectopic lipid deposition in the liver, skeletal muscle, and pancreas (lipotoxicity), and sustains chronic hyperglycemia. These alterations collectively impair endothelial function, reduce nitric oxide bioavailability, and stimulate vascular smooth muscle cell proliferation, thereby creating a shared substrate for the development of multiple cardiometabolic diseases [51, 52].

Indices that combine TyG with measures of central obesity—such as TyG-WC, TyG-WHtR, and particularly TyG-CVAI—capture an amplified risk profile by accounting for visceral adiposity. Visceral adipose tissue is metabolically active, secreting pro-inflammatory cytokines (e.g., TNF-α, IL-6), releasing FFAs, and altering adipokine balance (e.g., reduced adiponectin). This not only exacerbates systemic IR but also promotes a prothrombotic state, drives atherosclerosis, and contributes to hypertension and renal dysfunction, which are key components of both CKM syndrome and CMM [53–55].

The inclusion of inflammation in the CTI further highlights the vicious cycle linking IR, dyslipidemia, and chronic inflammation. IR and hypertriglyceridemia can activate innate immune pathways, elevating inflammatory markers such as CRP levels. Inflammatory cytokines directly interfere with insulin signaling, worsening IR and accelerating vascular and renal damage. This synergy facilitates the progression from isolated metabolic disturbances to multiple concurrent diseases [56].

Within the conceptual framework of CKM syndrome, these mechanisms illustrate a self-sustaining cycle of metabolic dysregulation, visceral adiposity, and inflammation, which simultaneously injure the cardiovascular system and kidneys. TyG-related indices, especially composite markers such as TyG-CVAI, effectively quantify this multisystem crosstalk, explaining their superior ability to identify individuals at high risk of progressing to CMM, even in the early to intermediate stages of the syndrome.

The positive dose-response relationships observed in this study further strengthen the biological plausibility of the nine TyG-related indices. For most indices, the relationship with CMM risk was linear, reflecting a monotonic increase in risk across the entire range. This provides a theoretical basis for implementing universal risk assessments based on these indices in different populations. In contrast, the nonlinear relationships of TyG-ABSI and CTI with CMM risk imply a potential threshold effect, beyond which the contributions of adverse body morphology or systemic inflammation to CMM risk may accelerate markedly.

In age-stratified analyses, formal tests for interaction indicated no statistically significant effect modification by age for any of the nine TyG-related indices (all *p* > 0.05). Therefore, we cannot conclude that the strength of the association with CMM risk differs meaningfully across age groups. The apparent visual attenuation of associations in the oldest subgroup (≥ 70 years) observed in Fig. 5 may be attributable to factors such as reduced statistical power due to a smaller sample size and higher competing risks from non-CMM- mortality rather than a true biological interaction. Nevertheless, it is noteworthy that several indices, particularly TyG-BMI, TyG-WC, TyG-WHtR, and TyG-CVAI, maintained relatively stable point estimates across age strata, supporting their consistent predictive utility in this population. Our sensitivity analysis further confirmed the robustness of our findings. Excluding participants with baseline diabetes or CKM syndrome at stage 0 or performing multiple imputations for missing covariates did not alter the results. This consistency strengthens the robustness of our conclusions and supports the applicability of these measures in various clinical and research settings in the future.

However, our study had several limitations. First, despite the prospective cohort design and extensive multivariate adjustment, residual confounding cannot be ruled out. Although the constituent components of the stages (e.g., obesity, metabolic risk factors, CKD, and subclinical CVD) are long-standing and well-defined clinical entities, the specific integrative framework is new. This retrospective application could potentially lead to misclassifications, particularly in distinguishing adjacent stages. Second, the ascertainment of incident CHD and stroke was based on self-reported physician diagnoses without independent validation through objective measures. This approach is susceptible to recall bias and diagnostic misclassification, which may have led to measurement errors in these outcomes. Third, our findings were derived from a single, albeit nationally representative, Chinese cohort. The predictive model for CMM using TyG-CVAI requires external validation in independent, ideally multi-regional or multi-ethnic cohorts, to confirm its generalizability and transportability to other populations and healthcare settings. Fourth, the nine TyG-related metrics evaluated in this study exhibited collinearity due to their construction, indicating that their respective hazard ratios and performance metrics were not statistically independent. Fifth, the TyG-CVAI formula is complex and requires multiple parameters to be measured. Furthermore, the lack of universally standardized, clinically validated cutoff points beyond study-specific quartiles currently limits its direct application. Finally, although we addressed missing data through multiple imputations, unmeasured confounders such as dietary patterns, physical activity levels, weight changes, and genetic backgrounds may have influenced the observed associations.

Several promising directions for future research have emerged from our findings. First, the external validity of TyG-CVAI as a predictive tool needs to be established through external validation in independent, diverse cohorts, including those of other ethnicities and younger populations. Second, future studies should determine clinically applicable cut-off values for TyG-CVAI to guide risk stratification. Third, although our study established a robust epidemiological association, further mechanistic research is warranted to elucidate the precise biological pathways linking visceral adiposity, insulin resistance, and the progression of multimorbidity within the CKM syndrome framework. Fourth, investigating whether interventions that specifically lower TyG-CVAI can effectively reduce the incidence of CMM is a critical step toward translational application. Finally, future prospective studies should aim to collect and incorporate detailed longitudinal data on diet, physical activity, and sleep to better delineate the independent contribution of insulin resistance surrogates from these closely related lifestyle factors in the development of CMM.

## Conclusions

In this large-scale prospective cohort study, we established that nine TyG-related indices independently predicted an elevated risk of CMM among middle-aged and older Chinese adults with CKM syndrome stage 0–3. TyG-CVAI consistently demonstrated the strongest predictive capacity for CMM, stroke, and CHD, providing significant incremental value over conventional risk factors. These findings position TyG-CVAI as a promising and scientifically robust biomarker for CMM risk stratification in patients with CKM syndrome stages 0–3.

## Supplementary Information

Below is the link to the electronic supplementary material.

Supplementary Material 1

## Data Availability

All data used in this study were obtained from the CHARLS database (https://charls.pku.edu.cn/).

## Abbreviations

AHA: American Heart Association
BUN: Blood urea nitrogen
BMI: Body mass index
CHARLS: China health and retirement longitudinal study
CHD: Coronary heart disease
CI: Confidence interval
CKD: Chronic kidney disease
CKM: Cardiovascular-kidney-metabolic
CMM: Cardiometabolic multimorbidity
Cr: Creatinine
CRP: C-reactive protein
CVD: Cardiovascular disease
DBP: Diastolic blood pressure
eGFR: Estimated glomerular filtration rate
FBG: Fasting blood glucose
Hb: Hemoglobin
HbA1c: Hemoglobin A1c
HDL-C: High-density lipoprotein cholesterol
HR: Hazard ratio
IR: Insulin resistance
LDL-C: Low-density lipoprotein cholesterol
MCV: Mean corpuscular volume
NPV: Negative predictive value
PCV: Packed cell volume
PPV: Positive predictive value
RCS: Restricted cubic spline
SBP: Systolic blood pressure
SD: Standard deviation
TC: Total cholesterol
TG: Triglyceride
TyG: Triglyceride glucose
TyG-BMI: Triglyceride glucose-body mass index
TyG-WC: Triglyceride glucose-waist circumference
TyG-WHtR: Triglyceride glucose-waist height ratio index
TyG-BRI: Triglyceride glucose-body roundness index
TyG-WWI: Triglyceride glucose-weight-adjusted waist index
TyG-CVAI: Triglyceride glucose-Chinese visceral adiposity index
TyG-ABSI: Triglyceride glucose-a body shape index
CTI: C-reactive protein-triglyceride-glucose index
UA: Uric acid
WC: Waist circumference
WBC: White blood cell count
