# Supplementary material for: Relationship between nine triglyceride-glucose-related indices and cardiometabolic multimorbidity incidence in patients with cardiovascular-kidney-metabolic syndrome stage 0–3: a nationwide prospective cohort study
Source: Cardiovasc Diabetol. 2026 Jan 12;25:36. doi: 10.1186/s12933-026-03077-4 (PMC12879366; doi:10.1186/s12933-026-03077-4)
Supplement: Supplementary file 1 — Supplementary Material 1 [file 12933_2026_3077_MOESM1_ESM.docx]

**Supplementary Materials**

**Table S1** Definitions of CKM syndrome stage 0-3

**Table S2** Distribution of missing values for variables

**Table S3.1** Covariance analysis of TyG index and other variables based on model 4

**Table S3.2** Covariance analysis of TyG-BMI index and other variables based on model 4

**Table S3.3** Covariance analysis of TyG-WC index and other variables based on model 4

**Table S3.4** Covariance analysis of TyG-WHtR index and other variables based on model 4

**Table S3.5** Covariance analysis of TyG-BRI index and other variables based on model 4

**Table S3.6** Covariance analysis of TyG-WWI index and other variables based on model 4

**Table S3.7** Covariance analysis of TyG-CVAI index and other variables based on model 4

**Table S3.8** Covariance analysis of TyG-ABSI index and other variables based on model 4

**Table S3.9** Covariance analysis of CTI index and other variables based on model 4

**Table S4** Characteristics of 6,867 participants based on the occurrence of CMM

**Table S5** Associations between TyG-related indices and stroke risk in CKM syndrome stage 0-3

**Table S6** Associations between TyG-related indices and CHD risk in CKM syndrome stage 0-3

**Table S7** Incremental predictive value of TyG-related indices for stroke in CKM syndrome stage 0-3

**Table S8** Incremental predictive value of TyG-related indices for CHD in CKM syndrome stage 0-3

**Table S9** Associations between TyG-related indices and CMM risk in CKM syndrome stage 0-3 without diabetes at baseline

**Table S10** Associations between TyG-related indices and CMM risk in CKM syndrome stage 1-3

**Table S11** Associations between TyG-related indices and CMM risk in CKM syndrome stage 0-3 after including individuals with missing data

**Table S12** Optimal thresholds, sensitivity, specificity, NPV and PPV of TyG-related indices for predicting CMM, stroke, and CHD

**Table S13** Schoenfeld residual test results for the Cox proportional hazards models of the associations between TyG-related indices and CMM in patients with CKM syndrome stage 0-3

**Table S14** Schoenfeld residual test results for the Cox proportional hazards models of the associations between TyG-related indices and stroke in patients with CKM syndrome stage 0-3

**Table S15** Schoenfeld residual test results for the Cox proportional hazards models of the associations between TyG-related indices and CHD in patients with CKM syndrome stage 0-3

**Table S16** Associations between TyG-related indices and incident CMM using Fine-Gray competing risk regression in participants with CKM syndrome stage 0-3

**Fig.S1** Dose-response relationships between TyG-related indices and stroke risk

**Fig.S2** Dose-response relationships between TyG-related indices and CHD risk

**Fig.S3** Kaplan-Meier curves for cumulative incidence of stroke by quartiles of TyG-related indices

**Fig.S4** Kaplan-Meier curves for cumulative incidence of CHD by quartiles of TyG-related indices

**Fig.S5** Pairwise comparison of the AUC for predicting CMM among TyG-related indices using the DeLong test

| **Table S1** Definitions of CKM syndrome stage 0-3[1] | |
| --- | --- |
| Stage 0 | **No CKM risk factors** |
| Stage 1 | **Excess or dysfunctional adiposity** (any one of the following): |
|  | BMI≥24kg/m2; |
|  | WC ≥ 80/90 cm in female/male; |
|  | FBG ≥ 100-124 mg/dL or HbA1c between 5.7% and 6.4%. |
| Stage 2 | **Individuals with metabolic risk factors (**at least three of the following five criteria): |
|  | **1.Diabetes**: FBG ≥ 126 mg/dl or HbA1c ≥ 6.5%, and/or a self-reported physician-diagnosed, and/or taking hypoglycemic agents; |
|  | **2.Hypertension**: a self-reported physician-diagnosed, and/or any antihypertensive medication use, and/or an average systolic/diastolic blood pressure (SBP/DBP) ≥ 140/90 mmHg; |
|  | **3.Metabolic syndrome**: Meet any three or more of the five: (1) WC ≥ 90 cm for males and ≥ 80 cm for females; (2) FBG ≥ 100mg/dl; (3) SBP/DBP ≥ 130/80 mmHg and/or use of antihypertensive medications; (4) TG ≥ 150mg/dl; (5) HDL-C< 40mg/dl for males and <50mg/dl for females; |
|  | **4.Dyslipidemia**: a self-reported physician-diagnosed, and/or current use of lipid-lowering drugs, and/or TC ≥ 240 mg/dl, TG≥ 135 mg/dl, HDL-C < 40 mg/dl, LDL-C ≥ 160 mg/dl; |
|  | **5.CKD**: self-reported history of chronic kidney disease or eGFR < 60 ml/min/1.73m^2^. |
| Stage 3 | **Subclinical CVD** (any one of the following): |
|  | **1.**eGFR < 30 ml/min/1.73m2; |
|  | **2.**Framingham risk score ≥ 20%. |

Reference of **Table S1**

1. Ndumele CE, Rangaswami J, Chow SL, Neeland IJ, Tuttle KR, Khan SS, Coresh J, Mathew RO, Baker-Smith CM, Carnethon MR *et al*: **Cardiovascular-Kidney-Metabolic Health: A Presidential Advisory From the American Heart Association**. *Circulation* 2023, **148**(20):1606-1635.

| **Table S2** Distribution of missing values for variables | | |
| --- | --- | --- |
| Variables | The number of missing data | Percent (%) |
| Gender | 6 | 0.087 |
| Education level | 2 | 0.029 |
| Hukou | 4 | 0.058 |
| Smoking status | 1 | 0.015 |
| Hypertension | 18 | 0.262 |
| SBP | 60 | 0.874 |
| DBP | 15 | 0.218 |
| FBG | 0 | 0 |
| HbA1c | 59 | 0.859 |
| Dyslipidemia | 52 | 0.757 |
| TC | 0 | 0 |
| TG | 0 | 0 |
| LDL-C | 13 | 0.189 |
| HDL-C | 1 | 0.015 |
| Kidney disease | 15 | 0.218 |
| UA | 0 | 0 |
| Cr | 1 | 0.015 |
| BUN | 0 | 0 |
| eGFR | 0 | 0 |
| CRP | 0 | 0 |
| Platelet count | 137 | 1.995 |
| Hb | 2 | 0.029 |
| MCV | 0 | 0 |
| PCV | 3 | 0.044 |
| WBC | 4 | 0.058 |
| Height | 0 | 0 |
| Weight | 0 | 0 |
| WC | 0 | 0 |
| SBP, systolic blood pressure; DBP, diastolic blood pressure; HDL-C, high-density lipoprotein cholesterol; LDL-C, low-density lipoprotein cholesterol; eGFR, estimated glomerular filtration rate; UA, uric acid; Cr, creatinine; BUN, blood urea nitrogen; Hb, hemoglobin; MCV, mean corpuscular volume; PCV, packed cell volume; WBC, white blood cell count, TC, total cholesterol; TG, triglyceride, FBG, fasting blood glucose, WC, waist circumference; CRP, C-reactive protein | | |

| **Table S3.1** Covariance analysis of TyG index and other variables based on model 4 | | | |
| --- | --- | --- | --- |
| Variable | GVIF | DF | GVIF^(1/(2*Df)) |
| TyG | 1.938 | 1 | 1.392 |
| Age | 1.763 | 1 | 1.328 |
| Gender | 3.013 | 1 | 1.736 |
| Marital status | 1.147 | 1 | 1.071 |
| Education level | 1.205 | 2 | 1.048 |
| Hukou | 1.146 | 1 | 1.07 |
| Smoking status | 2.193 | 1 | 1.481 |
| Drinking status | 1.382 | 1 | 1.176 |
| Hypertension | 1.833 | 1 | 1.354 |
| SBP | 2.38 | 1 | 1.543 |
| DBP | 2.009 | 1 | 1.417 |
| Dyslipidemia | 1.905 | 1 | 1.38 |
| LDL-C | 1.262 | 1 | 1.124 |
| HDL-C | 1.595 | 1 | 1.263 |
| Kidney disease | 1.027 | 1 | 1.014 |
| UA | 1.485 | 1 | 1.219 |
| Cr | 2.494 | 1 | 1.579 |
| BUN | 1.172 | 1 | 1.083 |
| eGFR | 2.222 | 1 | 1.491 |
| Hb | 1.783 | 1 | 1.335 |
| MCV | 1.178 | 1 | 1.085 |
| PCV | 1.896 | 1 | 1.377 |
| Platelet count | 1.148 | 1 | 1.071 |
| WBC | 1.116 | 1 | 1.056 |
| Stages of CKM syndrome | 3.319 | 3 | 1.221 |
| TyG, triglyceride-glucose index; SBP, systolic blood pressure; DBP, diastolic blood pressure; HDL-C, high-density lipoprotein cholesterol; LDL-C, low-density lipoprotein cholesterol; eGFR, estimated glomerular filtration rate; UA, uric acid; Cr, creatinine; BUN, blood urea nitrogen; Hb, hemoglobin; MCV, mean corpuscular volume; PCV, packed cell volume; WBC, white blood cell count; CKM syndrome, cardiovascular kidney-metabolic syndrome | | | |

| **Table S3.2** Covariance analysis of TyG-BMI index and other variables based on model 4 | | | |
| --- | --- | --- | --- |
| Variable | GVIF | DF | GVIF^(1/(2*Df)) |
| TyG-BMI | 1.705 | 1 | 1.306 |
| Age | 1.848 | 1 | 1.359 |
| Gender | 2.958 | 1 | 1.72 |
| Marital status | 1.145 | 1 | 1.07 |
| Education level | 1.199 | 2 | 1.046 |
| Hukou | 1.146 | 1 | 1.071 |
| Smoking status | 2.244 | 1 | 1.498 |
| Drinking status | 1.393 | 1 | 1.18 |
| Hypertension | 1.838 | 1 | 1.356 |
| SBP | 2.488 | 1 | 1.577 |
| DBP | 2.046 | 1 | 1.431 |
| Dyslipidemia | 1.632 | 1 | 1.277 |
| LDL-C | 1.206 | 1 | 1.098 |
| HDL-C | 1.59 | 1 | 1.261 |
| Kidney disease | 1.028 | 1 | 1.014 |
| UA | 1.504 | 1 | 1.226 |
| Cr | 2.189 | 1 | 1.479 |
| BUN | 1.186 | 1 | 1.089 |
| eGFR | 2.026 | 1 | 1.423 |
| Hb | 1.824 | 1 | 1.351 |
| MCV | 1.172 | 1 | 1.083 |
| PCV | 1.936 | 1 | 1.392 |
| Platelet count | 1.153 | 1 | 1.074 |
| WBC | 1.117 | 1 | 1.057 |
| Stages of CKM syndrome | 3.266 | 3 | 1.218 |
| TyG-BMI, triglyceride glucose-body mass index; SBP, systolic blood pressure; DBP, diastolic blood pressure; HDL-C, high-density lipoprotein cholesterol; LDL-C, low-density lipoprotein cholesterol; eGFR, estimated glomerular filtration rate; UA, uric acid; Cr, creatinine; BUN, blood urea nitrogen; Hb, hemoglobin; MCV, mean corpuscular volume; PCV, packed cell volume; WBC, white blood cell count; CKM syndrome, cardiovascular kidney-metabolic syndrome | | | |

| **Table S3.3** Covariance analysis of TyG-WC index and other variables based on model 4 | | | |
| --- | --- | --- | --- |
| Variable | GVIF | DF | GVIF^(1/(2*Df)) |
| TyG-WC | 1.723 | 1 | 1.312 |
| Age | 1.765 | 1 | 1.329 |
| Gender | 2.911 | 1 | 1.706 |
| Marital status | 1.148 | 1 | 1.071 |
| Education level | 1.196 | 2 | 1.046 |
| Hukou | 1.142 | 1 | 1.068 |
| Smoking status | 2.226 | 1 | 1.492 |
| Drinking status | 1.395 | 1 | 1.181 |
| Hypertension | 1.836 | 1 | 1.355 |
| SBP | 2.482 | 1 | 1.575 |
| DBP | 2.024 | 1 | 1.423 |
| Dyslipidemia | 1.671 | 1 | 1.293 |
| LDL-C | 1.206 | 1 | 1.098 |
| HDL-C | 1.618 | 1 | 1.272 |
| Kidney disease | 1.028 | 1 | 1.014 |
| UA | 1.504 | 1 | 1.226 |
| Cr | 2.192 | 1 | 1.481 |
| BUN | 1.179 | 1 | 1.086 |
| eGFR | 2.028 | 1 | 1.424 |
| Hb | 1.814 | 1 | 1.347 |
| MCV | 1.175 | 1 | 1.084 |
| PCV | 1.917 | 1 | 1.384 |
| Platelet count | 1.155 | 1 | 1.075 |
| WBC | 1.117 | 1 | 1.057 |
| Stages of CKM syndrome | 3.247 | 3 | 1.217 |
| TyG-WC, triglyceride glucose-waist circumference index; SBP, systolic blood pressure; DBP, diastolic blood pressure; HDL-C, high-density lipoprotein cholesterol; LDL-C, low-density lipoprotein cholesterol; eGFR, estimated glomerular filtration rate; UA, uric acid; Cr, creatinine; BUN, blood urea nitrogen; Hb, hemoglobin; MCV, mean corpuscular volume; PCV, packed cell volume; WBC, white blood cell count; CKM syndrome, cardiovascular kidney-metabolic syndrome | | | |

| **Table S3.4** Covariance analysis of TyG-WHtR index and other variables based on model 4 | | | |
| --- | --- | --- | --- |
| Variable | GVIF | DF | GVIF^(1/(2*Df)) |
| TyG-WHtR | 1.858 | 1 | 1.363 |
| Age | 1.767 | 1 | 1.329 |
| Gender | 3.183 | 1 | 1.784 |
| Marital status | 1.145 | 1 | 1.07 |
| Education level | 1.2 | 2 | 1.047 |
| Hukou | 1.137 | 1 | 1.066 |
| Smoking status | 2.247 | 1 | 1.499 |
| Drinking status | 1.39 | 1 | 1.179 |
| Hypertension | 1.843 | 1 | 1.358 |
| SBP | 2.504 | 1 | 1.582 |
| DBP | 2.028 | 1 | 1.424 |
| Dyslipidemia | 1.682 | 1 | 1.297 |
| LDL-C | 1.213 | 1 | 1.101 |
| HDL-C | 1.588 | 1 | 1.26 |
| Kidney disease | 1.026 | 1 | 1.013 |
| UA | 1.504 | 1 | 1.226 |
| Cr | 2.261 | 1 | 1.504 |
| BUN | 1.179 | 1 | 1.086 |
| eGFR | 2.072 | 1 | 1.439 |
| Hb | 1.816 | 1 | 1.348 |
| MCV | 1.176 | 1 | 1.084 |
| PCV | 1.917 | 1 | 1.385 |
| Platelet count | 1.154 | 1 | 1.074 |
| WBC | 1.12 | 1 | 1.058 |
| Stages of CKM syndrome | 3.247 | 3 | 1.217 |
| TyG-WHtR, triglyceride glucose-waist height ratio index; SBP, systolic blood pressure; DBP, diastolic blood pressure; HDL-C, high-density lipoprotein cholesterol; LDL-C, low-density lipoprotein cholesterol; eGFR, estimated glomerular filtration rate; UA, uric acid; Cr, creatinine; BUN, blood urea nitrogen; Hb, hemoglobin; MCV, mean corpuscular volume; PCV, packed cell volume; WBC, white blood cell count; CKM syndrome, cardiovascular kidney-metabolic syndrome | | | |

| **Table S3.5** Covariance analysis of TyG-BRI index and other variables based on model 4 | | | |
| --- | --- | --- | --- |
| Variable | GVIF | DF | GVIF^(1/(2*Df)) |
| TyG-BRI | 1.629 | 1 | 1.276 |
| Age | 1.786 | 1 | 1.336 |
| Gender | 3.153 | 1 | 1.776 |
| Marital status | 1.142 | 1 | 1.069 |
| Education level | 1.199 | 2 | 1.046 |
| Hukou | 1.137 | 1 | 1.066 |
| Smoking status | 2.255 | 1 | 1.502 |
| Drinking status | 1.382 | 1 | 1.176 |
| Hypertension | 1.864 | 1 | 1.365 |
| SBP | 2.579 | 1 | 1.606 |
| DBP | 2.056 | 1 | 1.434 |
| Dyslipidemia | 1.615 | 1 | 1.271 |
| LDL-C | 1.218 | 1 | 1.104 |
| HDL-C | 1.538 | 1 | 1.24 |
| Kidney disease | 1.025 | 1 | 1.013 |
| UA | 1.501 | 1 | 1.225 |
| Cr | 2.23 | 1 | 1.493 |
| BUN | 1.183 | 1 | 1.088 |
| eGFR | 2.053 | 1 | 1.433 |
| Hb | 1.815 | 1 | 1.347 |
| MCV | 1.173 | 1 | 1.083 |
| PCV | 1.916 | 1 | 1.384 |
| Platelet count | 1.156 | 1 | 1.075 |
| WBC | 1.118 | 1 | 1.057 |
| Stages of CKM syndrome | 3.236 | 3 | 1.216 |
| TyG-BRI, triglyceride glucose-body roundness index; SBP, systolic blood pressure; DBP, diastolic blood pressure; HDL-C, high-density lipoprotein cholesterol; LDL-C, low-density lipoprotein cholesterol; eGFR, estimated glomerular filtration rate; UA, uric acid; Cr, creatinine; BUN, blood urea nitrogen; Hb, hemoglobin; MCV, mean corpuscular volume; PCV, packed cell volume; WBC, white blood cell count; CKM syndrome, cardiovascular kidney-metabolic syndrome | | | |

| **Table S3.6** Covariance analysis of TyG-WWI index and other variables based on model 4 | | | |
| --- | --- | --- | --- |
| Variable | GVIF | DF | GVIF^(1/(2*Df)) |
| TyG-WWI | 1.85 | 1 | 1.36 |
| Age | 1.835 | 1 | 1.355 |
| Gender | 3.259 | 1 | 1.805 |
| Marital status | 1.147 | 1 | 1.071 |
| Education level | 1.203 | 2 | 1.047 |
| Hukou | 1.14 | 1 | 1.068 |
| Smoking status | 2.216 | 1 | 1.489 |
| Drinking status | 1.377 | 1 | 1.173 |
| Hypertension | 1.855 | 1 | 1.362 |
| SBP | 2.501 | 1 | 1.581 |
| DBP | 2.03 | 1 | 1.425 |
| Dyslipidemia | 1.745 | 1 | 1.321 |
| LDL-C | 1.235 | 1 | 1.111 |
| HDL-C | 1.553 | 1 | 1.246 |
| Kidney disease | 1.026 | 1 | 1.013 |
| UA | 1.492 | 1 | 1.221 |
| Cr | 2.42 | 1 | 1.556 |
| BUN | 1.176 | 1 | 1.084 |
| eGFR | 2.178 | 1 | 1.476 |
| Hb | 1.793 | 1 | 1.339 |
| MCV | 1.179 | 1 | 1.086 |
| PCV | 1.9 | 1 | 1.379 |
| Platelet count | 1.154 | 1 | 1.074 |
| WBC | 1.116 | 1 | 1.056 |
| Stages of CKM syndrome | 3.246 | 3 | 1.217 |
| TyG-WWI, triglyceride glucose-weight-adjusted waist index; SBP, systolic blood pressure; DBP, diastolic blood pressure; HDL-C, high-density lipoprotein cholesterol; LDL-C, low-density lipoprotein cholesterol; eGFR, estimated glomerular filtration rate; UA, uric acid; Cr, creatinine; BUN, blood urea nitrogen; Hb, hemoglobin; MCV, mean corpuscular volume; PCV, packed cell volume; WBC, white blood cell count; CKM syndrome, cardiovascular kidney-metabolic syndrome | | | |

| **Table S3.7** Covariance analysis of TyG-CVAI index and other variables based on model 4 | | | |
| --- | --- | --- | --- |
| Variable | GVIF | DF | GVIF^(1/(2*Df)) |
| TyG-CVAI | 1.998 | 1 | 1.414 |
| Age | 1.808 | 1 | 1.345 |
| Gender | 2.914 | 1 | 1.707 |
| Marital status | 1.151 | 1 | 1.073 |
| Education level | 1.196 | 2 | 1.046 |
| Hukou | 1.143 | 1 | 1.069 |
| Smoking status | 2.218 | 1 | 1.489 |
| Drinking status | 1.391 | 1 | 1.179 |
| Hypertension | 1.854 | 1 | 1.362 |
| SBP | 2.542 | 1 | 1.594 |
| DBP | 2.051 | 1 | 1.432 |
| Dyslipidemia | 1.652 | 1 | 1.285 |
| LDL-C | 1.205 | 1 | 1.098 |
| HDL-C | 1.795 | 1 | 1.34 |
| Kidney disease | 1.028 | 1 | 1.014 |
| UA | 1.508 | 1 | 1.228 |
| Cr | 2.164 | 1 | 1.471 |
| BUN | 1.176 | 1 | 1.084 |
| eGFR | 2.011 | 1 | 1.418 |
| Hb | 1.829 | 1 | 1.352 |
| MCV | 1.174 | 1 | 1.084 |
| PCV | 1.932 | 1 | 1.39 |
| Platelet count | 1.155 | 1 | 1.075 |
| WBC | 1.118 | 1 | 1.057 |
| Stages of CKM syndrome | 3.217 | 3 | 1.215 |
| TyG-CVAI, triglyceride glucose-Chinese visceral adiposity index; SBP, systolic blood pressure; DBP, diastolic blood pressure; HDL-C, high-density lipoprotein cholesterol; LDL-C, low-density lipoprotein cholesterol; eGFR, estimated glomerular filtration rate; UA, uric acid; Cr, creatinine; BUN, blood urea nitrogen; Hb, hemoglobin; MCV, mean corpuscular volume; PCV, packed cell volume; WBC, white blood cell count; CKM syndrome, cardiovascular kidney-metabolic syndrome | | | |

| **Table S3.8** Covariance analysis of TyG-ABSI index and other variables based on model 4 | | | |
| --- | --- | --- | --- |
| Variable | GVIF | DF | GVIF^(1/(2*Df)) |
| TyG-ABSI | 1.588 | 1 | 1.26 |
| Age | 1.833 | 1 | 1.354 |
| Gender | 3.043 | 1 | 1.745 |
| Marital status | 1.148 | 1 | 1.071 |
| Education level | 1.202 | 2 | 1.047 |
| Hukou | 1.142 | 1 | 1.068 |
| Smoking status | 2.198 | 1 | 1.482 |
| Drinking status | 1.373 | 1 | 1.172 |
| Hypertension | 1.858 | 1 | 1.363 |
| SBP | 2.494 | 1 | 1.579 |
| DBP | 2.036 | 1 | 1.427 |
| Dyslipidemia | 1.742 | 1 | 1.32 |
| LDL-C | 1.245 | 1 | 1.116 |
| HDL-C | 1.54 | 1 | 1.241 |
| Kidney disease | 1.027 | 1 | 1.013 |
| UA | 1.487 | 1 | 1.219 |
| Cr | 2.427 | 1 | 1.558 |
| BUN | 1.176 | 1 | 1.084 |
| eGFR | 2.183 | 1 | 1.477 |
| Hb | 1.784 | 1 | 1.336 |
| MCV | 1.179 | 1 | 1.086 |
| PCV | 1.895 | 1 | 1.377 |
| Platelet count | 1.153 | 1 | 1.074 |
| WBC | 1.113 | 1 | 1.055 |
| Stages of CKM syndrome | 3.239 | 3 | 1.216 |
| TyG-ABSI, triglyceride glucose -a body shape index; SBP, systolic blood pressure; DBP, diastolic blood pressure; HDL-C, high-density lipoprotein cholesterol; LDL-C, low-density lipoprotein cholesterol; eGFR, estimated glomerular filtration rate; UA, uric acid; Cr, creatinine; BUN, blood urea nitrogen; Hb, hemoglobin; MCV, mean corpuscular volume; PCV, packed cell volume; WBC, white blood cell count; CKM syndrome, cardiovascular kidney-metabolic syndrome | | | |

| **Table S3.9** Covariance analysis of CTI index and other variables based on model 4 | | | |
| --- | --- | --- | --- |
| Variable | GVIF | DF | GVIF^(1/(2*Df)) |
| CTI | 1.769 | 1 | 1.33 |
| Age | 1.752 | 1 | 1.323 |
| Gender | 3.009 | 1 | 1.735 |
| Marital status | 1.145 | 1 | 1.07 |
| Education level | 1.197 | 2 | 1.046 |
| Hukou | 1.141 | 1 | 1.068 |
| Smoking status | 2.207 | 1 | 1.486 |
| Drinking status | 1.385 | 1 | 1.177 |
| Hypertension | 1.837 | 1 | 1.355 |
| SBP | 2.413 | 1 | 1.554 |
| DBP | 2.023 | 1 | 1.422 |
| Dyslipidemia | 1.766 | 1 | 1.329 |
| LDL-C | 1.236 | 1 | 1.112 |
| HDL-C | 1.599 | 1 | 1.265 |
| Kidney disease | 1.026 | 1 | 1.013 |
| UA | 1.5 | 1 | 1.225 |
| Cr | 2.448 | 1 | 1.565 |
| BUN | 1.175 | 1 | 1.084 |
| eGFR | 2.203 | 1 | 1.484 |
| Hb | 1.796 | 1 | 1.34 |
| MCV | 1.175 | 1 | 1.084 |
| PCV | 1.909 | 1 | 1.382 |
| Platelet count | 1.149 | 1 | 1.072 |
| WBC | 1.177 | 1 | 1.085 |
| Stages of CKM syndrome | 3.274 | 3 | 1.219 |
| CTI, C-reactive protein-triglyceride-glucose index; SBP, systolic blood pressure; DBP, diastolic blood pressure; HDL-C, high-density lipoprotein cholesterol; LDL-C, low-density lipoprotein cholesterol; eGFR, estimated glomerular filtration rate; UA, uric acid; Cr, creatinine; BUN, blood urea nitrogen; Hb, hemoglobin; MCV, mean corpuscular volume; PCV, packed cell volume; WBC, white blood cell count; CKM syndrome, cardiovascular kidney-metabolic syndrome | | | |

| **Table S4** Characteristics of 6,867 participants based on the occurrence of CMM | | | | |
| --- | --- | --- | --- | --- |
| Variables | Total | CMM |  | P |
|  |  | No | Yes |  |
| n | 6867 | 6215 | 652 |  |
| Age Mean ± SD | 59.2 ± 9.0 | 59.1 ± 9.1 | 60.7 ± 8.1 | < 0.001 |
| Gender (%) |  |  |  | < 0.001 |
| Male | 3270 (47.6) | 3001 (48.3) | 269 (41.3) |  |
| Female | 3597 (52.4) | 3214 (51.7) | 383 (58.7) |  |
| Marital status (%) |  |  |  | 0.411 |
| Married | 6091 (88.7) | 5519 (88.8) | 572 (87.7) |  |
| Others | 776 (11.3) | 696 (11.2) | 80 (12.3) |  |
| Education level (%) |  |  |  | 0.71 |
| Secondary school or below | 6212 (90.5) | 5627 (90.5) | 585 (89.7) |  |
| High school | 575 (8.4) | 515 (8.3) | 60 (9.2) |  |
| College or above | 80 (1.2) | 73 (1.2) | 7 (1.1) |  |
| Hukou (%) |  |  |  | 0.003 |
| Agriculture | 5853 (85.2) | 5323 (85.6) | 530 (81.3) |  |
| Others | 1014 (14.8) | 892 (14.4) | 122 (18.7) |  |
| Smoking status (%) |  |  |  | 0.071 |
| No | 4145 (60.4) | 3730 (60) | 415 (63.7) |  |
| Yes | 2722 (39.6) | 2485 (40) | 237 (36.3) |  |
| Drinking status (%) |  |  |  | 0.075 |
| No | 4545 (66.2) | 4093 (65.9) | 452 (69.3) |  |
| Yes | 2322 (33.8) | 2122 (34.1) | 200 (30.7) |  |
| Hypertension (%) |  |  |  | < 0.001 |
| No | 4309 (62.7) | 4026 (64.8) | 283 (43.4) |  |
| Yes | 2558 (37.3) | 2189 (35.2) | 369 (56.6) |  |
| Diabetes (%) |  |  |  | < 0.001 |
| No | 6462 (94.1) | 5946 (95.7) | 516 (79.1) |  |
| Yes | 405 (5.9) | 269 (4.3) | 136 (20.9) |  |
| Dyslipidemia (%) |  |  |  | < 0.001 |
| No | 3697 (53.8) | 3453 (55.6) | 244 (37.4) |  |
| Yes | 3170 (46.2) | 2762 (44.4) | 408 (62.6) |  |
| Kidney disease (%) |  |  |  | 0.003 |
| No | 6456 (94.0) | 5860 (94.3) | 596 (91.4) |  |
| Yes | 411 (6.0) | 355 (5.7) | 56 (8.6) |  |
| SBP, mmHg | 128.8 ± 22.4 | 128.1 ± 22.3 | 135.9 ± 21.5 | < 0.001 |
| DBP, mmHg | 75.0 ± 12.1 | 74.7 ± 12.0 | 77.9 ± 12.1 | < 0.001 |
| FBG, mg/dL | 108.3 ± 27.6 | 106.7 ± 24.8 | 124.4 ± 43.5 | < 0.001 |
| HbA1c (%) | 5.2 ± 0.7 | 5.2 ± 0.7 | 5.6 ± 1.1 | < 0.001 |
| HDL-C, mg/dL | 51.7 ± 15.0 | 52.1 ± 15.0 | 48.3 ± 14.3 | < 0.001 |
| LDL-C, mg/dL | 117.2 ± 34.3 | 116.5 ± 33.9 | 123.5 ± 37.5 | < 0.001 |
| TC, mg/dL | 193.5 ± 37.5 | 192.6 ± 37.2 | 201.6 ± 39.6 | < 0.001 |
| TG, mg/dL | 126.1 ± 78.7 | 123.6 ± 76.9 | 149.3 ± 91.1 | < 0.001 |
| eGFR, mL/min per 1.73 m^2^ | 108.5 ± 27.7 | 108.8 ± 27.5 | 106.2 ± 30.0 | 0.026 |
| UA, mg/dL | 4.4 ± 1.2 | 4.4 ± 1.2 | 4.5 ± 1.2 | 0.251 |
| Cr, mg/dL | 0.8 ± 0.2 | 0.8 ± 0.2 | 0.8 ± 0.2 | 0.682 |
| BUN, mg/dL | 15.7 ± 4.5 | 15.8 ± 4.5 | 15.5 ± 4.4 | 0.207 |
| Height, m | 157.9 ± 8.2 | 157.9 ± 8.2 | 157.5 ± 8.2 | 0.201 |
| Weight, kg | 58.2 ± 10.3 | 57.8 ± 10.1 | 61.6 ± 11.1 | < 0.001 |
| WC, cm | 84.8 ± 9.5 | 84.3 ± 9.3 | 89.4 ± 9.7 | < 0.001 |
| BMI, kg/m^2^ | 23.3 ± 3.4 | 23.1 ± 3.3 | 24.8 ± 3.7 | < 0.001 |
| CRP, mg/L | 1.0 (0.5, 2.1) | 1.0 (0.5, 2.0) | 1.4 (0.7, 2.6) | < 0.001 |
| Hb, g/dL | 14.4 ± 2.2 | 14.4 ± 2.2 | 14.5 ± 2.2 | 0.316 |
| MCV, fL | 90.7 ± 8.5 | 90.7 ± 8.5 | 90.8 ± 8.4 | 0.915 |
| PCV (%) | 41.7 ± 6.1 | 41.6 ± 6.1 | 41.8 ± 5.9 | 0.576 |
| Platelet count, 10^9^/L | 211.6 ± 76.1 | 210.5 ± 72.1 | 222.2 ± 106.7 | < 0.001 |
| WBC, 10^9^/L | 6.3 ± 1.9 | 6.2 ± 1.9 | 6.6 ± 2.1 | < 0.001 |
| Data are presented as mean ± SD or n (percentage) CMM, cardiometabolic multimorbidity; SBP, systolic blood pressure; DBP, diastolic blood pressure; FBG, fasting blood glucose; HbA1c, hemoglobin A1c; HDL-C, high-density lipoprotein cholesterol; LDL-C, low-density lipoprotein cholesterol; TC, total cholesterol; TG, triglyceride; eGFR, estimated glomerular filtration rate; UA, uric acid; Cr, creatinine; BUN, blood urea nitrogen; WC, waist circumference; BMI, body mass index; CRP, C-reactive protein; Hb, hemoglobin; MCV, mean corpuscular volume; PCV, packed cell volume; WBC, white blood cell count; SD, standard deviation | | | | |

| **Fig.S5** Associations between TyG-related indices and stroke risk in CKM syndrome stage 0-3 | | | | | | | | | | | |
| --- | --- | --- | --- | --- | --- | --- | --- | --- | --- | --- | --- |
|  | Case | Crude |  | Model 1 |  | Model 2 |  | Model 3 |  | Model 4 |  |
|  |  | HR (95%CI) | P | HR (95%CI) | P | HR (95%CI) | P | HR (95%CI) | P | HR (95%CI) | P |
| TyG |  |  |  |  |  |  |  |  |  |  |  |
| Q1 | 91 (5.3) | 1(Ref) |  |  |  |  |  |  |  |  |  |
| Q2 | 142 (8.3) | 1.55 (1.19~2.02) | 0.001 | 1.6 (1.23~2.08) | <0.001 | 1.59 (1.22~2.08) | 0.001 | 1.49 (1.14~1.94) | 0.003 | 1.4 (1.07~1.83) | 0.014 |
| Q3 | 181 (10.5) | 2.03 (1.58~2.62) | <0.001 | 2.07 (1.6~2.66) | <0.001 | 2.06 (1.6~2.66) | <0.001 | 1.72 (1.32~2.24) | <0.001 | 1.5 (1.14~1.98) | 0.004 |
| Q4 | 175 (10.2) | 1.97 (1.53~2.54) | <0.001 | 2.08 (1.61~2.68) | <0.001 | 2.07 (1.6~2.67) | <0.001 | 1.39 (1.04~1.87) | 0.029 | 1.25 (0.91~1.7) | 0.167 |
| Trend test | 589 (8.6) | 1.23 (1.15~1.33) | <0.001 | 1.25 (1.16~1.35) | <0.001 | 1.25 (1.16~1.35) | <0.001 | 1.11 (1.01~1.22) | 0.024 | 1.07 (0.97~1.18) | 0.185 |
| TyG-BMI |  |  |  |  |  |  |  |  |  |  |  |
| Q1 | 91 (5.3) | 1(Ref) |  |  |  |  |  |  |  |  |  |
| Q2 | 138 (8) | 1.47 (1.13~1.91) | 0.004 | 1.64 (1.26~2.14) | <0.001 | 1.66 (1.27~2.16) | <0.001 | 1.52 (1.16~1.99) | 0.002 | 1.44 (1.09~1.89) | 0.01 |
| Q3 | 167 (9.7) | 1.79 (1.39~2.32) | <0.001 | 2.11 (1.63~2.73) | <0.001 | 2.13 (1.65~2.76) | <0.001 | 1.76 (1.34~2.31) | <0.001 | 1.61 (1.21~2.14) | 0.001 |
| Q4 | 193 (11.2) | 2.05 (1.6~2.63) | <0.001 | 2.55 (1.97~3.29) | <0.001 | 2.61 (2.02~3.38) | <0.001 | 1.89 (1.42~2.52) | <0.001 | 1.67 (1.23~2.27) | 0.001 |
| Trend test | 589 (8.6) | 1.25 (1.16~1.35) | <0.001 | 1.34 (1.24~1.44) | <0.001 | 1.35 (1.25~1.45) | <0.001 | 1.21 (1.11~1.32) | <0.001 | 1.16 (1.06~1.28) | 0.002 |
| TyG-WC |  |  |  |  |  |  |  |  |  |  |  |
| Q1 | 89 (5.2) | 1(Ref) |  |  |  |  |  |  |  |  |  |
| Q2 | 128 (7.5) | 1.44 (1.1~1.88) | 0.009 | 1.48 (1.13~1.94) | 0.005 | 1.48 (1.13~1.94) | 0.005 | 1.36 (1.04~1.79) | 0.027 | 1.26 (0.95~1.67) | 0.106 |
| Q3 | 167 (9.7) | 1.89 (1.46~2.45) | <0.001 | 1.96 (1.51~2.54) | <0.001 | 1.97 (1.52~2.55) | <0.001 | 1.65 (1.26~2.16) | <0.001 | 1.47 (1.11~1.96) | 0.008 |
| Q4 | 205 (11.9) | 2.35 (1.83~3.01) | <0.001 | 2.46 (1.91~3.15) | <0.001 | 2.48 (1.92~3.19) | <0.001 | 1.78 (1.34~2.36) | <0.001 | 1.55 (1.14~2.1) | 0.005 |
| Trend test | 589 (8.6) | 1.32 (1.22~1.42) | <0.001 | 1.33 (1.24~1.44) | <0.001 | 1.34 (1.24~1.44) | <0.001 | 1.2 (1.1~1.31) | <0.001 | 1.15 (1.04~1.26) | 0.005 |
| TyG-WHtR |  |  |  |  |  |  |  |  |  |  |  |
| Q1 | 91 (5.3) | 1(Ref) |  |  |  |  |  |  |  |  |  |
| Q2 | 120 (7) | 1.32 (1~1.73) | 0.048 | 1.41 (1.08~1.86) | 0.013 | 1.43 (1.09~1.88) | 0.011 | 1.32 (1~1.74) | 0.051 | 1.23 (0.92~1.63) | 0.158 |
| Q3 | 175 (10.2) | 1.9 (1.48~2.45) | <0.001 | 2.04 (1.58~2.64) | <0.001 | 2.06 (1.59~2.66) | <0.001 | 1.71 (1.3~2.24) | <0.001 | 1.53 (1.15~2.03) | 0.003 |
| Q4 | 203 (11.8) | 2.27 (1.78~2.91) | <0.001 | 2.5 (1.93~3.24) | <0.001 | 2.54 (1.96~3.29) | <0.001 | 1.82 (1.36~2.43) | <0.001 | 1.6 (1.17~2.19) | 0.003 |
| Trend test | 589 (8.6) | 1.32 (1.22~1.42) | <0.001 | 1.36 (1.25~1.47) | <0.001 | 1.36 (1.26~1.47) | <0.001 | 1.22 (1.11~1.33) | <0.001 | 1.17 (1.06~1.29) | 0.002 |
| TyG-BRI |  |  |  |  |  |  |  |  |  |  |  |
| Q1 | 92 (5.4) | 1(Ref) |  |  |  |  |  |  |  |  |  |
| Q2 | 123 (7.2) | 1.31 (1~1.71) | 0.051 | 1.4 (1.07~1.83) | 0.015 | 1.41 (1.08~1.85) | 0.013 | 1.3 (0.99~1.71) | 0.06 | 1.23 (0.93~1.62) | 0.152 |
| Q3 | 164 (9.6) | 1.76 (1.36~2.27) | <0.001 | 1.9 (1.47~2.47) | <0.001 | 1.93 (1.49~2.5) | <0.001 | 1.61 (1.23~2.1) | 0.001 | 1.46 (1.1~1.93) | 0.008 |
| Q4 | 210 (12.2) | 2.31 (1.81~2.95) | <0.001 | 2.57 (1.99~3.33) | <0.001 | 2.62 (2.02~3.4) | <0.001 | 1.92 (1.45~2.54) | <0.001 | 1.71 (1.27~2.3) | <0.001 |
| Trend test | 589 (8.6) | 1.32 (1.23~1.43) | <0.001 | 1.37 (1.26~1.48) | <0.001 | 1.37 (1.27~1.49) | <0.001 | 1.24 (1.13~1.35) | <0.001 | 1.19 (1.09~1.31) | <0.001 |
| TyG-WWI |  |  |  |  |  |  |  |  |  |  |  |
| Q1 | 84 (4.9) | 1(Ref) |  |  |  |  |  |  |  |  |  |
| Q2 | 149 (8.7) | 1.83 (1.4~2.39) | <0.001 | 1.81 (1.39~2.37) | <0.001 | 1.81 (1.38~2.37) | <0.001 | 1.64 (1.25~2.15) | <0.001 | 1.54 (1.17~2.03) | 0.002 |
| Q3 | 162 (9.4) | 1.97 (1.52~2.57) | <0.001 | 2 (1.53~2.61) | <0.001 | 2 (1.53~2.62) | <0.001 | 1.63 (1.24~2.16) | 0.001 | 1.46 (1.09~1.94) | 0.01 |
| Q4 | 194 (11.3) | 2.45 (1.9~3.17) | <0.001 | 2.39 (1.82~3.13) | <0.001 | 2.39 (1.82~3.13) | <0.001 | 1.65 (1.22~2.23) | 0.001 | 1.47 (1.07~2.02) | 0.016 |
| Trend test | 589 (8.6) | 1.29 (1.2~1.39) | <0.001 | 1.28 (1.18~1.39) | <0.001 | 1.28 (1.18~1.39) | <0.001 | 1.13 (1.04~1.24) | 0.006 | 1.09 (0.99~1.2) | 0.068 |
| TyG-CVAI |  |  |  |  |  |  |  |  |  |  |  |
| Q1 | 83 (4.8) | 1(Ref) |  |  |  |  |  |  |  |  |  |
| Q2 | 118 (6.9) | 1.42 (1.07~1.88) | 0.015 | 1.46 (1.1~1.93) | 0.009 | 1.47 (1.11~1.96) | 0.008 | 1.37 (1.03~1.82) | 0.031 | 1.3 (0.96~1.74) | 0.085 |
| Q3 | 169 (9.8) | 2.07 (1.59~2.69) | <0.001 | 2.08 (1.59~2.73) | <0.001 | 2.11 (1.61~2.77) | <0.001 | 1.79 (1.35~2.38) | <0.001 | 1.63 (1.21~2.2) | 0.001 |
| Q4 | 219 (12.8) | 2.78 (2.16~3.58) | <0.001 | 2.62 (2.02~3.4) | <0.001 | 2.67 (2.05~3.47) | <0.001 | 1.96 (1.46~2.62) | <0.001 | 1.78 (1.29~2.46) | 0.001 |
| Trend test | 589 (8.6) | 1.41 (1.3~1.52) | <0.001 | 1.37 (1.27~1.48) | <0.001 | 1.38 (1.27~1.49) | <0.001 | 1.24 (1.13~1.36) | <0.001 | 1.2 (1.09~1.33) | <0.001 |
| TyG-ABSI |  |  |  |  |  |  |  |  |  |  |  |
| Q1 | 94 (5.5) | 1(Ref) |  |  |  |  |  |  |  |  |  |
| Q2 | 142 (8.3) | 1.54 (1.19~2) | 0.001 | 1.46 (1.13~1.9) | 0.004 | 1.45 (1.12~1.89) | 0.005 | 1.32 (1.01~1.72) | 0.039 | 1.21 (0.93~1.59) | 0.155 |
| Q3 | 160 (9.3) | 1.78 (1.38~2.3) | <0.001 | 1.64 (1.27~2.12) | <0.001 | 1.64 (1.27~2.12) | <0.001 | 1.35 (1.03~1.76) | 0.028 | 1.21 (0.92~1.58) | 0.176 |
| Q4 | 193 (11.2) | 2.22 (1.73~2.84) | <0.001 | 1.96 (1.52~2.52) | <0.001 | 1.95 (1.51~2.51) | <0.001 | 1.37 (1.03~1.81) | 0.029 | 1.22 (0.91~1.63) | 0.178 |
| Trend test | 589 (8.6) | 1.28 (1.18~1.37) | <0.001 | 1.23 (1.14~1.32) | <0.001 | 1.22 (1.13~1.32) | <0.001 | 1.09 (1~1.19) | 0.053 | 1.05 (0.96~1.15) | 0.265 |
| CTI |  |  |  |  |  |  |  |  |  |  |  |
| Q1 | 80 (4.7) | 1(Ref) |  |  |  |  |  |  |  |  |  |
| Q2 | 140 (8.2) | 1.8 (1.37~2.37) | <0.001 | 1.72 (1.31~2.27) | <0.001 | 1.72 (1.31~2.27) | <0.001 | 1.63 (1.23~2.15) | 0.001 | 1.54 (1.16~2.05) | 0.003 |
| Q3 | 180 (10.5) | 2.4 (1.84~3.12) | <0.001 | 2.3 (1.77~2.99) | <0.001 | 2.3 (1.76~2.99) | <0.001 | 1.95 (1.48~2.57) | <0.001 | 1.75 (1.31~2.33) | <0.001 |
| Q4 | 189 (11) | 2.55 (1.97~3.32) | <0.001 | 2.45 (1.88~3.18) | <0.001 | 2.43 (1.87~3.17) | <0.001 | 1.79 (1.33~2.4) | <0.001 | 1.62 (1.18~2.22) | 0.003 |
| Trend test | 589 (8.6) | 1.32 (1.23~1.42) | <0.001 | 1.31 (1.22~1.41) | <0.001 | 1.31 (1.21~1.41) | <0.001 | 1.18 (1.08~1.29) | <0.001 | 1.14 (1.04~1.26) | 0.006 |
| CKM syndrome, cardiovascular kidney-metabolic syndrome; TyG, triglyceride-glucose index; TyG-BMI, triglyceride glucose-body mass index; TyG-WC, triglyceride glucose-waist circumference; TyG-WHtR, triglyceride glucose-waist height ratio index; TyG-BRI, triglyceride glucose-body roundness index; TyG-WWI, triglyceride glucose-weight-adjusted waist index; TyG-CVAI, triglyceride glucose-Chinese visceral adiposity index; TyG-ABSI, triglyceride glucose-a body shape index; CTI, C-reactive protein-triglyceride-glucose index; SBP, systolic blood pressure; DBP, diastolic blood pressure; HDL-C, high-density lipoprotein cholesterol; LDL-C, low-density lipoprotein cholesterol; eGFR, estimated glomerular filtration rate; UA, uric acid; Cr, creatinine; BUN, blood urea nitrogen; Hb, hemoglobin; MCV, mean corpuscular volume; PCV, packed cell volume; WBC, white blood cell count Model 1 adjusted for age, gender Model 2 adjusted for Model 1+marital status, education level, Hu kou, smoking status, drinking status Model 3 adjusted for Model 2+hypertension, diabetes, dyslipidemia, kidney disease Model 4 adjusted for Model 3+stages of CKM syndrome, SBP, DBP, HDL-C, LDL-C, eGFR, UA, Cr, BUN, Hb, MCV, PCV, platelet count, WBC  The hazard ratios (HRs) presented in the table were derived from cox proportional hazards regression models | | | | | | | | | | | |

| **Table S6** Associations between TyG-related indices and CHD risk in CKM syndrome stage 0-3 | | | | | | | | | | | |
| --- | --- | --- | --- | --- | --- | --- | --- | --- | --- | --- | --- |
|  | Case | Crude |  | Model 1 |  | Model 2 |  | Model 3 |  | Model 4 |  |
|  |  | HR (95%CI) | P | HR (95%CI) | P | HR (95%CI) | P | HR (95%CI) | P | HR (95%CI) | P |
| TyG |  |  |  |  |  |  |  |  |  |  |  |
| Q1 | 275 (16) | 1(Ref) |  |  |  |  |  |  |  |  |  |
| Q2 | 323 (18.8) | 1.19 (1.01~1.39) | 0.037 | 1.17 (1~1.38) | 0.054 | 1.16 (0.98~1.36) | 0.078 | 1.11 (0.94~1.3) | 0.218 | 1.09 (0.92~1.28) | 0.329 |
| Q3 | 339 (19.7) | 1.27 (1.08~1.49) | 0.004 | 1.2 (1.03~1.41) | 0.023 | 1.18 (1.01~1.39) | 0.04 | 1.07 (0.91~1.27) | 0.411 | 1.04 (0.87~1.24) | 0.65 |
| Q4 | 362 (21.1) | 1.37 (1.17~1.6) | <0.001 | 1.32 (1.13~1.55) | <0.001 | 1.3 (1.11~1.52) | 0.001 | 1.06 (0.87~1.28) | 0.572 | 1.05 (0.86~1.29) | 0.649 |
| Trend test | 1299 (18.9) | 1.1 (1.05~1.16) | <0.001 | 1.09 (1.04~1.14) | 0.001 | 1.08 (1.03~1.14) | 0.002 | 1.01 (0.95~1.08) | 0.64 | 1.01 (0.95~1.08) | 0.748 |
| TyG-BMI |  |  |  |  |  |  |  |  |  |  |  |
| Q1 | 255 (14.9) | 1(Ref) |  |  |  |  |  |  |  |  |  |
| Q2 | 304 (17.7) | 1.18 (1~1.39) | 0.052 | 1.21 (1.03~1.44) | 0.023 | 1.21 (1.02~1.43) | 0.026 | 1.17 (0.99~1.38) | 0.069 | 1.16 (0.97~1.38) | 0.097 |
| Q3 | 323 (18.8) | 1.27 (1.07~1.49) | 0.005 | 1.31 (1.11~1.55) | 0.001 | 1.29 (1.09~1.52) | 0.003 | 1.18 (0.99~1.41) | 0.06 | 1.2  (1~1.45) | 0.053 |
| Q4 | 417 (24.3) | 1.67 (1.43~1.95) | <0.001 | 1.74 (1.48~2.04) | <0.001 | 1.7 (1.44~1.99) | <0.001 | 1.47 (1.23~1.76) | <0.001 | 1.51 (1.24~1.84) | <0.001 |
| Trend test | 1299 (18.9) | 1.18 (1.12~1.24) | <0.001 | 1.19 (1.13~1.26) | <0.001 | 1.18 (1.12~1.25) | <0.001 | 1.13 (1.06~1.19) | <0.001 | 1.14 (1.07~1.21) | <0.001 |
| TyG-WC |  |  |  |  |  |  |  |  |  |  |  |
| Q1 | 237 (13.8) | 1(Ref) |  |  |  |  |  |  |  |  |  |
| Q2 | 315 (18.4) | 1.36 (1.15~1.61) | <0.001 | 1.35 (1.14~1.6) | 0.001 | 1.34 (1.13~1.59) | 0.001 | 1.29 (1.09~1.53) | 0.003 | 1.31 (1.09~1.56) | 0.003 |
| Q3 | 330 (19.2) | 1.44 (1.22~1.7) | <0.001 | 1.4 (1.19~1.66) | <0.001 | 1.37 (1.16~1.62) | <0.001 | 1.28 (1.07~1.52) | 0.006 | 1.3 (1.08~1.57) | 0.006 |
| Q4 | 417 (24.3) | 1.87 (1.59~2.19) | <0.001 | 1.81 (1.54~2.13) | <0.001 | 1.77 (1.51~2.08) | <0.001 | 1.55 (1.29~1.86) | <0.001 | 1.61 (1.32~1.97) | <0.001 |
| Trend test | 1299 (18.9) | 1.21 (1.15~1.27) | <0.001 | 1.2 (1.14~1.26) | <0.001 | 1.19 (1.13~1.25) | <0.001 | 1.14 (1.07~1.2) | <0.001 | 1.15 (1.08~1.22) | <0.001 |
| TyG-WHtR |  |  |  |  |  |  |  |  |  |  |  |
| Q1 | 242 (14.1) | 1(Ref) |  |  |  |  |  |  |  |  |  |
| Q2 | 305 (17.8) | 1.29 (1.09~1.53) | 0.003 | 1.26 (1.06~1.49) | 0.009 | 1.24 (1.05~1.47) | 0.013 | 1.2 (1.01~1.42) | 0.04 | 1.18 (0.99~1.41) | 0.066 |
| Q3 | 335 (19.5) | 1.41 (1.19~1.66) | <0.001 | 1.31 (1.11~1.55) | 0.001 | 1.29 (1.09~1.53) | 0.003 | 1.18 (0.99~1.41) | 0.066 | 1.17 (0.97~1.41) | 0.1 |
| Q4 | 417 (24.3) | 1.84 (1.57~2.15) | <0.001 | 1.63 (1.38~1.92) | <0.001 | 1.59 (1.35~1.88) | <0.001 | 1.36 (1.12~1.64) | 0.001 | 1.36 (1.11~1.67) | 0.003 |
| Trend test | 1299 (18.9) | 1.21 (1.15~1.27) | <0.001 | 1.16 (1.1~1.22) | <0.001 | 1.15 (1.1~1.22) | <0.001 | 1.09 (1.03~1.16) | 0.004 | 1.09 (1.02~1.17) | 0.007 |
| TyG-BRI |  |  |  |  |  |  |  |  |  |  |  |
| Q1 | 246 (14.3) | 1(Ref) |  |  |  |  |  |  |  |  |  |
| Q2 | 297 (17.3) | 1.22 (1.03~1.44) | 0.022 | 1.18 (1~1.4) | 0.052 | 1.17 (0.99~1.39) | 0.068 | 1.12 (0.95~1.33) | 0.185 | 1.11 (0.93~1.32) | 0.262 |
| Q3 | 329 (19.2) | 1.34 (1.14~1.59) | <0.001 | 1.26 (1.07~1.49) | 0.007 | 1.24 (1.04~1.46) | 0.014 | 1.13 (0.95~1.34) | 0.182 | 1.11 (0.92~1.33) | 0.274 |
| Q4 | 427 (24.9) | 1.85 (1.58~2.16) | <0.001 | 1.62 (1.37~1.91) | <0.001 | 1.59 (1.35~1.88) | <0.001 | 1.36 (1.13~1.62) | 0.001 | 1.35 (1.11~1.63) | 0.003 |
| Trend test | 1299 (18.9) | 1.22 (1.16~1.28) | <0.001 | 1.17 (1.11~1.23) | <0.001 | 1.16 (1.1~1.22) | <0.001 | 1.1 (1.04~1.16) | 0.001 | 1.1 (1.03~1.17) | 0.004 |
| TyG-WWI |  |  |  |  |  |  |  |  |  |  |  |
| Q1 | 251 (14.6) | 1(Ref) |  |  |  |  |  |  |  |  |  |
| Q2 | 299 (17.4) | 1.22 (1.03~1.45) | 0.019 | 1.15 (0.97~1.36) | 0.114 | 1.15 (0.97~1.36) | 0.108 | 1.1 (0.93~1.3) | 0.28 | 1.09 (0.91~1.29) | 0.353 |
| Q3 | 364 (21.2) | 1.52 (1.3~1.79) | <0.001 | 1.35 (1.15~1.6) | <0.001 | 1.35 (1.14~1.59) | <0.001 | 1.21 (1.02~1.44) | 0.027 | 1.19 (0.99~1.42) | 0.057 |
| Q4 | 385 (22.4) | 1.65 (1.41~1.93) | <0.001 | 1.33 (1.13~1.58) | 0.001 | 1.33 (1.12~1.58) | 0.001 | 1.1 (0.91~1.33) | 0.331 | 1.08 (0.88~1.32) | 0.467 |
| Trend test | 1299 (18.9) | 1.18 (1.13~1.24) | <0.001 | 1.1 (1.05~1.16) | <0.001 | 1.1 (1.05~1.16) | <0.001 | 1.04 (0.98~1.1) | 0.241 | 1.03 (0.97~1.1) | 0.371 |
| TyG-CVAI |  |  |  |  |  |  |  |  |  |  |  |
| Q1 | 218 (12.7) | 1(Ref) |  |  |  |  |  |  |  |  |  |
| Q2 | 310 (18.1) | 1.46 (1.23~1.73) | <0.001 | 1.35 (1.13~1.6) | 0.001 | 1.33 (1.12~1.59) | 0.001 | 1.29 (1.08~1.54) | 0.005 | 1.3 (1.08~1.57) | 0.005 |
| Q3 | 351 (20.4) | 1.69 (1.42~2) | <0.001 | 1.5 (1.26~1.78) | <0.001 | 1.46 (1.23~1.74) | <0.001 | 1.36 (1.13~1.63) | 0.001 | 1.41 (1.16~1.73) | 0.001 |
| Q4 | 420 (24.5) | 2.1 (1.79~2.48) | <0.001 | 1.81 (1.53~2.15) | <0.001 | 1.77 (1.49~2.09) | <0.001 | 1.53 (1.26~1.86) | <0.001 | 1.65 (1.32~2.05) | <0.001 |
| Trend test | 1299 (18.9) | 1.26 (1.2~1.32) | <0.001 | 1.2 (1.14~1.27) | <0.001 | 1.19 (1.13~1.25) | <0.001 | 1.14 (1.07~1.21) | <0.001 | 1.16 (1.09~1.25) | <0.001 |
| TyG-ABSI |  |  |  |  |  |  |  |  |  |  |  |
| Q1 | 264 (15.4) | 1(Ref) |  |  |  |  |  |  |  |  |  |
| Q2 | 309 (18) | 1.21 (1.03~1.43) | 0.023 | 1.15 (0.97~1.35) | 0.102 | 1.15 (0.97~1.35) | 0.1 | 1.1 (0.94~1.31) | 0.24 | 1.09 (0.92~1.29) | 0.34 |
| Q3 | 356 (20.7) | 1.44 (1.23~1.68) | <0.001 | 1.3 (1.1~1.52) | 0.002 | 1.29 (1.1~1.51) | 0.002 | 1.17 (0.99~1.39) | 0.06 | 1.15 (0.97~1.37) | 0.111 |
| Q4 | 370 (21.5) | 1.52 (1.3~1.78) | <0.001 | 1.28 (1.08~1.5) | 0.003 | 1.27 (1.08~1.49) | 0.005 | 1.07 (0.89~1.28) | 0.481 | 1.05 (0.86~1.27) | 0.645 |
| Trend test | 1299 (18.9) | 1.15 (1.09~1.21) | <0.001 | 1.09 (1.03~1.14) | 0.001 | 1.08 (1.03~1.14) | 0.002 | 1.02 (0.97~1.09) | 0.413 | 1.02 (0.96~1.08) | 0.563 |
| CTI |  |  |  |  |  |  |  |  |  |  |  |
| Q1 | 258 (15) | 1(Ref) |  |  |  |  |  |  |  |  |  |
| Q2 | 322 (18.8) | 1.29 (1.09~1.52) | 0.002 | 1.24 (1.05~1.46) | 0.01 | 1.23 (1.04~1.45) | 0.015 | 1.2 (1.01~1.41) | 0.033 | 1.19 (1.01~1.41) | 0.042 |
| Q3 | 335 (19.5) | 1.37 (1.17~1.62) | <0.001 | 1.3 (1.11~1.53) | 0.002 | 1.28 (1.09~1.51) | 0.003 | 1.19 (1~1.41) | 0.047 | 1.18 (0.99~1.42) | 0.067 |
| Q4 | 384 (22.4) | 1.62 (1.38~1.89) | <0.001 | 1.52 (1.29~1.78) | <0.001 | 1.48 (1.26~1.74) | <0.001 | 1.28 (1.07~1.54) | 0.007 | 1.3 (1.07~1.59) | 0.009 |
| Trend test | 1299 (18.9) | 1.16 (1.1~1.22) | <0.001 | 1.14 (1.08~1.19) | <0.001 | 1.13 (1.07~1.18) | <0.001 | 1.08 (1.01~1.14) | 0.014 | 1.08 (1.01~1.15) | 0.017 |
| CHD, coronary heart disease; CKM syndrome, cardiovascular kidney-metabolic syndrome; TyG, triglyceride-glucose index; TyG-BMI, triglyceride glucose-body mass index; TyG-WC, triglyceride glucose-waist circumference; TyG-WHtR, triglyceride glucose-waist height ratio index; TyG-BRI, triglyceride glucose-body roundness index; TyG-WWI, triglyceride glucose-weight-adjusted waist index; TyG-CVAI, triglyceride glucose-Chinese visceral adiposity index; TyG-ABSI, triglyceride glucose-a body shape index; CTI, C-reactive protein-triglyceride-glucose index; SBP, systolic blood pressure; DBP, diastolic blood pressure; HDL-C, high-density lipoprotein cholesterol; LDL-C, low-density lipoprotein cholesterol; eGFR, estimated glomerular filtration rate; UA, uric acid; Cr, creatinine; BUN, blood urea nitrogen; Hb, hemoglobin; MCV, mean corpuscular volume; PCV, packed cell volume; WBC, white blood cell count Model 1 adjusted for age, gender Model 2 adjusted for Model 1+marital status, education level, Hu kou, smoking status, drinking status Model 3 adjusted for Model 2+hypertension, diabetes, dyslipidemia, kidney disease Model 4 adjusted for Model 3+stages of CKM syndrome, SBP, DBP, HDL-C, LDL-C, eGFR, UA, Cr, BUN, Hb, MCV, PCV, platelet count, WBC  The hazard ratios (HRs) presented in the table were derived from cox proportional hazards regression models | | | | | | | | | | | |

| **Table S7** Incremental predictive value of TyG-related indices for stroke in CKM syndrome stage 0-3 | | | | |
| --- | --- | --- | --- | --- |
| Model | NRI (95%CI) | P | IDI (95%CI) | P |
| Basic model | Ref | Ref | Ref | Ref |
| +TyG | 0.0327 (-0.0517-0.1171) | 0.448 | 0.0002 (-0.0003-0.0007) | 0.480 |
| +TyG-BMI | 0.0981 (0.0137-0.1825) | 0.023 | 0.0013 (0.0001-0.0025) | 0.036 |
| +TyG-WC | 0.1222 (0.0378-0.2065) | 0.005 | 0.0016 (0.0002-0.0029) | 0.020 |
| +TyG-WHtR | 0.1337 (0.0493-0.2180) | 0.002 | 0.0018 (0.0004-0.0032) | 0.011 |
| +TyG-BRI | 0.1282 (0.0438-0.2126) | 0.003 | 0.0019 (0.0005-0.0033) | 0.008 |
| +TyG-WWI | 0.0656 (-0.0188-0.1501) | 0.128 | 0.0009 (-0.0001-0.0018) | 0.079 |
| +TyG-CVAI | 0.1516 (0.0673-0.2359) | <0.001 | 0.0019 (0.0005-0.0033) | 0.008 |
| +TyG-ABSI | 0.0063 (-0.0781-0.0907) | 0.884 | 0.0003 (-0.0003-0.0010) | 0.271 |
| +CTI | 0.0869 (0.0026-0.1713) | 0.043 | 0.0008 (-0.0002-0.0018) | 0.127 |
| The basic model included age, gender, marital status, education level, Hu kou, smoking status, drinking status, hypertension, diabetes, dyslipidemia, kidney disease, SBP, DBP, HDL-C, LDL-C, eGFR, UA, Cr, BUN, Hb, MCV, PCV, platelet count, WBC, stages of CKM syndrome  NRI, net reclassification improvement; Ref, reference; IDI, integrated discrimination improvement; CI, confidence interval; TyG, triglyceride-glucose index; TyG-BMI, triglyceride glucose-body mass index; TyG-WC, triglyceride glucose-waist circumference; TyG-WHtR, triglyceride glucose-waist height ratio index; TyG-BRI, triglyceride glucose-body roundness index; TyG-WWI, triglyceride glucose-weight-adjusted waist index; TyG-CVAI, triglyceride glucose-Chinese visceral adiposity index; TyG-ABSI, triglyceride glucose-a body shape index; CTI, C-reactive protein-triglyceride-glucose index; SBP, systolic blood pressure; DBP, diastolic blood pressure; HDL-C, high-density lipoprotein cholesterol; LDL-C, low-density lipoprotein cholesterol; eGFR, estimated glomerular filtration rate; UA, uric acid; Cr, creatinine; BUN, blood urea nitrogen; Hb, hemoglobin; MCV, mean corpuscular volume; PCV, packed cell volume; WBC, white blood cell count | | | | |

| **Table S8** Incremental predictive value of TyG-related indices for CHD in CKM syndrome stage 0-3 | | | | |
| --- | --- | --- | --- | --- |
| Model | NRI (95%CI) | P | IDI (95%CI) | P |
| Basic model | Ref | Ref | Ref | Ref |
| +TyG | 0.0122 (-0.0482-0.0725) | 0.693 | 0.0000 (-0.0001-0.0001) | 0.593 |
| +TyG-BMI | 0.1095 (0.0491-0.1698) | <0.001 | 0.0049 (0.0030-0.0067) | <0.001 |
| +TyG-WC | 0.0946 (0.0342-0.1549) | 0.002 | 0.0041 (0.0025-0.0057) | <0.001 |
| +TyG-WHtR | 0.0535 (-0.0069-0.1139) | 0.082 | 0.0019 (0.0008-0.0030) | <0.001 |
| +TyG-BRI | 0.0831 (0.0227-0.1434) | 0.007 | 0.0023 (0.0011-0.0036) | <0.001 |
| +TyG-WWI | -0.0187 (-0.0791-0.042) | 0.544 | 0.0000 (-0.0001-0.0001) | 0.641 |
| +TyG-CVAI | 0.0728 (0.0125-0.1332) | 0.018 | 0.0044 (0.0028-0.0060) | <0.001 |
| +TyG-ABSI | -0.0194 (-0.0798-0.0410) | 0.529 | 0.0000 (0.0000-0.0000) | 0.866 |
| +CTI | 0.0524 (-0.0079-0.1128) | 0.088 | 0.0003 (-0.0001-0.0008) | 0.096 |
| The basic model included age, gender, marital status, education level, Hu kou, smoking status, drinking status, hypertension, diabetes, dyslipidemia, kidney disease, SBP, DBP, HDL-C, LDL-C, eGFR, UA, Cr, BUN, Hb, MCV, PCV, platelet count, WBC, stages of CKM syndrome  CHD, coronary heart disease; NRI, net reclassification improvement; Ref, reference; IDI, integrated discrimination improvement; CI, confidence interval; TyG, triglyceride-glucose index; TyG-BMI, triglyceride glucose-body mass index; TyG-WC, triglyceride glucose-waist circumference; TyG-WHtR, triglyceride glucose-waist height ratio index; TyG-BRI, triglyceride glucose-body roundness index; TyG-WWI, triglyceride glucose-weight-adjusted waist index; TyG-CVAI, triglyceride glucose-Chinese visceral adiposity index; TyG-ABSI, triglyceride glucose-a body shape index; CTI, C-reactive protein-triglyceride-glucose index; SBP, systolic blood pressure; DBP, diastolic blood pressure; HDL-C, high-density lipoprotein cholesterol; LDL-C, low-density lipoprotein cholesterol; eGFR, estimated glomerular filtration rate; UA, uric acid; Cr, creatinine; BUN, blood urea nitrogen; Hb, hemoglobin; MCV, mean corpuscular volume; PCV, packed cell volume; WBC, white blood cell count | | | | |

| **Table S9** Associations between TyG-related indices and CMM risk in CKM syndrome stage 0-3 without diabetes at baseline | | | | | | | | | | | |
| --- | --- | --- | --- | --- | --- | --- | --- | --- | --- | --- | --- |
|  | Case | Crude |  | Model 1 |  | Model 2 |  | Model 3 |  | Model 4 |  |
|  |  | HR (95%CI) | P | HR (95%CI) | P | HR (95%CI) | P | HR (95%CI) | P | HR (95%CI) | P |
| TyG |  |  |  |  |  |  |  |  |  |  |  |
| Q1 | 71 (4.4) | 1(Ref) |  |  |  |  |  |  |  |  |  |
| Q2 | 113 (7) | 1.59 (1.18~2.14) | 0.002 | 1.6 (1.19~2.16) | 0.002 | 1.59 (1.18~2.15) | 0.002 | 1.49 (1.1~2.01) | 0.009 | 1.37 (1.01~1.86) | 0.04 |
| Q3 | 140 (8.7) | 2  (1.5~2.66) | <0.001 | 1.98 (1.49~2.63) | <0.001 | 1.97 (1.48~2.62) | <0.001 | 1.64 (1.22~2.21) | 0.001 | 1.38 (1.01~1.88) | 0.041 |
| Q4 | 192 (11.9) | 2.82 (2.15~3.71) | <0.001 | 2.85 (2.17~3.74) | <0.001 | 2.82 (2.15~3.71) | <0.001 | 1.96 (1.43~2.7) | <0.001 | 1.7 (1.22~2.38) | 0.002 |
| Trend test | 516 (8) | 1.39 (1.28~1.5) | <0.001 | 1.39 (1.28~1.51) | <0.001 | 1.38 (1.28~1.5) | <0.001 | 1.22 (1.11~1.35) | <0.001 | 1.17 (1.05~1.3) | 0.004 |
| TyG-BMI |  |  |  |  |  |  |  |  |  |  |  |
| Q1 | 69 (4.3) | 1(Ref) |  |  |  |  |  |  |  |  |  |
| Q2 | 100 (6.2) | 1.42 (1.04~1.92) | 0.026 | 1.53 (1.12~2.08) | 0.007 | 1.54 (1.13~2.1) | 0.006 | 1.41 (1.03~1.92) | 0.03 | 1.31 (0.95~1.8) | 0.094 |
| Q3 | 124 (7.7) | 1.77 (1.32~2.38) | <0.001 | 2.01 (1.49~2.71) | <0.001 | 2.02 (1.5~2.73) | <0.001 | 1.65 (1.21~2.24) | 0.002 | 1.47 (1.07~2.04) | 0.019 |
| Q4 | 223 (13.8) | 3.24 (2.47~4.24) | <0.001 | 3.77 (2.86~4.98) | <0.001 | 3.85 (2.92~5.1) | <0.001 | 2.8 (2.06~3.8) | <0.001 | 2.43 (1.75~3.38) | <0.001 |
| Trend test | 516 (8) | 1.49 (1.37~1.62) | <0.001 | 1.56 (1.43~1.7) | <0.001 | 1.57 (1.44~1.71) | <0.001 | 1.41 (1.28~1.55) | <0.001 | 1.35 (1.22~1.5) | <0.001 |
| TyG-WC |  |  |  |  |  |  |  |  |  |  |  |
| Q1 | 59 (3.7) | 1(Ref) |  |  |  |  |  |  |  |  |  |
| Q2 | 93 (5.8) | 1.57 (1.13~2.18) | 0.007 | 1.6 (1.15~2.21) | 0.005 | 1.6 (1.15~2.21) | 0.005 | 1.47 (1.06~2.04) | 0.022 | 1.34 (0.96~1.88) | 0.087 |
| Q3 | 136 (8.4) | 2.33 (1.71~3.16) | <0.001 | 2.35 (1.73~3.19) | <0.001 | 2.35 (1.73~3.2) | <0.001 | 1.98 (1.44~2.72) | <0.001 | 1.77 (1.27~2.47) | 0.001 |
| Q4 | 228 (14.1) | 4.06 (3.05~5.41) | <0.001 | 4.14 (3.1~5.51) | <0.001 | 4.15 (3.11~5.54) | <0.001 | 3.11 (2.26~4.28) | <0.001 | 2.74 (1.95~3.86) | <0.001 |
| Trend test | 516 (8) | 1.6 (1.47~1.74) | <0.001 | 1.61 (1.48~1.75) | <0.001 | 1.61 (1.48~1.75) | <0.001 | 1.46 (1.32~1.61) | <0.001 | 1.41 (1.27~1.57) | <0.001 |
| TyG-WHtR |  |  |  |  |  |  |  |  |  |  |  |
| Q1 | 58 (3.6) | 1(Ref) |  |  |  |  |  |  |  |  |  |
| Q2 | 98 (6.1) | 1.7 (1.23~2.35) | 0.001 | 1.78 (1.29~2.47) | 0.001 | 1.79 (1.29~2.48) | <0.001 | 1.64 (1.18~2.28) | 0.003 | 1.49 (1.06~2.09) | 0.02 |
| Q3 | 134 (8.3) | 2.29 (1.68~3.11) | <0.001 | 2.38 (1.74~3.25) | <0.001 | 2.38 (1.74~3.26) | <0.001 | 1.97 (1.43~2.72) | <0.001 | 1.75 (1.25~2.45) | 0.001 |
| Q4 | 226 (14) | 4.09 (3.06~5.45) | <0.001 | 4.32 (3.2~5.83) | <0.001 | 4.36 (3.23~5.89) | <0.001 | 3.19 (2.3~4.43) | <0.001 | 2.77 (1.95~3.95) | <0.001 |
| Trend test | 516 (8) | 1.58 (1.45~1.72) | <0.001 | 1.6 (1.47~1.75) | <0.001 | 1.61 (1.47~1.76) | <0.001 | 1.44 (1.31~1.59) | <0.001 | 1.39 (1.25~1.54) | <0.001 |
| TyG-BRI |  |  |  |  |  |  |  |  |  |  |  |
| Q1 | 64 (4) | 1(Ref) |  |  |  |  |  |  |  |  |  |
| Q2 | 98 (6.1) | 1.51 (1.1~2.07) | 0.01 | 1.58 (1.15~2.17) | 0.005 | 1.58 (1.15~2.17) | 0.004 | 1.43 (1.04~1.97) | 0.027 | 1.31 (0.95~1.81) | 0.101 |
| Q3 | 131 (8.1) | 2.01 (1.49~2.71) | <0.001 | 2.11 (1.56~2.86) | <0.001 | 2.11 (1.56~2.86) | <0.001 | 1.73 (1.27~2.36) | 0.001 | 1.53 (1.11~2.11) | 0.01 |
| Q4 | 223 (13.8) | 3.6 (2.73~4.76) | <0.001 | 3.8 (2.84~5.08) | <0.001 | 3.85 (2.87~5.16) | <0.001 | 2.77 (2.03~3.78) | <0.001 | 2.39 (1.72~3.31) | <0.001 |
| Trend test | 516 (8) | 1.54 (1.41~1.67) | <0.001 | 1.56 (1.42~1.7) | <0.001 | 1.56 (1.43~1.71) | <0.001 | 1.4 (1.27~1.54) | <0.001 | 1.34 (1.21~1.48) | <0.001 |
| TyG-WWI |  |  |  |  |  |  |  |  |  |  |  |
| Q1 | 66 (4.1) | 1(Ref) |  |  |  |  |  |  |  |  |  |
| Q2 | 97 (6) | 1.49 (1.09~2.03) | 0.013 | 1.47 (1.08~2.02) | 0.015 | 1.47 (1.08~2.02) | 0.015 | 1.33 (0.97~1.83) | 0.079 | 1.21 (0.88~1.67) | 0.244 |
| Q3 | 144 (8.9) | 2.22 (1.66~2.97) | <0.001 | 2.21 (1.65~2.98) | <0.001 | 2.23 (1.65~3) | <0.001 | 1.8 (1.32~2.45) | <0.001 | 1.57 (1.15~2.16) | 0.005 |
| Q4 | 209 (12.9) | 3.42 (2.59~4.51) | <0.001 | 3.31 (2.47~4.43) | <0.001 | 3.32 (2.48~4.45) | <0.001 | 2.36 (1.71~3.26) | <0.001 | 2.03 (1.45~2.83) | <0.001 |
| Trend test | 516 (8) | 1.51 (1.39~1.64) | <0.001 | 1.49 (1.37~1.63) | <0.001 | 1.49 (1.37~1.63) | <0.001 | 1.33 (1.21~1.47) | <0.001 | 1.27 (1.15~1.41) | <0.001 |
| TyG-CVAI |  |  |  |  |  |  |  |  |  |  |  |
| Q1 | 58 (3.6) | 1(Ref) |  |  |  |  |  |  |  |  |  |
| Q2 | 92 (5.7) | 1.57 (1.13~2.19) | 0.007 | 1.56 (1.12~2.17) | 0.009 | 1.56 (1.12~2.17) | 0.008 | 1.44 (1.03~2.02) | 0.031 | 1.32 (0.94~1.86) | 0.114 |
| Q3 | 143 (8.9) | 2.5 (1.84~3.39) | <0.001 | 2.42 (1.77~3.3) | <0.001 | 2.42 (1.77~3.31) | <0.001 | 2.04 (1.48~2.82) | <0.001 | 1.85 (1.31~2.61) | <0.001 |
| Q4 | 223 (13.8) | 4.12 (3.09~5.51) | <0.001 | 3.83 (2.85~5.15) | <0.001 | 3.84 (2.85~5.17) | <0.001 | 2.77 (2~3.85) | <0.001 | 2.49 (1.73~3.59) | <0.001 |
| Trend test | 516 (8) | 1.61 (1.48~1.75) | <0.001 | 1.57 (1.44~1.71) | <0.001 | 1.57 (1.44~1.71) | <0.001 | 1.4 (1.27~1.54) | <0.001 | 1.36 (1.22~1.52) | <0.001 |
| TyG-ABSI |  |  |  |  |  |  |  |  |  |  |  |
| Q1 | 66 (4.1) | 1(Ref) |  |  |  |  |  |  |  |  |  |
| Q2 | 116 (7.2) | 1.79 (1.32~2.42) | <0.001 | 1.73 (1.27~2.33) | <0.001 | 1.72 (1.27~2.33) | <0.001 | 1.56 (1.15~2.12) | 0.004 | 1.41 (1.03~1.91) | 0.031 |
| Q3 | 139 (8.6) | 2.18 (1.63~2.93) | <0.001 | 2.04 (1.52~2.74) | <0.001 | 2.04 (1.52~2.74) | <0.001 | 1.66 (1.23~2.26) | 0.001 | 1.47 (1.08~2) | 0.016 |
| Q4 | 195 (12.1) | 3.25 (2.46~4.3) | <0.001 | 2.89 (2.17~3.85) | <0.001 | 2.88 (2.16~3.84) | <0.001 | 2.05 (1.5~2.8) | <0.001 | 1.77 (1.28~2.44) | 0.001 |
| Trend test | 516 (8) | 1.43 (1.32~1.56) | <0.001 | 1.38 (1.27~1.5) | <0.001 | 1.38 (1.27~1.5) | <0.001 | 1.23 (1.12~1.35) | <0.001 | 1.18 (1.07~1.3) | 0.001 |
| CTI |  |  |  |  |  |  |  |  |  |  |  |
| Q1 | 57 (3.5) | 1(Ref) |  |  |  |  |  |  |  |  |  |
| Q2 | 112 (6.9) | 2.03 (1.48~2.8) | <0.001 | 1.96 (1.42~2.7) | <0.001 | 1.96 (1.42~2.69) | <0.001 | 1.86 (1.35~2.56) | <0.001 | 1.73 (1.25~2.4) | 0.001 |
| Q3 | 138 (8.5) | 2.57 (1.89~3.5) | <0.001 | 2.45 (1.8~3.34) | <0.001 | 2.44 (1.79~3.33) | <0.001 | 2.11 (1.53~2.9) | <0.001 | 1.85 (1.33~2.58) | <0.001 |
| Q4 | 209 (12.9) | 4.06 (3.03~5.44) | <0.001 | 3.87 (2.88~5.19) | <0.001 | 3.83 (2.85~5.14) | <0.001 | 2.92 (2.11~4.04) | <0.001 | 2.53 (1.79~3.58) | <0.001 |
| Trend test | 516 (8) | 1.53 (1.41~1.66) | <0.001 | 1.51 (1.39~1.64) | <0.001 | 1.5 (1.38~1.63) | <0.001 | 1.37 (1.24~1.5) | <0.001 | 1.31 (1.18~1.45) | <0.001 |
| CMM, cardiometabolic multimorbidity; CKM syndrome, cardiovascular kidney-metabolic syndrome; TyG, triglyceride-glucose index; TyG-BMI, triglyceride glucose-body mass index; TyG-WC, triglyceride glucose-waist circumference; TyG-WHtR, triglyceride glucose-waist height ratio index; TyG-BRI, triglyceride glucose-body roundness index; TyG-WWI, triglyceride glucose-weight-adjusted waist index; TyG-CVAI, triglyceride glucose-Chinese visceral adiposity index; TyG-ABSI, triglyceride glucose-a body shape index; CTI, C-reactive protein-triglyceride-glucose index; SBP, systolic blood pressure; DBP, diastolic blood pressure; HDL-C, high-density lipoprotein cholesterol; LDL-C, low-density lipoprotein cholesterol; eGFR, estimated glomerular filtration rate; UA, uric acid; Cr, creatinine; BUN, blood urea nitrogen; Hb, hemoglobin; MCV, mean corpuscular volume; PCV, packed cell volume; WBC, white blood cell count Model 1 adjusted for age, gender Model 2 adjusted for Model 1+marital status, education level, Hu kou, smoking status, drinking status Model 3 adjusted for Model 2+hypertension, diabetes, dyslipidemia, kidney disease Model 4 adjusted for Model 3+stages of CKM syndrome, SBP, DBP, HDL-C, LDL-C, eGFR, UA, Cr, BUN, Hb, MCV, PCV, platelet count, WBC | | | | | | | | | | | |

| **Table S10** Associations between TyG-related indices and CMM risk in CKM syndrome stage 1-3 | | | | | | | | | | | |
| --- | --- | --- | --- | --- | --- | --- | --- | --- | --- | --- | --- |
|  | Case | Crude |  | Model 1 |  | Model 2 |  | Model 3 |  | Model 4 |  |
|  |  | HR (95%CI) | P | HR (95%CI) | P | HR (95%CI) | P | HR (95%CI) | P | HR (95%CI) | P |
| TyG |  |  |  |  |  |  |  |  |  |  |  |
| Q1 | 85 (5.4) | 1(Ref) |  |  |  |  |  |  |  |  |  |
| Q2 | 151 (9.6) | 1.77 (1.36~2.31) | <0.001 | 1.78 (1.36~2.32) | <0.001 | 1.77 (1.36~2.31) | <0.001 | 1.7 (1.3~2.22) | <0.001 | 1.61 (1.23~2.11) | 0.001 |
| Q3 | 152 (9.6) | 1.81 (1.39~2.36) | <0.001 | 1.79 (1.37~2.34) | <0.001 | 1.76 (1.35~2.3) | <0.001 | 1.49 (1.13~1.97) | 0.004 | 1.34 (1.01~1.78) | 0.044 |
| Q4 | 252 (16) | 3.13 (2.45~4) | <0.001 | 3.18 (2.48~4.07) | <0.001 | 3.12 (2.44~4) | <0.001 | 1.92 (1.45~2.56) | <0.001 | 1.74 (1.29~2.35) | <0.001 |
| Trend test | 640 (10.1) | 1.41 (1.31~1.52) | <0.001 | 1.42 (1.32~1.52) | <0.001 | 1.41 (1.31~1.52) | <0.001 | 1.19 (1.09~1.29) | <0.001 | 1.15 (1.05~1.26) | 0.004 |
| TyG-BMI |  |  |  |  |  |  |  |  |  |  |  |
| Q1 | 89 (5.6) | 1(Ref) |  |  |  |  |  |  |  |  |  |
| Q2 | 124 (7.9) | 1.31 (1~1.73) | 0.049 | 1.47 (1.12~1.94) | 0.006 | 1.46 (1.11~1.93) | 0.007 | 1.33 (1.01~1.75) | 0.043 | 1.32 (1~1.75) | 0.05 |
| Q3 | 147 (9.3) | 1.61 (1.23~2.09) | <0.001 | 1.87 (1.43~2.44) | <0.001 | 1.84 (1.4~2.41) | <0.001 | 1.45 (1.1~1.92) | 0.008 | 1.42 (1.06~1.89) | 0.017 |
| Q4 | 280 (17.7) | 3.15 (2.48~3.99) | <0.001 | 3.77 (2.95~4.83) | <0.001 | 3.72 (2.9~4.77) | <0.001 | 2.38 (1.82~3.13) | <0.001 | 2.25 (1.68~3.01) | <0.001 |
| Trend test | 640 (10.1) | 1.49 (1.38~1.6) | <0.001 | 1.57 (1.45~1.69) | <0.001 | 1.56 (1.45~1.69) | <0.001 | 1.33 (1.23~1.45) | <0.001 | 1.3 (1.19~1.43) | <0.001 |
| TyG-WC |  |  |  |  |  |  |  |  |  |  |  |
| Q1 | 77 (4.9) | 1(Ref) |  |  |  |  |  |  |  |  |  |
| Q2 | 119 (7.5) | 1.53 (1.15~2.04) | 0.004 | 1.56 (1.17~2.08) | 0.003 | 1.55 (1.16~2.07) | 0.003 | 1.47 (1.1~1.97) | 0.008 | 1.44 (1.08~1.93) | 0.014 |
| Q3 | 162 (10.3) | 2.13 (1.62~2.79) | <0.001 | 2.19 (1.67~2.87) | <0.001 | 2.15 (1.64~2.83) | <0.001 | 1.81 (1.37~2.4) | <0.001 | 1.73 (1.3~2.31) | <0.001 |
| Q4 | 282 (17.9) | 3.89 (3.02~5) | <0.001 | 3.98 (3.09~5.13) | <0.001 | 3.91 (3.03~5.04) | <0.001 | 2.55 (1.93~3.38) | <0.001 | 2.42 (1.79~3.25) | <0.001 |
| Trend test | 640 (10.1) | 1.58 (1.47~1.7) | <0.001 | 1.59 (1.47~1.71) | <0.001 | 1.58 (1.46~1.71) | <0.001 | 1.35 (1.24~1.47) | <0.001 | 1.33 (1.21~1.45) | <0.001 |
| TyG-WHtR |  |  |  |  |  |  |  |  |  |  |  |
| Q1 | 75 (4.7) | 1(Ref) |  |  |  |  |  |  |  |  |  |
| Q2 | 117 (7.4) | 1.53 (1.14~2.04) | 0.004 | 1.62 (1.21~2.17) | 0.001 | 1.61 (1.2~2.15) | 0.001 | 1.48 (1.1~1.99) | 0.009 | 1.45 (1.08~1.95) | 0.014 |
| Q3 | 169 (10.7) | 2.22 (1.69~2.91) | <0.001 | 2.32 (1.76~3.06) | <0.001 | 2.28 (1.73~3.01) | <0.001 | 1.85 (1.4~2.46) | <0.001 | 1.78 (1.33~2.38) | <0.001 |
| Q4 | 279 (17.7) | 3.92 (3.04~5.06) | <0.001 | 4.1 (3.15~5.35) | <0.001 | 4.04 (3.1~5.28) | <0.001 | 2.52 (1.88~3.37) | <0.001 | 2.36 (1.74~3.21) | <0.001 |
| Trend test | 640 (10.1) | 1.59 (1.47~1.71) | <0.001 | 1.6 (1.48~1.73) | <0.001 | 1.59 (1.47~1.72) | <0.001 | 1.34 (1.23~1.47) | <0.001 | 1.31 (1.2~1.44) | <0.001 |
| TyG-BRI |  |  |  |  |  |  |  |  |  |  |  |
| Q1 | 76 (4.8) | 1(Ref) |  |  |  |  |  |  |  |  |  |
| Q2 | 117 (7.4) | 1.52 (1.14~2.03) | 0.005 | 1.61 (1.21~2.16) | 0.001 | 1.59 (1.19~2.13) | 0.002 | 1.43 (1.07~1.91) | 0.017 | 1.43 (1.06~1.92) | 0.018 |
| Q3 | 168 (10.6) | 2.18 (1.66~2.86) | <0.001 | 2.31 (1.75~3.04) | <0.001 | 2.26 (1.71~2.98) | <0.001 | 1.81 (1.37~2.4) | <0.001 | 1.77 (1.33~2.36) | <0.001 |
| Q4 | 279 (17.7) | 3.89 (3.02~5.01) | <0.001 | 4.12 (3.16~5.38) | <0.001 | 4.07 (3.12~5.32) | <0.001 | 2.63 (1.98~3.48) | <0.001 | 2.51 (1.87~3.37) | <0.001 |
| Trend test | 640 (10.1) | 1.58 (1.47~1.71) | <0.001 | 1.6 (1.48~1.73) | <0.001 | 1.6 (1.47~1.73) | <0.001 | 1.37 (1.26~1.49) | <0.001 | 1.35 (1.23~1.47) | <0.001 |
| TyG-WWI |  |  |  |  |  |  |  |  |  |  |  |
| Q1 | 85 (5.4) | 1(Ref) |  |  |  |  |  |  |  |  |  |
| Q2 | 121 (7.7) | 1.44 (1.09~1.9) | 0.01 | 1.42 (1.08~1.88) | 0.013 | 1.41 (1.07~1.87) | 0.015 | 1.28 (0.97~1.7) | 0.082 | 1.24 (0.93~1.64) | 0.138 |
| Q3 | 178 (11.3) | 2.12 (1.64~2.75) | <0.001 | 2.11 (1.62~2.74) | <0.001 | 2.08 (1.6~2.71) | <0.001 | 1.65 (1.26~2.16) | <0.001 | 1.53 (1.16~2.01) | 0.003 |
| Q4 | 256 (16.2) | 3.28 (2.56~4.19) | <0.001 | 3.13 (2.41~4.06) | <0.001 | 3.1 (2.39~4.02) | <0.001 | 1.81 (1.36~2.41) | <0.001 | 1.65 (1.22~2.22) | 0.001 |
| Trend test | 640 (10.1) | 1.49 (1.39~1.61) | <0.001 | 1.47 (1.36~1.59) | <0.001 | 1.46 (1.35~1.58) | <0.001 | 1.22 (1.11~1.33) | <0.001 | 1.18 (1.07~1.29) | 0.001 |
| TyG-CVAI |  |  |  |  |  |  |  |  |  |  |  |
| Q1 | 67 (4.2) | 1(Ref) |  |  |  |  |  |  |  |  |  |
| Q2 | 125 (7.9) | 1.85 (1.38~2.5) | <0.001 | 1.82 (1.35~2.45) | <0.001 | 1.81 (1.34~2.44) | <0.001 | 1.72 (1.27~2.32) | <0.001 | 1.72 (1.27~2.33) | 0.001 |
| Q3 | 166 (10.5) | 2.52 (1.9~3.35) | <0.001 | 2.41 (1.81~3.22) | <0.001 | 2.36 (1.77~3.15) | <0.001 | 1.97 (1.47~2.65) | <0.001 | 1.98 (1.45~2.7) | <0.001 |
| Q4 | 282 (17.9) | 4.55 (3.49~5.94) | <0.001 | 4.22 (3.22~5.53) | <0.001 | 4.13 (3.14~5.42) | <0.001 | 2.74 (2.04~3.69) | <0.001 | 2.75 (1.99~3.82) | <0.001 |
| Trend test | 640 (10.1) | 1.62 (1.51~1.75) | <0.001 | 1.58 (1.46~1.71) | <0.001 | 1.57 (1.45~1.7) | <0.001 | 1.35 (1.24~1.47) | <0.001 | 1.35 (1.23~1.49) | <0.001 |
| TyG-ABSI |  |  |  |  |  |  |  |  |  |  |  |
| Q1 | 84 (5.3) | 1(Ref) |  |  |  |  |  |  |  |  |  |
| Q2 | 139 (8.8) | 1.71 (1.3~2.24) | <0.001 | 1.65 (1.26~2.17) | <0.001 | 1.65 (1.26~2.17) | <0.001 | 1.51 (1.15~1.99) | 0.003 | 1.45 (1.1~1.91) | 0.009 |
| Q3 | 172 (10.9) | 2.14 (1.65~2.78) | <0.001 | 2.02 (1.55~2.62) | <0.001 | 2 (1.53~2.6) | <0.001 | 1.64 (1.25~2.15) | <0.001 | 1.52 (1.15~1.99) | 0.003 |
| Q4 | 245 (15.5) | 3.25 (2.54~4.16) | <0.001 | 2.93 (2.27~3.77) | <0.001 | 2.91 (2.26~3.76) | <0.001 | 1.81 (1.37~2.39) | <0.001 | 1.67 (1.25~2.23) | <0.001 |
| Trend test | 640 (10.1) | 1.45 (1.34~1.55) | <0.001 | 1.4  (1.3~1.5) | <0.001 | 1.39 (1.29~1.5) | <0.001 | 1.18 (1.09~1.28) | <0.001 | 1.15 (1.06~1.26) | 0.001 |
| CTI |  |  |  |  |  |  |  |  |  |  |  |
| Q1 | 78 (4.9) | 1(Ref) |  |  |  |  |  |  |  |  |  |
| Q2 | 131 (8.3) | 1.73 (1.31~2.29) | <0.001 | 1.67 (1.26~2.22) | <0.001 | 1.67 (1.26~2.21) | <0.001 | 1.6 (1.21~2.12) | 0.001 | 1.55 (1.16~2.06) | 0.003 |
| Q3 | 171 (10.8) | 2.32 (1.78~3.04) | <0.001 | 2.25 (1.72~2.94) | <0.001 | 2.22 (1.7~2.91) | <0.001 | 1.84 (1.4~2.44) | <0.001 | 1.69 (1.27~2.26) | <0.001 |
| Q4 | 260 (16.5) | 3.74 (2.9~4.81) | <0.001 | 3.61 (2.8~4.65) | <0.001 | 3.55 (2.75~4.58) | <0.001 | 2.32 (1.75~3.08) | <0.001 | 2.09 (1.54~2.83) | <0.001 |
| Trend test | 640 (10.1) | 1.52 (1.41~1.64) | <0.001 | 1.51 (1.4~1.63) | <0.001 | 1.5 (1.39~1.62) | <0.001 | 1.29 (1.18~1.4) | <0.001 | 1.24 (1.13~1.36) | <0.001 |
| CMM, cardiometabolic multimorbidity; CKM syndrome, cardiovascular kidney-metabolic syndrome; TyG, triglyceride-glucose index; TyG-BMI, triglyceride glucose-body mass index; TyG-WC, triglyceride glucose-waist circumference; TyG-WHtR, triglyceride glucose-waist height ratio index; TyG-BRI, triglyceride glucose-body roundness index; TyG-WWI, triglyceride glucose-weight-adjusted waist index; TyG-CVAI, triglyceride glucose-Chinese visceral adiposity index; TyG-ABSI, triglyceride glucose-a body shape index; CTI, C-reactive protein-triglyceride-glucose index; SBP, systolic blood pressure; DBP, diastolic blood pressure; HDL-C, high-density lipoprotein cholesterol; LDL-C, low-density lipoprotein cholesterol; eGFR, estimated glomerular filtration rate; UA, uric acid; Cr, creatinine; BUN, blood urea nitrogen; Hb, hemoglobin; MCV, mean corpuscular volume; PCV, packed cell volume; WBC, white blood cell count Model 1 adjusted for age, gender Model 2 adjusted for Model 1+marital status, education level, Hu kou, smoking status, drinking status Model 3 adjusted for Model 2+hypertension, diabetes, dyslipidemia, kidney disease Model 4 adjusted for Model 3+stages of CKM syndrome, SBP, DBP, HDL-C, LDL-C, eGFR, UA, Cr, BUN, Hb, MCV, PCV, platelet count, WBC | | | | | | | | | | | |

| **Table S11** Associations between TyG-related indices and CMM risk in CKM syndrome stage 0-3 after including individuals with missing data | | | | | | | | | | | |
| --- | --- | --- | --- | --- | --- | --- | --- | --- | --- | --- | --- |
|  | Case | Crude |  | Model 1 |  | Model 2 |  | Model 3 |  | Model 4 |  |
|  |  | HR (95%CI) | P | HR (95%CI) | P | HR (95%CI) | P | HR (95%CI) | P | HR (95%CI) | P |
| TyG |  |  |  |  |  |  |  |  |  |  |  |
| Q1 | 100 (5.2) | 1(Ref) |  |  |  |  |  |  |  |  |  |
| Q2 | 153 (7.9) | 1.52 (1.19~1.96) | 0.001 | 1.52 (1.18~1.95) | 0.001 | 1.51 (1.17~1.94) | 0.001 | 1.4 (1.09~1.81) | 0.009 | 1.3  (1~1.68) | 0.047 |
| Q3 | 187 (9.7) | 1.91 (1.5~2.44) | <0.001 | 1.85 (1.45~2.36) | <0.001 | 1.82 (1.43~2.33) | <0.001 | 1.45 (1.12~1.87) | 0.004 | 1.22 (0.94~1.59) | 0.142 |
| Q4 | 310 (16.1) | 3.31 (2.64~4.15) | <0.001 | 3.31 (2.64~4.15) | <0.001 | 3.24 (2.58~4.06) | <0.001 | 1.73 (1.32~2.25) | <0.001 | 1.44 (1.08~1.92) | 0.012 |
| Trend test | 750 (9.7) | 1.48 (1.38~1.59) | <0.001 | 1.48 (1.38~1.59) | <0.001 | 1.47 (1.37~1.58) | <0.001 | 1.17 (1.08~1.28) | <0.001 | 1.11 (1.01~1.21) | 0.029 |
| TyG-BMI |  |  |  |  |  |  |  |  |  |  |  |
| Q1 | 90 (4.7) | 1(Ref) |  |  |  |  |  |  |  |  |  |
| Q2 | 138 (7.2) | 1.5 (1.15~1.96) | 0.003 | 1.62 (1.24~2.12) | <0.001 | 1.62 (1.24~2.11) | <0.001 | 1.41 (1.08~1.85) | 0.012 | 1.34 (1.02~1.77) | 0.034 |
| Q3 | 183 (9.5) | 2.01 (1.56~2.59) | <0.001 | 2.24 (1.73~2.89) | <0.001 | 2.19 (1.69~2.82) | <0.001 | 1.59 (1.22~2.07) | 0.001 | 1.47 (1.11~1.94) | 0.007 |
| Q4 | 339 (17.6) | 3.86 (3.06~4.87) | <0.001 | 4.46 (3.52~5.66) | <0.001 | 4.35 (3.43~5.53) | <0.001 | 2.47 (1.89~3.22) | <0.001 | 2.23 (1.67~2.98) | <0.001 |
| Trend test | 750 (9.7) | 1.58 (1.48~1.7) | <0.001 | 1.65 (1.54~1.77) | <0.001 | 1.64 (1.52~1.76) | <0.001 | 1.34 (1.24~1.46) | <0.001 | 1.3 (1.19~1.42) | <0.001 |
| TyG-WC |  |  |  |  |  |  |  |  |  |  |  |
| Q1 | 88 (4.6) | 1(Ref) |  |  |  |  |  |  |  |  |  |
| Q2 | 133 (6.9) | 1.52 (1.16~1.99) | 0.002 | 1.53 (1.17~2.01) | 0.002 | 1.53 (1.16~2) | 0.002 | 1.36 (1.04~1.79) | 0.026 | 1.27 (0.96~1.67) | 0.096 |
| Q3 | 183 (9.5) | 2.12 (1.64~2.73) | <0.001 | 2.13 (1.65~2.74) | <0.001 | 2.08 (1.61~2.68) | <0.001 | 1.62 (1.25~2.11) | <0.001 | 1.45 (1.1~1.91) | 0.008 |
| Q4 | 346 (18) | 4.23 (3.34~5.34) | <0.001 | 4.25 (3.36~5.38) | <0.001 | 4.13 (3.26~5.23) | <0.001 | 2.32 (1.78~3.03) | <0.001 | 2.06 (1.54~2.74) | <0.001 |
| Trend test | 750 (9.7) | 1.64 (1.53~1.76) | <0.001 | 1.64 (1.53~1.76) | <0.001 | 1.62 (1.51~1.74) | <0.001 | 1.32 (1.21~1.43) | <0.001 | 1.27 (1.16~1.39) | <0.001 |
| TyG-WHtR |  |  |  |  |  |  |  |  |  |  |  |
| Q1 | 88 (4.6) | 1(Ref) |  |  |  |  |  |  |  |  |  |
| Q2 | 126 (6.5) | 1.44 (1.09~1.88) | 0.009 | 1.47 (1.12~1.93) | 0.006 | 1.46 (1.11~1.92) | 0.007 | 1.3 (0.99~1.71) | 0.062 | 1.22 (0.92~1.61) | 0.168 |
| Q3 | 197 (10.2) | 2.25 (1.75~2.89) | <0.001 | 2.3 (1.78~2.96) | <0.001 | 2.23 (1.73~2.88) | <0.001 | 1.68 (1.29~2.18) | <0.001 | 1.5 (1.14~1.98) | 0.004 |
| Q4 | 339 (17.6) | 4.15 (3.29~5.25) | <0.001 | 4.24 (3.33~5.4) | <0.001 | 4.14 (3.24~5.27) | <0.001 | 2.28 (1.74~2.98) | <0.001 | 2.02 (1.51~2.7) | <0.001 |
| Trend test | 750 (9.7) | 1.64 (1.53~1.76) | <0.001 | 1.65 (1.53~1.77) | <0.001 | 1.63 (1.52~1.76) | <0.001 | 1.32 (1.21~1.43) | <0.001 | 1.27 (1.16~1.39) | <0.001 |
| TyG-BRI |  |  |  |  |  |  |  |  |  |  |  |
| Q1 | 92 (4.8) | 1(Ref) |  |  |  |  |  |  |  |  |  |
| Q2 | 129 (6.7) | 1.39 (1.07~1.82) | 0.015 | 1.43 (1.09~1.87) | 0.009 | 1.42 (1.09~1.86) | 0.011 | 1.24 (0.94~1.62) | 0.123 | 1.17 (0.89~1.54) | 0.261 |
| Q3 | 196 (10.2) | 2.12 (1.66~2.72) | <0.001 | 2.19 (1.7~2.81) | <0.001 | 2.13 (1.66~2.74) | <0.001 | 1.55 (1.2~2) | 0.001 | 1.42 (1.08~1.85) | 0.011 |
| Q4 | 333 (17.3) | 3.87 (3.07~4.87) | <0.001 | 3.98 (3.13~5.07) | <0.001 | 3.9 (3.06~4.96) | <0.001 | 2.2 (1.69~2.85) | <0.001 | 1.98 (1.51~2.61) | <0.001 |
| Trend test | 750 (9.7) | 1.6 (1.49~1.72) | <0.001 | 1.61 (1.5~1.74) | <0.001 | 1.6 (1.49~1.72) | <0.001 | 1.31 (1.21~1.42) | <0.001 | 1.27 (1.17~1.38) | <0.001 |
| TyG-WWI |  |  |  |  |  |  |  |  |  |  |  |
| Q1 | 102 (5.3) | 1(Ref) |  |  |  |  |  |  |  |  |  |
| Q2 | 124 (6.4) | 1.23 (0.95~1.6) | 0.12 | 1.21 (0.93~1.57) | 0.159 | 1.21 (0.93~1.57) | 0.16 | 1.08 (0.83~1.41) | 0.555 | 1.02 (0.78~1.33) | 0.881 |
| Q3 | 217 (11.3) | 2.19 (1.73~2.77) | <0.001 | 2.15 (1.7~2.73) | <0.001 | 2.14 (1.69~2.72) | <0.001 | 1.6 (1.25~2.04) | <0.001 | 1.39 (1.08~1.79) | 0.01 |
| Q4 | 307 (15.9) | 3.32 (2.66~4.16) | <0.001 | 3.16  (2.5~4) | <0.001 | 3.13 (2.48~3.97) | <0.001 | 1.61 (1.24~2.1) | <0.001 | 1.39 (1.06~1.83) | 0.018 |
| Trend test | 750 (9.7) | 1.54 (1.44~1.65) | <0.001 | 1.51 (1.41~1.63) | <0.001 | 1.51 (1.4~1.62) | <0.001 | 1.19 (1.1~1.29) | <0.001 | 1.14 (1.04~1.24) | 0.004 |
| TyG-CVAI |  |  |  |  |  |  |  |  |  |  |  |
| Q1 | 84 (4.4) | 1(Ref) |  |  |  |  |  |  |  |  |  |
| Q2 | 130 (6.8) | 1.54 (1.17~2.03) | 0.002 | 1.51 (1.14~1.99) | 0.004 | 1.49 (1.13~1.97) | 0.004 | 1.32 (1~1.74) | 0.052 | 1.26 (0.94~1.67) | 0.118 |
| Q3 | 199 (10.3) | 2.43 (1.89~3.14) | <0.001 | 2.31 (1.79~3) | <0.001 | 2.25 (1.73~2.92) | <0.001 | 1.7 (1.3~2.23) | <0.001 | 1.57 (1.18~2.09) | 0.002 |
| Q4 | 337 (17.5) | 4.38 (3.45~5.56) | <0.001 | 4.05 (3.18~5.17) | <0.001 | 3.91 (3.06~5) | <0.001 | 2.23 (1.7~2.93) | <0.001 | 2.08 (1.53~2.83) | <0.001 |
| Trend test | 750 (9.7) | 1.65 (1.54~1.78) | <0.001 | 1.61 (1.5~1.73) | <0.001 | 1.59 (1.48~1.71) | <0.001 | 1.31 (1.2~1.42) | <0.001 | 1.28 (1.17~1.41) | <0.001 |
| TyG-ABSI |  |  |  |  |  |  |  |  |  |  |  |
| Q1 | 104 (5.4) | 1(Ref) |  |  |  |  |  |  |  |  |  |
| Q2 | 149 (7.7) | 1.46 (1.13~1.87) | 0.003 | 1.4 (1.09~1.8) | 0.008 | 1.41 (1.09~1.81) | 0.008 | 1.29 (1.01~1.67) | 0.045 | 1.18 (0.91~1.52) | 0.202 |
| Q3 | 195 (10.1) | 1.97 (1.55~2.5) | <0.001 | 1.84 (1.45~2.34) | <0.001 | 1.83 (1.44~2.32) | <0.001 | 1.4 (1.09~1.79) | 0.008 | 1.21 (0.94~1.56) | 0.145 |
| Q4 | 302 (15.7) | 3.24 (2.59~4.05) | <0.001 | 2.92 (2.33~3.67) | <0.001 | 2.91 (2.31~3.65) | <0.001 | 1.59 (1.24~2.05) | <0.001 | 1.37 (1.06~1.79) | 0.018 |
| Trend test | 750 (9.7) | 1.48 (1.38~1.59) | <0.001 | 1.43 (1.33~1.53) | <0.001 | 1.43 (1.33~1.53) | <0.001 | 1.15 (1.07~1.24) | <0.001 | 1.1 (1.01~1.19) | 0.021 |
| CTI |  |  |  |  |  |  |  |  |  |  |  |
| Q1 | 83 (4.3) | 1(Ref) |  |  |  |  |  |  |  |  |  |
| Q2 | 153 (7.9) | 1.91 (1.46~2.49) | <0.001 | 1.83 (1.4~2.4) | <0.001 | 1.83 (1.4~2.39) | <0.001 | 1.74 (1.33~2.28) | <0.001 | 1.62 (1.23~2.13) | 0.001 |
| Q3 | 189 (9.8) | 2.41 (1.86~3.12) | <0.001 | 2.31 (1.79~2.99) | <0.001 | 2.29 (1.77~2.96) | <0.001 | 1.81 (1.38~2.37) | <0.001 | 1.58 (1.2~2.1) | 0.001 |
| Q4 | 325 (16.9) | 4.41 (3.47~5.62) | <0.001 | 4.21 (3.31~5.36) | <0.001 | 4.12 (3.23~5.25) | <0.001 | 2.39 (1.82~3.15) | <0.001 | 2.07 (1.54~2.79) | <0.001 |
| Trend test | 750 (9.7) | 1.6 (1.49~1.71) | <0.001 | 1.58 (1.47~1.69) | <0.001 | 1.57 (1.46~1.68) | <0.001 | 1.28 (1.18~1.39) | <0.001 | 1.22 (1.12~1.34) | <0.001 |
| CMM, cardiometabolic multimorbidity; CKM syndrome, cardiovascular kidney-metabolic syndrome; TyG, triglyceride-glucose index; TyG-BMI, triglyceride glucose-body mass index; TyG-WC, triglyceride glucose-waist circumference; TyG-WHtR, triglyceride glucose-waist height ratio index; TyG-BRI, triglyceride glucose-body roundness index; TyG-WWI, triglyceride glucose-weight-adjusted waist index; TyG-CVAI, triglyceride glucose-Chinese visceral adiposity index; TyG-ABSI, triglyceride glucose-a body shape index; CTI, C-reactive protein-triglyceride-glucose index; SBP, systolic blood pressure; DBP, diastolic blood pressure; HDL-C, high-density lipoprotein cholesterol; LDL-C, low-density lipoprotein cholesterol; eGFR, estimated glomerular filtration rate; UA, uric acid; Cr, creatinine; BUN, blood urea nitrogen; Hb, hemoglobin; MCV, mean corpuscular volume; PCV, packed cell volume; WBC, white blood cell count Model 1 adjusted for age, gender Model 2 adjusted for Model 1+marital status, education level, Hu kou, smoking status, drinking status Model 3 adjusted for Model 2+hypertension, diabetes, dyslipidemia, kidney disease Model 4 adjusted for Model 3+stages of CKM syndrome, SBP, DBP, HDL-C, LDL-C, eGFR, UA, Cr, BUN, Hb, MCV, PCV, platelet count, WBC | | | | | | | | | | | |

| **Table S12** Optimal thresholds, sensitivity, specificity, NPV and PPV of TyG-related indices for predicting CMM, stroke, and CHD | | | | | | |
| --- | --- | --- | --- | --- | --- | --- |
| TyG-related Index | Optimal Cut-off | Sensitivity | Specificity (%) | PPV | NPV | Youden’s index |
| For CMM |  |  |  |  |  |  |
| TyG | 8.725 | 0.5798 | 0.6071 | 0.134 | 0.9323 | 1.1868 |
| TyG-BMI | 211.8488 | 0.5828 | 0.6626 | 0.1534 | 0.938 | 1.2454 |
| TyG-WC | 764.6158 | 0.6212 | 0.6528 | 0.158 | 0.9426 | 1.2739 |
| TyG-WHtR | 4.8677 | 0.6212 | 0.6597 | 0.1607 | 0.9432 | 1.2809 |
| TyG-BRI | 35.9807 | 0.6871 | 0.5632 | 0.1416 | 0.9449 | 1.2503 |
| TyG-WWI | 98.7475 | 0.6273 | 0.62 | 0.1476 | 0.9407 | 1.2473 |
| TyG-CVAI | 1082.5421 | 0.5644 | 0.7012 | 0.1654 | 0.9388 | 1.2656 |
| TyG-ABSI | 0.722 | 0.6365 | 0.5619 | 0.1322 | 0.9364 | 1.1984 |
| CTI | 8.7708 | 0.6488 | 0.5821 | 0.1401 | 0.9405 | 1.2309 |
| For stroke |  |  |  |  |  |  |
| TyG | 8.2851 | 0.82 | 0.3046 | 0.0996 | 0.9475 | 1.1246 |
| TyG-BMI | 198.4546 | 0.6095 | 0.5169 | 0.1058 | 0.9338 | 1.1264 |
| TyG-WC | 753.1569 | 0.5501 | 0.6039 | 0.1153 | 0.9347 | 1.1539 |
| TyG-WHtR | 4.6122 | 0.6418 | 0.5151 | 0.1105 | 0.9388 | 1.1569 |
| TyG-BRI | 34.9049 | 0.6282 | 0.5229 | 0.11 | 0.9375 | 1.1511 |
| TyG-WWI | 98.8436 | 0.5229 | 0.6115 | 0.1121 | 0.9318 | 1.1344 |
| TyG-CVAI | 899.1522 | 0.657 | 0.5223 | 0.1143 | 0.942 | 1.1793 |
| TyG-ABSI | 0.6835 | 0.7929 | 0.3334 | 0.1004 | 0.9449 | 1.1263 |
| CTI | 8.3513 | 0.7929 | 0.3654 | 0.1049 | 0.9495 | 1.1583 |
| For CHD |  |  |  |  |  |  |
| TyG | 8.725 | 0.4619 | 0.6013 | 0.2128 | 0.8273 | 1.0632 |
| TyG-BMI | 208.1034 | 0.4781 | 0.6239 | 0.2287 | 0.8367 | 1.102 |
| TyG-WC | 711.993 | 0.632 | 0.4776 | 0.2201 | 0.8476 | 1.1096 |
| TyG-WHtR | 4.3783 | 0.7129 | 0.3978 | 0.2164 | 0.8559 | 1.1107 |
| TyG-BRI | 38.8575 | 0.4727 | 0.6408 | 0.2349 | 0.8389 | 1.1135 |
| TyG-WWI | 95.215 | 0.6082 | 0.4928 | 0.2186 | 0.8435 | 1.101 |
| TyG-CVAI | 746.285 | 0.7621 | 0.3718 | 0.2206 | 0.8701 | 1.1339 |
| TyG-ABSI | 0.7119 | 0.5743 | 0.5036 | 0.2125 | 0.8353 | 1.0779 |
| CTI | 8.2049 | 0.7821 | 0.2902 | 0.2045 | 0.851 | 1.0724 |
| CMM, cardiometabolic multimorbidity; CHD, coronary heart disease; NPV, negative predictive value; PPV, positive predictive value; TyG, triglyceride-glucose index; TyG-BMI, triglyceride glucose-body mass index; TyG-WC, triglyceride glucose-waist circumference; TyG-WHtR, triglyceride glucose-waist height ratio index; TyG-BRI, triglyceride glucose-body roundness index; TyG-WWI, triglyceride glucose-weight-adjusted waist index; TyG-CVAI, triglyceride glucose-Chinese visceral adiposity index; TyG-ABSI, triglyceride glucose-a body shape index; CTI, C-reactive protein-triglyceride-glucose index | | | | | | |

| **Table S13** Schoenfeld residual test results for the Cox proportional hazards models of the associations between TyG-related indices and CMM in patients with CKM syndrome stage 0-3 | | | |
| --- | --- | --- | --- |
| Variable | chisq(χ²) | df | p-value |
| TyG | 0.894 | 1 | 0.344 |
| TyG-BMI | 0.009 | 1 | 0.924 |
| TyG-WC | 3.67 | 1 | 0.055 |
| TyG-WHtR | 3.019 | 1 | 0.082 |
| TyG-BRI | 1.292 | 1 | 0.256 |
| TyG-WWI | 2.106 | 1 | 0.147 |
| TyG-CVAI | 0.019 | 1 | 0.889 |
| TyG-ABSI | 1.402 | 1 | 0.236 |
| CTI | 1.148 | 1 | 0.284 |
| Global test | 5.763 | 9 | 0.763 |
| CMM, cardiometabolic multimorbidity; CKM syndrome, cardiovascular kidney-metabolic syndrome; TyG, triglyceride-glucose index; TyG-BMI, triglyceride glucose-body mass index; TyG-WC, triglyceride glucose-waist circumference; TyG-WHtR, triglyceride glucose-waist height ratio index; TyG-BRI, triglyceride glucose-body roundness index; TyG-WWI, triglyceride glucose-weight-adjusted waist index; TyG-CVAI, triglyceride glucose-Chinese visceral adiposity index; TyG-ABSI, triglyceride glucose-a body shape index; CTI, C-reactive protein-triglyceride-glucose index  All p-values were greater than 0.05, indicating that the proportional hazards assumption was not violated for any of the TyG-related indices or in the global test. | | | |

| **Table S14** Schoenfeld residual test results for the Cox proportional hazards models of the associations between TyG-related indices and CMM in patients with CKM syndrome stage 0-3 | | | |
| --- | --- | --- | --- |
| Variable | chisq(χ²) | df | p-value |
| TyG | 0.353 | 1 | 0.552 |
| TyG-BMI | 0.005 | 1 | 0.942 |
| TyG-WC | 0.218 | 1 | 0.641 |
| TyG-WHtR | 0.005 | 1 | 0.944 |
| TyG-BRI | 0.007 | 1 | 0.931 |
| TyG-WWI | 0.063 | 1 | 0.802 |
| TyG-CVAI | 0.077 | 1 | 0.781 |
| TyG-ABSI | 0.37 | 1 | 0.543 |
| CTI | 0.094 | 1 | 0.759 |
| Global test | 5.78 | 9 | 0.762 |
| CKM syndrome, cardiovascular kidney-metabolic syndrome; TyG, triglyceride-glucose index; TyG-BMI, triglyceride glucose-body mass index; TyG-WC, triglyceride glucose-waist circumference; TyG-WHtR, triglyceride glucose-waist height ratio index; TyG-BRI, triglyceride glucose-body roundness index; TyG-WWI, triglyceride glucose-weight-adjusted waist index; TyG-CVAI, triglyceride glucose-Chinese visceral adiposity index; TyG-ABSI, triglyceride glucose-a body shape index; CTI, C-reactive protein-triglyceride-glucose index  All p-values were greater than 0.05, indicating that the proportional hazards assumption was not violated for any of the TyG-related indices or in the global test. | | | |

| **Table S15** Schoenfeld residual test results for the Cox proportional hazards models of the associations between TyG-related indices and CMM in patients with CKM syndrome stage 0-3 | | | |
| --- | --- | --- | --- |
| Variable | chisq(χ²) | df | p-value |
| TyG | 1.216 | 1 | 0.27 |
| TyG-BMI | 0.834 | 1 | 0.361 |
| TyG-WC | 0.962 | 1 | 0.327 |
| TyG-WHtR | 1.46 | 1 | 0.227 |
| TyG-BRI | 1.167 | 1 | 0.28 |
| TyG-WWI | 1.493 | 1 | 0.222 |
| TyG-CVAI | 2.299 | 1 | 0.129 |
| TyG-ABSI | 1.04 | 1 | 0.308 |
| CTI | 0.288 | 1 | 0.592 |
| Global test | 7.057 | 9 | 0.631 |
| CHD, coronary heart disease; CKM syndrome, cardiovascular kidney-metabolic syndrome; TyG, triglyceride-glucose index; TyG-BMI, triglyceride glucose-body mass index; TyG-WC, triglyceride glucose-waist circumference; TyG-WHtR, triglyceride glucose-waist height ratio index; TyG-BRI, triglyceride glucose-body roundness index; TyG-WWI, triglyceride glucose-weight-adjusted waist index; TyG-CVAI, triglyceride glucose-Chinese visceral adiposity index; TyG-ABSI, triglyceride glucose-a body shape index; CTI, C-reactive protein-triglyceride-glucose index  All p-values were greater than 0.05, indicating that the proportional hazards assumption was not violated for any of the TyG-related indices or in the global test. | | | |

| **Table S16** Associations between TyG-related indices and incident CMM using Fine-Gray competing risk regression in participants with CKM syndrome stage 0-3 | | | | | |
| --- | --- | --- | --- | --- | --- |
| Variable | Events (n, %) | crude HR (95%CI) | crude P-value | adjusted HR (95%CI) | adjusted P-value |
| TyG (Continuous variable per SD) | 652 (9.5) | 1.54 (1.45~1.65) | <0.001 | 1.2 (1.08~1.32) | 0.001 |
| TyG-BMI (Continuous variable per SD) | 652 (9.5) | 1.68 (1.57~1.8) | <0.001 | 1.41 (1.28~1.56) | <0.001 |
| TyG-WC (Continuous variable per SD) | 652 (9.5) | 1.77 (1.65~1.9) | <0.001 | 1.45 (1.31~1.6) | <0.001 |
| TyG-WHtR (Continuous variable per SD) | 652 (9.5) | 1.76 (1.64~1.88) | <0.001 | 1.41 (1.28~1.56) | <0.001 |
| TyG-BRI (Continuous variable per SD) | 652 (9.5) | 1.63 (1.53~1.74) | <0.001 | 1.25 (1.13~1.37) | <0.001 |
| TyG-WWI (Continuous variable per SD) | 652 (9.5) | 1.66 (1.56~1.77) | <0.001 | 1.34 (1.22~1.46) | <0.001 |
| TyG-CVAI (Continuous variable per SD) | 652 (9.5) | 1.78 (1.67~1.91) | <0.001 | 1.46 (1.31~1.62) | <0.001 |
| TyG-ABSI (Continuous variable per SD) | 652 (9.5) | 1.55 (1.46~1.66) | <0.001 | 1.17 (1.07~1.29) | 0.001 |
| CTI (Continuous variable per SD) | 652 (9.5) | 1.62 (1.52~1.73) | <0.001 | 1.25 (1.14~1.37) | <0.001 |
| CMM, cardiometabolic multimorbidity; CKM syndrome, cardiovascular kidney-metabolic syndrome; TyG, triglyceride-glucose index; TyG-BMI, triglyceride glucose-body mass index; TyG-WC, triglyceride glucose-waist circumference; TyG-WHtR, triglyceride glucose-waist height ratio index; TyG-BRI, triglyceride glucose-body roundness index; TyG-WWI, triglyceride glucose-weight-adjusted waist index; TyG-CVAI, triglyceride glucose-Chinese visceral adiposity index; TyG-ABSI, triglyceride glucose-a body shape index; CTI, C-reactive protein-triglyceride-glucose index  Adjusted for age, gender, marital status, education level, Hu kou, smoking status, drinking status, hypertension, diabetes, dyslipidemia, kidney disease, stages of CKM syndrome, SBP, DBP, HDL-C, LDL-C, eGFR, UA, Cr, BUN, Hb, MCV, PCV, platelet count, WBC | | | | | |


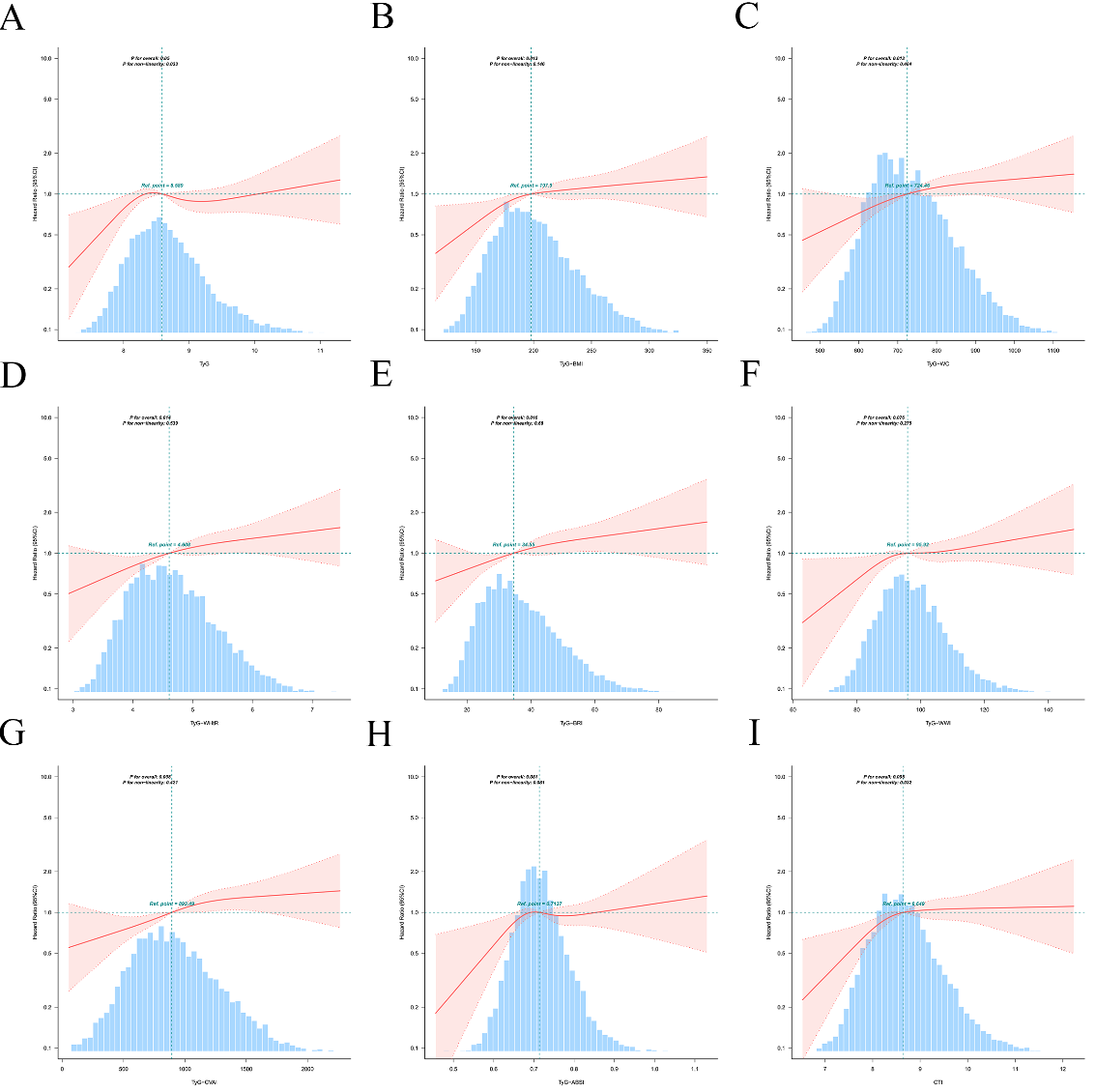


**Fig.S1** Dose-response relationships between TyG-related indices and stroke risk. Solid lines indicate HRs and shadow shapes indicate 95% CI. The y-axis represents the adjusted HR for the CMM. A. TyG, triglyceride-glucose index; B. TyG-BMI, triglyceride glucose-body mass index; C. TyG-WC, triglyceride glucose-waist circumference; D. TyG-WHtR, triglyceride glucose-waist height ratio index; E. TyG-BRI, triglyceride glucose-body roundness index; F. TyG-WWI, triglyceride glucose-weight-adjusted waist index; G. TyG-CVAI, triglyceride glucose-Chinese visceral adiposity index; H. TyG-ABSI, triglyceride glucose-a body shape index; I. CTI, C-reactive protein-triglyceride-glucose index; HR, hazard ratio; CI, confidence interval


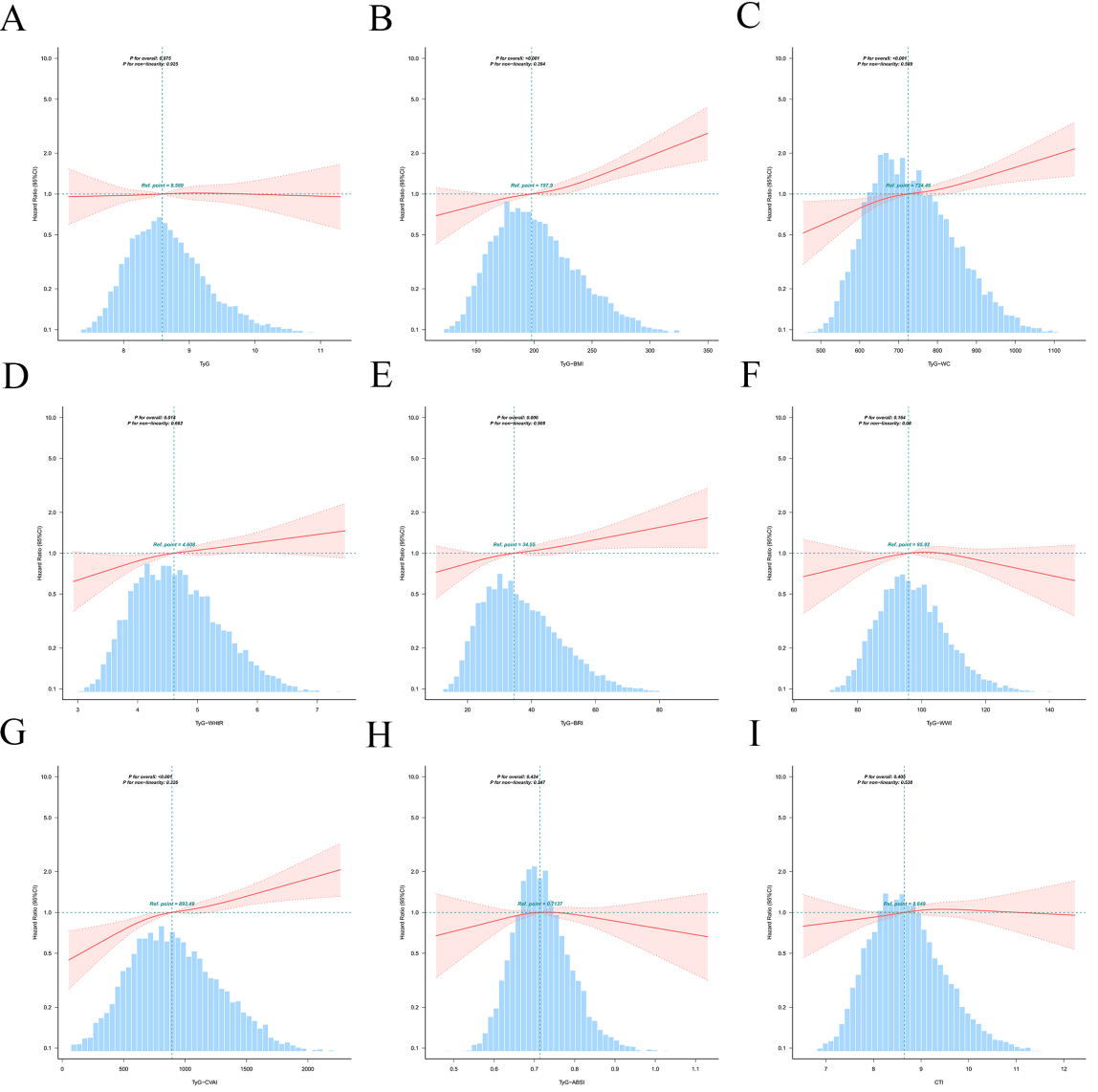


**Fig.S2** Dose-response relationships between TyG-related indices and CHD risk. Solid lines indicate HRs and shadow shapes indicate 95% CI. The y-axis represents the adjusted HR for the CMM. A. TyG, triglyceride-glucose index; B. TyG-BMI, triglyceride glucose-body mass index; C. TyG-WC, triglyceride glucose-waist circumference; D. TyG-WHtR, triglyceride glucose-waist height ratio index; E. TyG-BRI, triglyceride glucose-body roundness index; F. TyG-WWI, triglyceride glucose-weight-adjusted waist index; G. TyG-CVAI, triglyceride glucose-Chinese visceral adiposity index; H. TyG-ABSI, triglyceride glucose-a body shape index; I. CTI, C-reactive protein-triglyceride-glucose index; HR, hazard ratio; CI, confidence interval; CHD, coronary heart disease

**
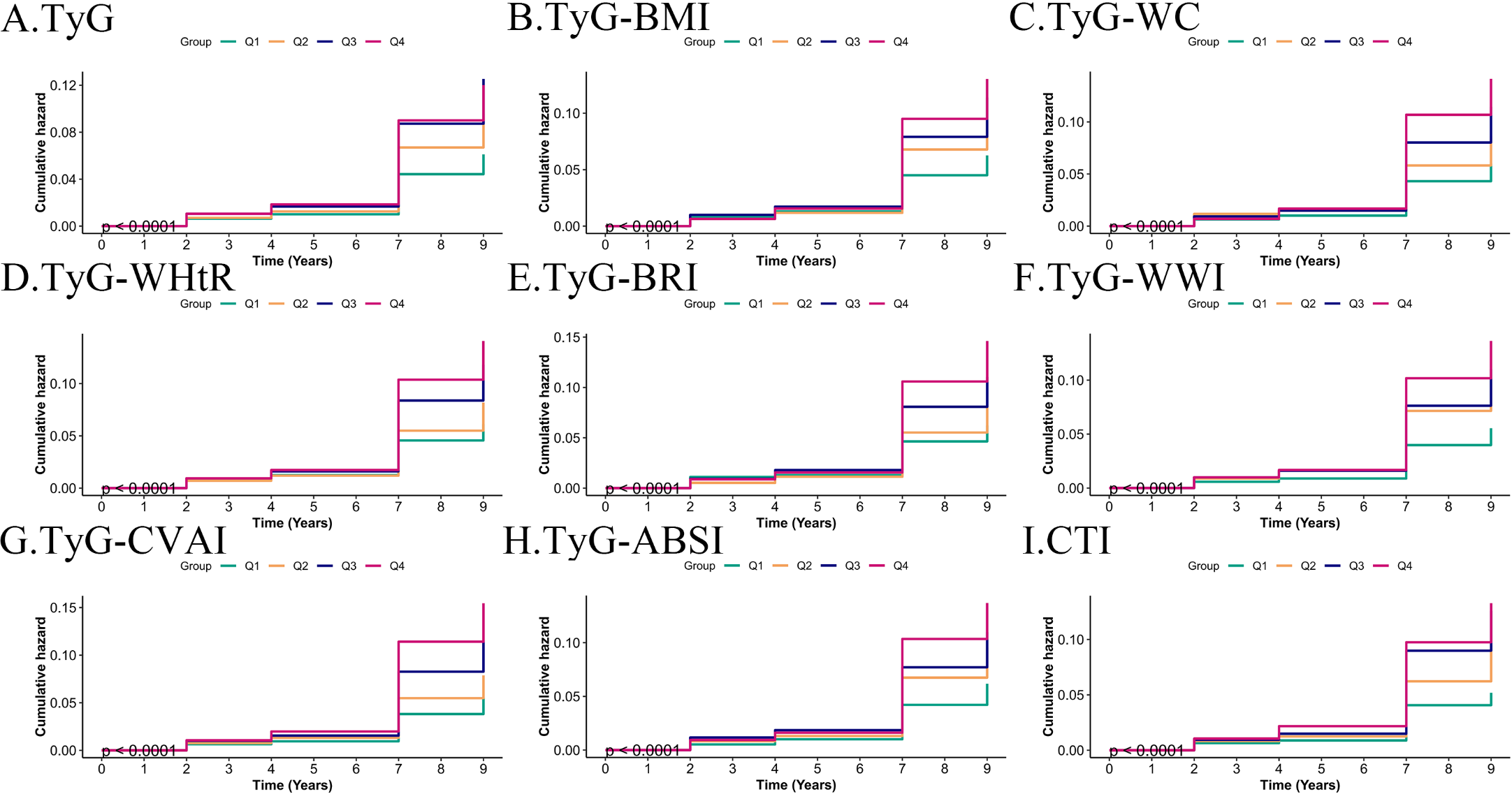
**

**Fig.S3** Kaplan-Meier curves for cumulative incidence of stroke by quartiles of TyG-related indices. A. TyG, triglyceride-glucose index; B. TyG-BMI, triglyceride glucose-body mass index; C. TyG-WC, triglyceride glucose-waist circumference; D. TyG-WHtR, triglyceride glucose-waist height ratio index; E. TyG-BRI, triglyceride glucose-body roundness index; F. TyG-WWI, triglyceride glucose-weight-adjusted waist index; G. TyG-CVAI, triglyceride glucose-Chinese visceral adiposity index; H. TyG-ABSI, triglyceride glucose-a body shape index; I. CTI, C-reactive protein-triglyceride-glucose index.

**
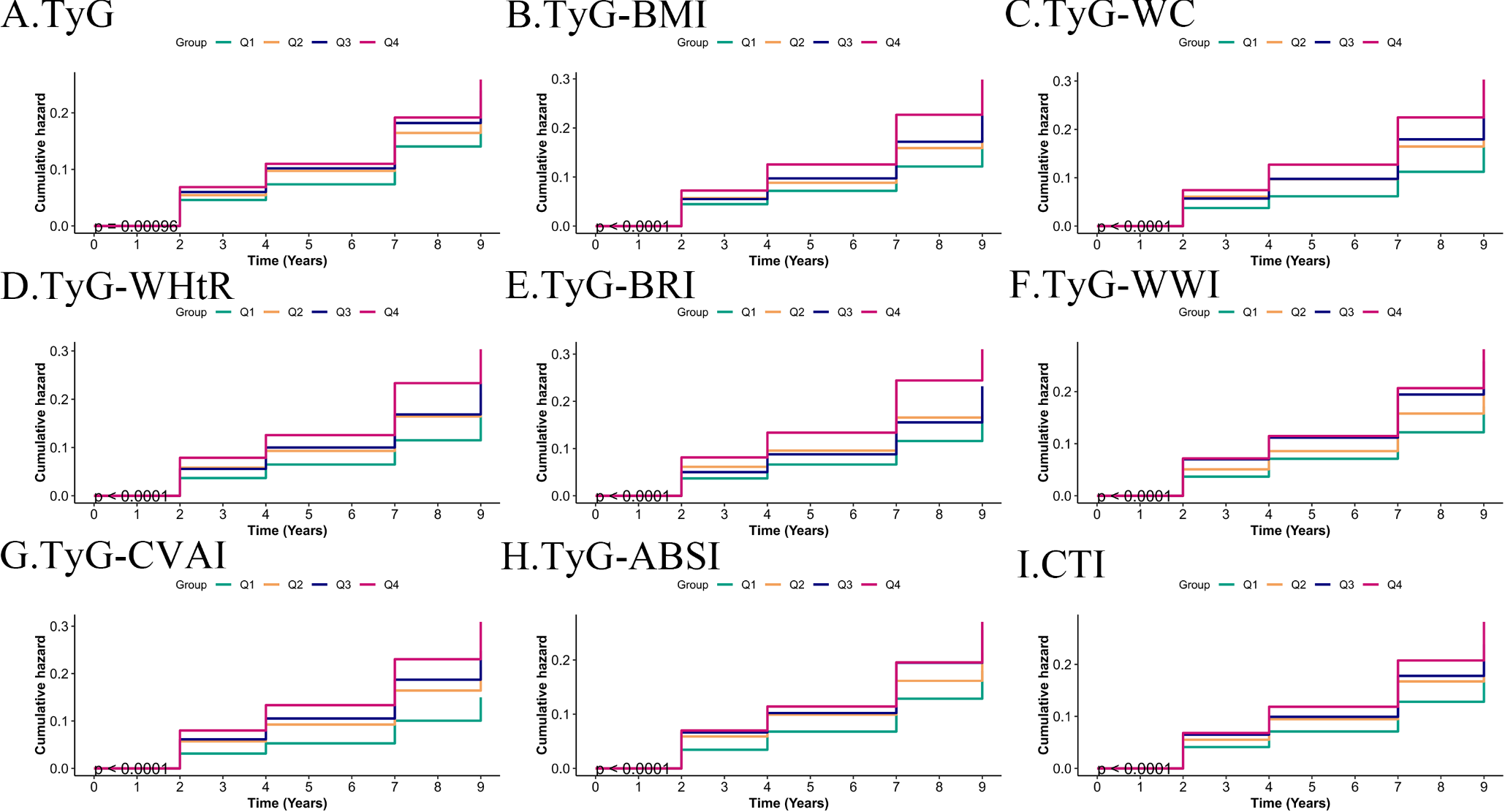
**

**Fig.S4** Kaplan-Meier curves for cumulative incidence of CHD by quartiles of TyG-related indices. A. TyG, triglyceride-glucose index; B. TyG-BMI, triglyceride glucose-body mass index; C. TyG-WC, triglyceride glucose-waist circumference; D. TyG-WHtR, triglyceride glucose-waist height ratio index; E. TyG-BRI, triglyceride glucose-body roundness index; F. TyG-WWI, triglyceride glucose-weight-adjusted waist index; G. TyG-CVAI, triglyceride glucose-Chinese visceral adiposity index; H. TyG-ABSI, triglyceride glucose-a body shape index; I. CTI, C-reactive protein-triglyceride-glucose index. CHD, coronary heart disease


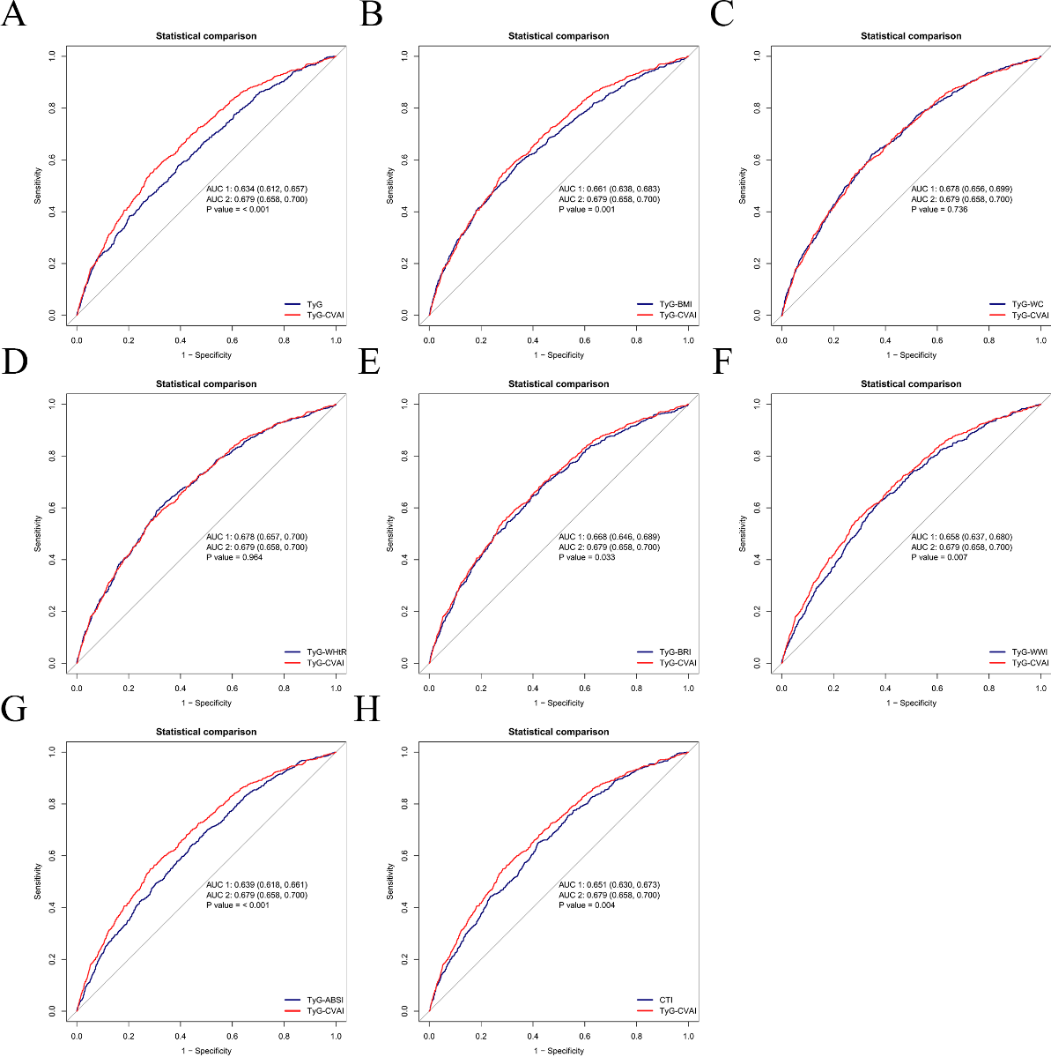


**Fig.S5** Pairwise comparison of the AUC for predicting CMM among TyG-related indices using the DeLong test. A. TyG vs. TyG-CVAI; B. TyG-BMI vs. TyG-CVAI; C. TyG-WC vs. TyG-CVAI; D. TyG-WHtR vs. TyG-CVAI; E. TyG-BRI vs. TyG-CVAI; F. TyG-WWI vs. TyG-CVAI; G. TyG-ABSI vs. TyG-CVAI; H. CTI vs. TyG-CVAI. AUC, area under curve; CMM, cardiometabolic multimorbidity; TyG, triglyceride-glucose index; TyG-BMI, triglyceride glucose-body mass index; TyG-WC, triglyceride glucose-waist circumference; TyG-WHtR, triglyceride glucose-waist height ratio index; TyG-BRI, triglyceride glucose-body roundness index; TyG-WWI, triglyceride glucose-weight-adjusted waist index; TyG-CVAI, triglyceride glucose-Chinese visceral adiposity index; TyG-ABSI, triglyceride glucose-a body shape index; CTI, C-reactive protein-triglyceride-glucose index
